# Methodological quality (risk of bias) assessment tools for primary and secondary medical studies: what are they and which is better?

**DOI:** 10.1186/s40779-020-00238-8

**Published:** 2020-02-29

**Authors:** Lin-Lu Ma, Yun-Yun Wang, Zhi-Hua Yang, Di Huang, Hong Weng, Xian-Tao Zeng

**Affiliations:** 1 Center for Evidence-Based and Translational Medicine, Zhongnan Hospital, Wuhan University, 169 Donghu Road, Wuchang District, Wuhan, 430071 Hubei China; 2 Department of Evidence-Based Medicine and Clinical Epidemiology, The Second Clinical College, Wuhan University, Wuhan, 430071 China; 3 Center for Evidence-Based and Translational Medicine, Wuhan University, Wuhan, 430071 China; 4 Global Health Institute, Wuhan University, Wuhan, 430072 China

**Keywords:** Methodological quality, Risk of bias, Quality assessment, Critical appraisal, Methodology checklist, Appraisal tool, Observational study, Qualitative study, Interventional study, Outcome measurement instrument

## Abstract

Methodological quality (risk of bias) assessment is an important step before study initiation usage. Therefore, accurately judging study type is the first priority, and the choosing proper tool is also important. In this review, we introduced methodological quality assessment tools for randomized controlled trial (including individual and cluster), animal study, non-randomized interventional studies (including follow-up study, controlled before-and-after study, before-after/ pre-post study, uncontrolled longitudinal study, interrupted time series study), cohort study, case-control study, cross-sectional study (including analytical and descriptive), observational case series and case reports, comparative effectiveness research, diagnostic study, health economic evaluation, prediction study (including predictor finding study, prediction model impact study, prognostic prediction model study), qualitative study, outcome measurement instruments (including patient - reported outcome measure development, content validity, structural validity, internal consistency, cross-cultural validity/ measurement invariance, reliability, measurement error, criterion validity, hypotheses testing for construct validity, and responsiveness), systematic review and meta-analysis, and clinical practice guideline. The readers of our review can distinguish the types of medical studies and choose appropriate tools. In one word, comprehensively mastering relevant knowledge and implementing more practices are basic requirements for correctly assessing the methodological quality.

## Background

In the twentieth century, pioneering works by distinguished professors Cochrane A [1], Guyatt GH [2], and Chalmers IG [3] have led us to the evidence-based medicine (EBM) era. In this era, how to search, critically appraise, and use the best evidence is important. Moreover, systematic review and meta-analysis is the most used tool for summarizing primary data scientifically [4–6] and also the basic for developing clinical practice guideline according to the Institute of Medicine (IOM) [7]. Hence, to perform a systematic review and/ or meta-analysis, assessing the methodological quality of based primary studies is important; naturally, it would be key to assess its own methodological quality before usage. Quality includes internal and external validity, while methodological quality usually refers to internal validity [8, 9]. Internal validity is also recommended as “risk of bias (RoB)” by the Cochrane Collaboration [9].

There are three types of tools: scales, checklists, and items [10, 11]. In 2015, Zeng et al. [11] investigated methodological quality tools for randomized controlled trial (RCT), non-randomized clinical intervention study, cohort study, case-control study, cross-sectional study, case series, diagnostic accuracy study which also called “diagnostic test accuracy (DTA)”, animal study, systematic review and meta-analysis, and clinical practice guideline (CPG). From then on, some changes might generate in pre-existing tools, and new tools might also emerge; moreover, the research method has also been developed in recent years. Hence, it is necessary to systematically investigate commonly-used tools for assessing methodological quality, especially those for economic evaluation, clinical prediction rule/model, and qualitative study. Therefore, this narrative review presented related methodological quality (including “RoB”) assessment tools for primary and secondary medical studies up to December 2019, and Table 1 presents their basic characterizes. We hope this review can help the producers, users, and researchers of evidence.

**Table 1:** The basic characteristics of the included methodological quality (risk of bias) assessment tools

No.	Development Organization	Tool’s name	Type of study
1	The Cochrane Collaboration	Cochrane RoB tool and RoB 2.0 tool	Randomized controlled trial Diagnostic accuracy study
2	The Physiotherapy Evidence Database (PEDro)	PEDro scale	Randomized controlled trial
3	The Effective Practice and Organisation of Care (EPOC) Group	EPOC RoB tool	Randomized controlled trial Clinical controlled trials Controlled before-and-after study Interrupted time series studies
4	The Critical Appraisal Skills Programme (CASP)	CASP checklist	Randomized controlled trial Cohort study Case-control study Cross-sectional study Diagnostic test study Clinical prediction rule Economic evaluation Qualitative study Systematic review
5	The National Institutes of Health (NIH)	NIH quality assessment tool	Controlled intervention study Cohort study Cross-sectional study Case-control study Before-after (Pre-post) study with no control group Case-series (Interventional) Systematic review and meta-analysis
6	The Joanna Briggs Institute (JBI)	JBI critical appraisal checklist	Randomized controlled trial Non-randomized experimental study Cohort study Case-control study Cross-sectional study Prevalence data Case reports Economic evaluation Qualitative study Text and expert opinion papers Systematic reviews and research syntheses
7	The Scottish Intercollegiate Guidelines Network (SIGN)	SIGN methodology checklist	Randomized controlled trial Cohort study Case-control study Diagnostic study Economic evaluation Systematic reviews and meta-analyses
8	The Stroke Therapy Academic Industry Roundtable (STAIR) Group	CAMARADES tool	Animal study
9	The SYstematic Review Center for Laboratory animal Experimentation (SYRCLE)	SYRCLE’s RoB tool	Animal study
10	Sterne JAC et al.	ROBINS-I tool	Non-randomised interventional study
11	Slim K et al.	MINORS tool	Non-randomised interventional study
12	The Canada Institute of Health Economics (IHE)	IHE quality appraisal tool	Case-series (Interventional)
13	Wells GA et al.	Newcastle-Ottawa Scale (NOS)	Cohort study Case-control study
14	Downes MJ et al.	AXIS tool	Cross-sectional study
15	The Agency for Healthcare Research and Quality (AHRQ)	AHRQ methodology checklist	Cross-sectional/ Prevalence study
16	Crombie I	Crombie’s items	Cross-sectional study
17	The Good Research for Comparative Effectiveness (GRACE) Initiative	GRACE checklist	Comparative effectiveness research
18	Whiting PF et al.	QUADAS tool and QUADAS-2 tool	Diagnostic accuracy study
19	The National Institute for Clinical Excellence (NICE)	NICE methodology checklist	Economic evaluation
20	The Cabinet Office	The Quality Framework: Cabinet Office checklist	Qualitative study (social research)
21	Hayden JA et al.	QIPS tool	Prediction study (predictor finding study)
22	Wolff RF et al.	PROBAST	Prediction study (prediction model study)
23	The (COnsensus-based Standards for the selection of health Measurement INstruments) initiative	COSMIN RoB checklist	Patient-reported outcome measure development Content validity Structural validity Internal consistency Cross-cultural validity/ measurement invariance Reliability Measurement error Criterion validity Hypotheses testing for construct validity Responsiveness
24	Shea BJ et al.	AMSTAR and AMSTAR 2	Systematic review
25	The Decision Support Unit (DSU)	DSU network meta-analysis (NMA) methodology checklist	Network meta-analysis
26	Whiting P et al.	ROBIS tool	Systematic review
27	Brouwers MC et al.	AGREE instrument and AGREE II instrument	Clinical practice guideline

*AMSTAR* A measurement tool to assess systematic reviews, *AHRQ* Agency for healthcare research and quality, *AXIS* Appraisal tool for cross-sectional studies, *CASP* Critical appraisal skills programme, *CAMARADES* The collaborative approach to meta-analysis and review of animal data from experimental studies, *COSMIN* Consensus-based standards for the selection of health measurement instruments, *DSU* Decision support unit, *EPOC* the effective practice and organisation of care group, *GRACE* The god research for comparative effectiveness initiative, *IHE* Canada institute of health economics, *JBI* Joanna Briggs Institute, *MINORS* Methodological index for non-randomized studies, *NOS* Newcastle-Ottawa scale, *NMA* network meta-analysis, *NIH* national institutes of health, *NICE* National institute for clinical excellence, *PEDro* physiotherapy evidence database, *PROBAST* The prediction model risk of bias assessment tool, *QUADAS* Quality assessment of diagnostic accuracy studies, *QIPS* Quality in prognosis studies, *RoB* Risk of bias, *ROBINS-I* Risk of bias in non-randomised studies - of interventions, *ROBIS* Risk of bias in systematic review, *SYRCLE* Systematic review center for laboratory animal experimentation, *STAIR* Stroke therapy academic industry roundtable, *SIGN* The Scottish intercollegiate guidelines network

## Tools for intervention studies

### Randomized controlled trial (individual or cluster)

The first RCT was designed by Hill BA (1897–1991) and became the “gold standard” for experimental study design [12, 13] up to now. Nowadays, the Cochrane risk of bias tool for randomized trials (which was introduced in 2008 and edited on March 20, 2011) is the most commonly recommended tool for RCT [9, 14], which is called “RoB”. On August 22, 2019 (which was introduced in 2016), the revised revision for this tool to assess RoB in randomized trials (RoB 2.0) was published [15]. The RoB 2.0 tool is suitable for individually-randomized, parallel-group, and cluster- randomized trials, which can be found in the dedicated website https://www.riskofbias.info/welcome/rob-2-0-tool. The RoB 2.0 tool consists of five bias domains and shows major changes when compared to the original Cochrane RoB tool (Table S1A-B presents major items of both versions).

The Physiotherapy Evidence Database (PEDro) scale is a specialized methodological assessment tool for RCT in physiotherapy [16, 17] and can be found in http://www.pedro.org.au/english/downloads/pedro-scale/, covering 11 items (Table S1C). The Effective Practice and Organisation of Care (EPOC) Group is a Cochrane Review Group who also developed a tool (called as “EPOC RoB Tool”) for complex interventions randomized trials. This tool has 9 items (Table S1D) and can be found in https://epoc.cochrane.org/resources/epoc-resources-review-authors. The Critical Appraisal Skills Programme (CASP) is a part of the Oxford Centre for Triple Value Healthcare Ltd. (3 V) portfolio, which provides resources and learning and development opportunities to support the development of critical appraisal skills in the UK (http://www.casp-uk.net/) [18–20]. The CASP checklist for RCT consists of three sections involving 11 items (Table S1E). The National Institutes of Health (NIH) also develops quality assessment tools for controlled intervention study (Table S1F) to assess methodological quality of RCT (https://www.nhlbi.nih.gov/health-topics/study-quality-assessment-tools).

The Joanna Briggs Institute (JBI) is an independent, international, not-for-profit researching and development organization based in the Faculty of Health and Medical Sciences at the University of Adelaide, South Australia (https://joannabriggs.org/). Hence, it also develops many critical appraisal checklists involving the feasibility, appropriateness, meaningfulness and effectiveness of healthcare interventions. Table S1G presents the JBI Critical appraisal checklist for RCT, which includes 13 items.

The Scottish Intercollegiate Guidelines Network (SIGN) was established in 1993 (https://www.sign.ac.uk/). Its objective is to improve the quality of health care for patients in Scotland via reducing variations in practices and outcomes, through developing and disseminating national clinical guidelines containing recommendations for effective practice based on current evidence. Hence, it also develops many critical appraisal checklists for assessing methodological quality of different study types, including RCT (Table S1H).

In addition, the Jadad Scale [21], Modified Jadad Scale [22, 23], Delphi List [24], Chalmers Scale [25], National Institute for Clinical Excellence (NICE) methodology checklist [11], Downs & Black checklist [26], and other tools summarized by West et al. in 2002 [27] are not commonly used or recommended nowadays.

### Animal study

Before starting clinical trials, the safety and effectiveness of new drugs are usually tested in animal models [28], so animal study is considered as preclinical research, possessing important significance [29, 30]. Likewise, the methodological quality of animal study also needs to be assessed [30]. In 1999, the initial “Stroke Therapy Academic Industry Roundtable (STAIR)” recommended their criteria for assessing the quality of stroke animal studies [31] and this tool is also called “STAIR”. In 2009, the STAIR Group updated their criteria and developed “Recommendations for Ensuring Good Scientific Inquiry” [32]. Besides, Macleod et al. [33] proposed a 10-point tool based on STAIR to assess methodological quality of animal study in 2004, which is also called “CAMARADES (The Collaborative Approach to Meta-Analysis and Review of Animal Data from Experimental Studies)”; with “S” presenting “Stroke” at that time and now standing for “Studies” (http://www.camarades.info/). In CAMARADES tool, every item could reach a highest score of one point and the total score for this tool could achieve 10 points (Table S1J).

In 2008, the Systematic Review Center for Laboratory animal Experimentation (SYRCLE) was established in Netherlands and this team developed and released an RoB tool for animal intervention studies - SYRCLE’s RoB tool in 2014, based on the original Cochrane RoB Tool [34]. This new tool contained 10 items which had become the most recommended tool for assessing the methodological quality of animal intervention studies (Table S1I).

### Non-randomised studies

In clinical research, RCT is not always feasible [35]; therefore, non-randomized design remains considerable. In non-randomised study (also called quasi-experimental studies), investigators control the allocation of participants into groups, but do not attempt to adopt randomized operation [36], including follow-up study. According to with or without comparison, non-randomized clinical intervention study can be divided into comparative and non-comparative sub-types, the Risk Of Bias In Non-randomised Studies - of Interventions (ROBINS-I) tool [37] is the preferentially recommended tool. This tool is developed to evaluate risk of bias in estimating comparative effectiveness (harm or benefit) of interventions in studies not adopting randomization in allocating units (individuals or clusters of individuals) into comparison groups. Besides, the JBI critical appraisal checklist for quasi-experimental studies (non-randomized experimental studies) is also suitable, which includes 9 items. Moreover, the methodological index for non-randomized studies (MINORS) [38] tool can also be used, which contains a total of 12 methodological points; the first 8 items could be applied for both non-comparative and comparative studies, while the last 4 items appropriate for studies with two or more groups. Every item is scored from 0 to 2, and the total scores over 16 or 24 give an overall quality score. Table S1K-L-M presented the major items of these three tools.

Non-randomized study with a separate control group could also be called clinical controlled trial or controlled before-and-after study. For this design type, the EPOC RoB tool is suitable (see Table S1D). When using this tool, the “random sequence generation” and “allocation concealment” should be scored as “High risk”, while grading for other items could be the same as that for randomized trial.

Non-randomized study without a separate control group could be a before-after (Pre-Post) study, a case series (uncontrolled longitudinal study), or an interrupted time series study. A case series is described a series of individuals, who usually receive the same intervention, and contains non control group [9]. There are several tools for assessing the methodological quality of case series study. The latest one was developed by Moga C et al. [39] in 2012 using a modified Delphi technique, which was developed by the Canada Institute of Health Economics (IHE); hence, it is also called “IHE Quality Appraisal Tool” (Table S1N). Moreover, NIH also develops a quality assessment tool for case series study, including 9 items (Table S1O). For interrupted time series studies, the “EPOC RoB tool for interrupted time series studies” is recommended (Table S1P). For the before-after study, we recommend the NIH quality assessment tool for before-after (Pre-Post) study without control group (Table S1Q).

In addition, for non-randomized intervention study, the Reisch tool (Check List for Assessing Therapeutic Studies) [11, 40], Downs & Black checklist [26], and other tools summarized by Deeks et al. [36] are not commonly used or recommended nowadays.

## Tools for observational studies and diagnostic study

Observational studies include cohort study, case-control study, cross-sectional study, case series, case reports, and comparative effectiveness research [41], and can be divided into analytical and descriptive studies [42].

### Cohort study

Cohort study includes prospective cohort study, retrospective cohort study, and ambidirectional cohort study [43]. There are some tools for assessing the quality of cohort study, such as the CASP cohort study checklist (Table S2A), SIGN critical appraisal checklists for cohort study (Table S2B), NIH quality assessment tool for observational cohort and cross-sectional studies (Table S2C), Newcastle-Ottawa Scale (NOS; Table S2D) for cohort study, and JBI critical appraisal checklist for cohort study (Table S2E). However, the Downs & Black checklist [26] and the NICE methodology checklist for cohort study [11] are not commonly used or recommended nowadays.

The NOS [44, 45] came from an ongoing collaboration between the Universities of Newcastle, Australia and Ottawa, Canada. Among all above mentioned tools, the NOS is the most commonly used tool nowadays which also allows to be modified based on a special subject.

### Case-control study

Case-control study selects participants based on the presence of a specific disease or condition, and seeks earlier exposures that may lead to the disease or outcome [42]. It has an advantage over cohort study, that is the issue of “drop out” or “loss in follow up” of participants as seen in cohort study would not arise in such study. Nowadays, there are some acceptable tools for assessing the methodological quality of case-control study, including CASP case-control study checklist (Table S2F), SIGN critical appraisal checklists for case-control study (Table S2G), NIH quality assessment tool of case-control study (Table S2H), JBI critical appraisal checklist for case-control study (Table S2I), and the NOS for case-control study (Table S2J). Among them, the NOS for case-control study is also the most frequently used tool nowadays and allows to be modified by users.

In addition, the Downs & Black checklist [26] and the NICE methodology checklist for case-control study [11] are also not commonly used or recommended nowadays.

### Cross-sectional study (analytical or descriptive)

Cross-sectional study is used to provide a snapshot of a disease and other variables in a defined population at a time point. It can be divided into analytical and purely descriptive types. Descriptive cross-sectional study merely describes the number of cases or events in a particular population at a time point or during a period of time; whereas analytic cross-sectional study can be used to infer relationships between a disease and other variables [46].

For assessing the quality of analytical cross-sectional study, the NIH quality assessment tool for observational cohort and cross-sectional studies (Table S2C), JBI critical appraisal checklist for analytical cross-sectional study (Table S2K), and the Appraisal tool for Cross-Sectional Studies (AXIS tool; Table S2L) [47] are recommended tools. The AXIS tool is a critical appraisal tool that addresses study design and reporting quality as well as the risk of bias in cross-sectional study, which was developed in 2016 and contains 20 items. Among these three tools, the JBI checklist is the most preferred one.

Purely descriptive cross-sectional study is usually used to measure disease prevalence and incidence. Hence, the critical appraisal tool for analytic cross-sectional study is not proper for the assessment. Only few quality assessment tools are suitable for descriptive cross-sectional study, like the JBI critical appraisal checklist for studies reporting prevalence data [48] (Table S2M), Agency for Healthcare Research and Quality (AHRQ) methodology checklist for assessing the quality of cross-sectional/ prevalence study (Table S2N), and Crombie’s items for assessing the quality of cross-sectional study [49] (Table S2O). Among them, the JBI tool is the newest.

### Case series and case reports

Unlike above mentioned interventional case series, case reports and case series are used to report novel occurrences of a disease or a unique finding [50]. Hence, they belong to descriptive studies. There is only one tool – the JBI critical appraisal checklist for case reports (Table S2P).

### Comparative effectiveness research

Comparative effectiveness research (CER) compares real-world outcomes [51] resulting from alternative treatment options that are available for a given medical condition. Its key elements include the study of effectiveness (effect in the real world), rather than efficacy (ideal effect), and the comparisons among alternative strategies [52]. In 2010, the Good Research for Comparative Effectiveness (GRACE) Initiative was established and developed principles to help healthcare providers, researchers, journal readers, and editors evaluate inherent quality for observational research studies of comparative effectiveness [41]. And in 2016, a validated assessment tool – the GRACE Checklist v5.0 (Table S2Q) was released for assessing the quality of CER.

### Diagnostic study

Diagnostic tests, also called “Diagnostic Test Accuracy (DTA)”, are used by clinicians to identify whether a condition exists in a patient or not, so as to develop an appropriate treatment plan [53]. DTA has several unique features in terms of its design which differ from standard intervention and observational evaluations. In 2003, Penny et al. [53, 54] developed a tool for assessing the quality of DTA, namely Quality Assessment of Diagnostic Accuracy Studies (QUADAS) tool. In 2011, a revised “QUADAS-2” tool (Table S2R) was launched [55, 56]. Besides, the CASP diagnostic checklist (Table S2S), SIGN critical appraisal checklists for diagnostic study (Table S2T), JBI critical appraisal checklist for diagnostic test accuracy studies (Table S2U), and the Cochrane risk of bias assessing tool for diagnostic test accuracy (Table S2V) are also common useful tools in this field.

Of them, the Cochrane risk of bias tool (https://methods.cochrane.org/sdt/) is based on the QUADAS tool, and the SIGN and JBI tools are based on the QUADAS-2 tool. Of course, the QUADAS-2 tool is the first recommended tool. Other relevant tools reviewed by Whiting et al. [53] in 2004 are not used nowadays.

## Tools for other primary medical studies

### Health economic evaluation

Health economic evaluation research comparatively analyses alternative interventions with regard to their resource uses, costs and health effects [57]. It focuses on identifying, measuring, valuing and comparing resource use, costs and benefit/effect consequences for two or more alternative intervention options [58]. Nowadays, health economic study is increasingly popular. Of course, its methodological quality also needs to be assessed before its initiation. The first tool for such assessment was developed by Drummond and Jefferson in 1996 [59], and then many tools have been developed based on the Drummond’s items or its revision [60], such as the SIGN critical appraisal checklists for economic evaluations (Table S3A), CASP economic evaluation checklist (Table S3B), and the JBI critical appraisal checklist for economic evaluations (Table S3C). The NICE only retains one methodology checklist for economic evaluation (Table S3D).

However, we regard the Consolidated Health Economic Evaluation Reporting Standards (CHEERS) statement [61] as a reporting tool rather than a methodological quality assessment tool, so we do not recommend it to assess the methodological quality of health economic evaluation.

### Qualitative study

In healthcare, qualitative research aims to understand and interpret individual experiences, behaviours, interactions, and social contexts, so as to explain interested phenomena, such as the attitudes, beliefs, and perspectives of patients and clinicians; the interpersonal nature of caregiver and patient relationships; illness experience; and the impact of human sufferings [62]. Compared with quantitative studies, assessment tools for qualitative studies are fewer. Nowadays, the CASP qualitative research checklist (Table S3E) is the most frequently recommended tool for this issue. Besides, the JBI critical appraisal checklist for qualitative research [63, 64] (Table S3F) and the Quality Framework: Cabinet Office checklist for social research [65] (Table S3G) are also suitable.

### Prediction studies

Clinical prediction study includes predictor finding (prognostic factor) studies, prediction model studies (development, validation, and extending or updating), and prediction model impact studies [66]. For predictor finding study, the Quality In Prognosis Studies (QIPS) tool [67] can be used for assessing its methodological quality (Table S3H). For prediction model impact studies, if it uses a randomized comparative design, tools for RCT can be used, especially the RoB 2.0 tool; if it uses a nonrandomized comparative design, tools for non-randomized studies can be used, especially the ROBINS-I tool. For diagnostic and prognostic prediction model studies, the Prediction model Risk Of Bias Assessment Tool (PROBAST; Table S3I) [68] and CASP clinical prediction rule checklist (Table S3J) are suitable.

### Text and expert opinion papers

Text and expert opinion-based evidence (also called “non-research evidence”) comes from expert opinions, consensus, current discourse, comments, and assumptions or assertions that appear in various journals, magazines, monographs and reports [69–71]. Nowadays, only the JBI has a critical appraisal checklist for the assessment of text and expert opinion papers (Table S3K).

### Outcome measurement instruments

An outcome measurement instrument is a “device” used to collect a measurement. The range embraced by the term ‘instrument’ is broad, and can refer to questionnaire (e.g. patient-reported outcome such as quality of life), observation (e.g. the result of a clinical examination), scale (e.g. a visual analogue scale), laboratory test (e.g. blood test) and images (e.g. ultrasound or other medical imaging) [72, 73]. Measurements can be subjective or objective, and either unidimensional (e.g. attitude) or multidimensional. Nowadays, only one tool - the COnsensus-based Standards for the selection of health Measurement INstruments (COSMIN) Risk of Bias checklist [74–76] (www.cosmin.nl/) is proper for assessing the methodological quality of outcome measurement instrument, and Table S3L presents its major items, including patient - reported outcome measure (PROM) development (Table S3LA), content validity (Table S3LB), structural validity (Table S3LC), internal consistency (Table S3LD), cross-cultural validity/ measurement invariance (Table S3LE), reliability (Table S3LF), measurement error (Table S3LG), criterion validity (Table S3LH), hypotheses testing for construct validity (Table S3LI), and responsiveness (Table S3LJ).

## Tools for secondary medical studies

### Systematic review and meta-analysis

Systematic review and meta-analysis are popular methods to keep up with current medical literature [4–6]. Their ultimate purposes and values lie in promoting healthcare [6, 77, 78]. Meta-analysis is a statistical process of combining results from several studies, commonly a part of a systematic review [11]. Of course, critical appraisal would be necessary before using systematic review and meta-analysis.

In 1988, Sacks et al. developed the first tool for assessing the quality of meta-analysis on RCTs - the Sack’s Quality Assessment Checklist (SQAC) [79]; And then in 1991, Oxman and Guyatt developed another tool – the Overview Quality Assessment Questionnaire (OQAQ) [80, 81]. To overcome the shortcomings of these two tools, in 2007 the A Measurement Tool to Assess Systematic Reviews (AMSTAR) was developed based on them [82] (http://www.amstar.ca/). However, this original AMSTAR instrument did not include an assessment on the risk of bias for non-randomised studies, and the expert group thought revisions should address all aspects of the conduct of a systematic review. Hence, the new instrument for randomised or non-randomised studies on healthcare interventions - AMSTAR 2 was released in 2017 [83], and Table S4A presents its major items.

Besides, the CASP systematic review checklist (Table S4B), SIGN critical appraisal checklists for systematic reviews and meta-analyses (Table S4C), JBI critical appraisal checklist for systematic reviews and research syntheses (Table S4D), NIH quality assessment tool for systematic reviews and meta-analyses (Table S4E), The Decision Support Unit (DSU) network meta-analysis (NMA) methodology checklist (Table S4F), and the Risk of Bias in Systematic Review (ROBIS) [84] tool (Table S4G) are all suitable. Among them, the AMSTAR 2 is the most commonly used and the ROIBS is the most frequently recommended.

Among those tools, the AMSTAR 2 is suitable for assessing systematic review and meta-analysis based on randomised or non-randomised interventional studies, the DSU NMA methodology checklist for network meta-analysis, while the ROBIS for meta-analysis based on interventional, diagnostic test accuracy, clinical prediction, and prognostic studies.

### Clinical practice guidelines

Clinical practice guideline (CPG) is integrated well into the thinking of practicing clinicians and professional clinical organizations [85–87]; and also make scientific evidence incorporated into clinical practice [88]. However, not all CPGs are evidence-based [89, 90] and their qualities are uneven [91–93]. Until now there were more than 20 appraisal tools have been developed [94]. Among them, the Appraisal of Guidelines for Research and Evaluation (AGREE) instrument has the greatest potential in serving as a basis to develop an appraisal tool for clinical pathways [94]. The AGREE instrument was first released in 2003 [95] and updated to AGREE II instrument in 2009 [96] (www.agreetrust.org/). Now the AGREE II instrument is the most recommended tool for CPG (Table S4H).

Besides, based on the AGREE II, the AGREE Global Rating Scale (AGREE GRS) Instrument [97] was developed as a short item tool to evaluate the quality and reporting of CPGs.

## Discussion and conclusions

Currently, the EBM is widely accepted and the major attention of healthcare workers lies in “Going from evidence to recommendations” [98, 99]. Hence, critical appraisal of evidence before using is a key point in this process [100, 101]. In 1987, Mulrow CD [102] pointed out that medical reviews needed routinely use scientific methods to identify, assess, and synthesize information. Hence, perform methodological quality assessment is necessary before using the study. However, although there are more than 20 years have been passed since the first tool emergence, many users remain misunderstand the methodological quality and reporting quality. Of them, someone used the reporting checklist to assess the methodological quality, such as used the Consolidated Standards of Reporting Trials (CONSORT) statement [103] to assess methodological quality of RCT, used the Strengthening the Reporting of Observational Studies in Epidemiology (STROBE) statement [104] to methodological quality of cohort study. This phenomenon indicates more universal education of clinical epidemiology is needed for medical students and professionals.

The methodological quality tool development should according to the characteristics of different study types. In this review, we used “methodological quality”, “risk of bias”, “critical appraisal”, “checklist”, “scale”, “items”, and “assessment tool” to search in the NICE website, SIGN website, Cochrane Library website and JBI website, and on the basis of them, added “systematic review”, “meta-analysis”, “overview” and “clinical practice guideline” to search in PubMed. Compared with our previous systematic review [11], we found some tools are recommended and remain used, some are used without recommendation, and some are eliminated [10, 29, 30, 36, 53, 94, 105–107]. These tools produce a significant impetus for clinical practice [108, 109].

In addition, compared with our previous systematic review [11], this review stated more tools, especially those developed after 2014, and the latest revisions. Of course, we also adjusted the method of study type classification. Firstly, in 2014, the NICE provided 7 methodology checklists but only retains and updated the checklist for economic evaluation now. Besides, the Cochrane RoB 2.0 tool, AMSTAR 2 tool, CASP checklist, and most of JBI critical appraisal checklists are all the newest revisions; the NIH quality assessment tool, ROBINS-I tool, EPOC RoB tool, AXIS tool, GRACE Checklist, PROBAST, COSMIN Risk of Bias checklist, and ROBIS tool are all newly released tools. Secondly, we also introduced tools for network meta-analysis, outcome measurement instruments, text and expert opinion papers, prediction studies, qualitative study, health economic evaluation, and CER. Thirdly, we classified interventional studies into randomized and non-randomized sub-types, and then further classified non-randomized studies into with and without controlled group. Moreover, we also classified cross-sectional study into analytic and purely descriptive sub-types, and case-series into interventional and observational sub-types. These processing courses were more objective and comprehensive.

Obviously, the number of appropriate tools is the largest for RCT, followed by cohort study; the applicable range of JBI is widest [63, 64], with CASP following closely. However, further efforts remain necessary to develop appraisal tools. For some study types, only one assessment tool is suitable, such as CER, outcome measurement instruments, text and expert opinion papers, case report, and CPG. Besides, there is no proper assessment tool for many study types, such as overview, genetic association study, and cell study. Moreover, existing tools have not been fully accepted. In the future, how to develop well accepted tools remains a significant and important work [11].

Our review can help the professionals of systematic review, meta-analysis, guidelines, and evidence users to choose the best tool when producing or using evidence. Moreover, methodologists can obtain the research topics for developing new tools. Most importantly, we must remember that all assessment tools are subjective, and actual yields of wielding them would be influenced by user’s skills and knowledge level. Therefore, users must receive formal training (relevant epidemiological knowledge is necessary), and hold rigorous academic attitude, and at least two independent reviewers should be involved in evaluation and cross-checking to avoid performance bias [110].

## Supplementary information

**Additional file 1: Table S1.** Major components of the tools for assessing intervention studies

**Additional file 2: Table S2.** Major components of the tools for assessing observational studies and diagnostic study

**Additional file 3: Table S3.** Major components of the tools for assessing other primary medical studies

**Additional file 4: Table S4.** Major components of the tools for assessing secondary medical studies

## Data Availability

The data and materials used during the current review are all available in this review.

## Abbreviations

AGREE GRS: AGREE Global rating scale
AGREE: Appraisal of guidelines for research and evaluation
AHRQ: Agency for healthcare research and quality
AMSTAR: A measurement tool to assess systematic reviews
AXIS: Appraisal tool for cross-sectional studies
CAMARADES: The collaborative approach to meta-analysis and review of animal data from experimental studies
CASP: Critical appraisal skills programme
CER: Comparative effectiveness research
CHEERS: Consolidated health economic evaluation reporting standards
CONSORT: Consolidated standards of reporting trials
COSMIN: Consensus-based standards for the selection of health measurement instruments
CPG: Clinical practice guideline
DSU: Decision support unit
DTA: Diagnostic test accuracy
EBM: Evidence-based medicine
EPOC: The effective practice and organisation of care group
GRACE: The good research for comparative effectiveness initiative
IHE: Canada institute of health economics
IOM: Institute of medicine
JBI: Joanna Briggs Institute
MINORS: Methodological index for non-randomized studies
NICE: National institute for clinical excellence
NIH: National institutes of health
NMA: Network meta-analysis
NOS: Newcastle-Ottawa scale
OQAQ: Overview quality assessment questionnaire
PEDro: Physiotherapy evidence database
PROBAST: The prediction model risk of bias assessment tool
PROM: Patient - reported outcome measure
QIPS: Quality in prognosis studies
QUADAS: Quality assessment of diagnostic accuracy studies
RCT: Randomized controlled trial
RoB: Risk of bias
ROBINS-I: Risk of bias in non-randomised studies - of interventions
ROBIS: Risk of bias in systematic review
SIGN: The Scottish intercollegiate guidelines network
SQAC: Sack’s quality assessment checklist
STAIR: Stroke therapy academic industry roundtable
STROBE: Strengthening the reporting of observational studies in epidemiology
SYRCLE: Systematic review center for laboratory animal experimentation
