# Supplementary material for: Methodological quality (risk of bias) assessment tools for primary and secondary medical studies: what are they and which is better?
Source: Mil Med Res. 2020 Feb 29;7:7. doi: 10.1186/s40779-020-00238-8 (PMC7049186; doi:10.1186/s40779-020-00238-8)
Supplement: Supplementary file 1 — Additional file 1: Table S1. Major components of the tools for assessing intervention studies [file 40779_2020_238_MOESM1_ESM.docx]

**Table S1. Major components of the tools for assessing intervention studies**

| **A. The Cochrane RoB 2.0 tool (introduced in 2016 and last edited on August 22, 2019)**  **Website:** https://www.riskofbias.info/ | | | | | | | | | | | | | | | | | | | | | | | | | | | | | |
| --- | --- | --- | --- | --- | --- | --- | --- | --- | --- | --- | --- | --- | --- | --- | --- | --- | --- | --- | --- | --- | --- | --- | --- | --- | --- | --- | --- | --- | --- |
| Bias domain and signalling question (Signalling questions for bias due to deviations from intended interventions relate to the effect of assignment to intervention) | | | | | | | Response options | | | | | | | | | | | | | | | | | | | | | | |
|  |  |  |  |  |  |  | Lower risk of bias | | | | | | | | | | | Higher risk of bias | | | | | | | | Other | | | |
| **1. Bias arising from the randomisation process** | | | | | | | | | | | | | | | | | | | | | | | | | | | | | |
| 1.1 Was the allocation sequence random? | | | | | | | Yes/ Probably Yes | | | | | | | | | | | No/ Probably No | | | | | | | | No Information | | | |
| 1.2 Was the allocation sequence concealed until participants were enrolled and assigned to interventions? | | | | | | | Yes/ Probably Yes | | | | | | | | | | | No/ Probably No | | | | | | | | No Information | | | |
| 1.3 Did baseline differences between intervention groups suggest a problem with the randomisation process? | | | | | | | No/ Probably No | | | | | | | | | | | Yes/ Probably Yes | | | | | | | | No Information | | | |
| Risk-of-bias judgment (low/high/some concerns) | | | | | | |  | | | | | | | | | | | | | | | | | | | | | | |
| Optional: What is the predicted direction of bias arising from the randomisation process? | | | | | | |  | | | | | | | | | | | | | | | | | | | | | | |
| **2. Bias due to deviations from intended interventions** | | | | | | | | | | | | | | | | | | | | | | | | | | | | | |
| 2.1 Were participants aware of their assigned intervention during the trial? | | | | | | | No/ Probably No | | | | | | | | | | | Yes/ Probably Yes | | | | | | | | No Information | | | |
| 2.2 Were carers and people delivering the interventions aware of participants’ assigned intervention during the trial? | | | | | | | No/ Probably No | | | | | | | | | | | Yes/ Probably Yes | | | | | | | | No Information | | | |
| 2.3 If Yes/ Probably Yes/ No Information to 2.1 or 2.2: Were there deviations from the intended intervention that arose because of the trial context? | | | | | | | No/ Probably No | | | | | | | | | | | Yes/ Probably Yes | | | | | | | | No Information/ Not Applicable | | | |
| 2.4 If Yes/ Probably Yes/ No Information to 2.3: Were these deviations likely to have affected the outcome? | | | | | | | No/ Probably No | | | | | | | | | | | Yes/ Probably Yes | | | | | | | | No Information/ Not Applicable | | | |
| 2.5 If Yes/ Probably Yes to 2.4: Were these deviations from intended intervention balanced between groups? | | | | | | | Yes/ Probably Yes | | | | | | | | | | | No/ Probably No | | | | | | | | No Information/ Not Applicable | | | |
| 2.6 Was an appropriate analysis used to estimate the effect of assignment to intervention? | | | | | | | Yes/ Probably Yes | | | | | | | | | | | No/ Probably No | | | | | | | | No Information | | | |
| 2.7 If No/ Probably No/ No Information to 2.6: Was there potential for a substantial impact (on the result) of the failure to analyse participants in the group to which they were randomised? | | | | | | | No/ Probably No | | | | | | | | | | | Yes/ Probably Yes | | | | | | | | No Information/ Not Applicable | | | |
| Risk-of-bias judgment (low/high/some concerns) | | | | | | |  | | | | | | | | | | | | | | | | | | | | | | |
| Optional: What is the predicted direction of bias due to deviations from intended interventions? | | | | | | |  | | | | | | | | | | | | | | | | | | | | | | |
| **3. Bias due to missing outcome data** | | | | | | | | | | | | | | | | | | | | | | | | | | | | | |
| 3.1 Were data for this outcome available for all, or nearly all, participants randomised? | | | | | | | Yes/ Probably Yes | | | | | | | | | | | No/ Probably No | | | | | | | | No Information | | | |
| 3.2 If No/ Probably No/ No Information to 3.1: Is there evidence that the result was not biased by missing outcome data? | | | | | | | Yes/ Probably Yes | | | | | | | | | | | No/ Probably No | | | | | | | | Not Applicable | | | |
| 3.3 If No/ Probably No to 3.2: Could missingness in the outcome depend on its true value? | | | | | | | No/ Probably No | | | | | | | | | | | Yes/ Probably Yes | | | | | | | | No Information/ Not Applicable | | | |
| 3.4 If Yes/ Probably Yes/ No Information to 3.3: Is it likely that missingness in the outcome depended on its true value? | | | | | | | No/ Probably No | | | | | | | | | | | Yes/ Probably Yes | | | | | | | | No Information/ Not Applicable | | | |
| Risk-of-bias judgment (low/high/some concerns) | | | | | | |  | | | | | | | | | | | | | | | | | | | | | | |
| Optional: What is the predicted direction of bias due to missing outcome data? | | | | | | |  | | | | | | | | | | | | | | | | | | | | | | |
| **4. Bias in measurement of the outcome** | | | | | | | | | | | | | | | | | | | | | | | | | | | | | |
| 4.1 Was the method of measuring the outcome inappropriate? | | | | | | | No/ Probably No | | | | | | | | | | | Yes/ Probably Yes | | | | | | | | No Information | | | |
| 4.2 Could measurement or ascertainment of the outcome have differed between intervention groups? | | | | | | | No/ Probably No | | | | | | | | | | | Yes/ Probably Yes | | | | | | | | No Information | | | |
| 4.3 If No/ Probably No/ No Information to 4.1 and 4.2: Were outcome assessors aware of the intervention received by study participants? | | | | | | | No/ Probably No | | | | | | | | | | | Yes/ Probably Yes | | | | | | | | No Information | | | |
| 4.4 If Yes/ Probably Yes/ No Information to 4.3: Could assessment of the outcome have been influenced by knowledge of intervention received? | | | | | | | No/ Probably No | | | | | | | | | | | Yes/ Probably Yes | | | | | | | | No Information/ Not Applicable | | | |
| 4.5 If Yes/ Probably Yes/ No Information to 4.4: Is it likely that assessment of the outcome was influenced by knowledge of intervention received? | | | | | | | No/ Probably No | | | | | | | | | | | Yes/ Probably Yes | | | | | | | | No Information | | | |
| Risk-of-bias judgment (low/high/some concerns) | | | | | | |  | | | | | | | | | | | | | | | | | | | | | | |
| Optional: What is the predicted direction of bias in measurement of the outcome? | | | | | | |  | | | | | | | | | | | | | | | | | | | | | | |
| **5. Bias in selection of the reported result** | | | | | | | | | | | | | | | | | | | | | | | | | | | | | |
| 5.1 Were the data that produced this result analysed in accordance with a prespecified analysis plan that was finalised before unblinded outcome data were available for analysis? | | | | | | | Yes/ Probably Yes | | | | | | | | | | | No/ Probably No | | | | | | | | No Information | | | |
| Is the numerical result being assessed likely to have been selected, on the basis of the results, from: | | | | | | |  | | | | | | | | | | |  | | | | | | | |  | | | |
| 5.2 ... multiple eligible outcome measurements (eg, scales, definitions, time points) within the outcome domain? | | | | | | | No/ Probably No | | | | | | | | | | | Yes/ Probably Yes | | | | | | | | No Information | | | |
| 5.3 ... multiple eligible analyses of the data? | | | | | | | No/ Probably No | | | | | | | | | | | Yes/ Probably Yes | | | | | | | | No Information | | | |
| Risk-of-bias judgment (low/high/some concerns) | | | | | | |  | | | | | | | | | | | | | | | | | | | | | | |
| Optional: What is the predicted direction bias due to selection of the reported results? | | | | | | |  | | | | | | | | | | | | | | | | | | | | | | |
| **6. Overall bias** | | | | | | | | | | | | | | | | | | | | | | | | | | | | | |
| Risk-of-bias judgment (low/high/some concerns) | | | | | | |  | | | | | | | | | | | | | | | | | | | | | | |
| Optional: What is the overall predicted direction of bias for this outcome? | | | | | | |  | | | | | | | | | | | | | | | | | | | | | | |
|  | | | | | | | | | | | | | | | | | | | | | | | | | | | | | |
| **B. The original Cochrane RoB tool (introduced in 2008 and edited on March 20, 2011)** | | | | | | | | | | | | | | | | | | | | | | | | | | | | | |
| Major Components | | | | Response options | | | | | | | | | | | | | | | | | | | | | | | | | |
| 1.Random sequence generation | | | | Lower risk of bias | | | | | | | | | | Higher risk of bias | | | | | | | | | Unclear risk of bias | | | | | | |
| 2. Allocation concealment | | | | Lower risk of bias | | | | | | | | | | Higher risk of bias | | | | | | | | | Unclear risk of bias | | | | | | |
| 3. Blinding of participants and personnel | | | | Lower risk of bias | | | | | | | | | | Higher risk of bias | | | | | | | | | Unclear risk of bias | | | | | | |
| 4. Blinding of outcome assessment | | | | Lower risk of bias | | | | | | | | | | Higher risk of bias | | | | | | | | | Unclear risk of bias | | | | | | |
| 5. Incomplete outcome data | | | | Lower risk of bias | | | | | | | | | | Higher risk of bias | | | | | | | | | Unclear risk of bias | | | | | | |
| 6. Selective reporting | | | | Lower risk of bias | | | | | | | | | | Higher risk of bias | | | | | | | | | Unclear risk of bias | | | | | | |
| 7. Other sources of bias | | | | Lower risk of bias | | | | | | | | | | Higher risk of bias | | | | | | | | | Unclear risk of bias | | | | | | |
|  | | | | | | | | | | | | | | | | | | | | | | | | | | | | | |
| **C. The Physiotherapy Evidence Database (PEDro) scale (last amended on June 21, 1999)**  **Website:** http://www.pedro.org.au/english/downloads/pedro-scale/ | | | | | | | | | | | | | | | | | | | | | | | | | | | | | |
| Major Components | | | | | | | | | | | | | | Response options | | | | | | | | | | | | | | | |
| 1. Eligibility criteria were specified | | | | | | | | | | | | | | Yes (1 point) | | | | | | | | | No (0 point) | | | | | | |
| 2. Subjects were randomly allocated to groups (in a crossover study, subjects were randomly allocated an order in which treatments were received) | | | | | | | | | | | | | | Yes (1 point) | | | | | | | | | No (0 point) | | | | | | |
| 3. Allocation was concealed | | | | | | | | | | | | | | Yes (1 point) | | | | | | | | | No (0 point) | | | | | | |
| 4. The groups were similar at baseline regarding the most important prognostic indicators | | | | | | | | | | | | | | Yes (1 point) | | | | | | | | | No (0 point) | | | | | | |
| 5. There was blinding of all subjects | | | | | | | | | | | | | | Yes (1 point) | | | | | | | | | No (0 point) | | | | | | |
| 6. There was blinding of all therapists who administered the therapy | | | | | | | | | | | | | | Yes (1 point) | | | | | | | | | No (0 point) | | | | | | |
| 7. There was blinding of all assessors who measured at least one key outcome | | | | | | | | | | | | | | Yes (1 point) | | | | | | | | | No (0 point) | | | | | | |
| 8. Measures of at least one key outcome were obtained from more than 85% of the subjects initially allocated to groups | | | | | | | | | | | | | | Yes (1 point) | | | | | | | | | No (0 point) | | | | | | |
| 9. All subjects for whom outcome measures were available received the treatment or control condition as allocated or, where this was not the case, data for at least one key outcome was analysed by “intention to treat” | | | | | | | | | | | | | | Yes (1 point) | | | | | | | | | No (0 point) | | | | | | |
| 10. The results of between-group statistical comparisons are reported for at least one key outcome | | | | | | | | | | | | | | Yes (1 point) | | | | | | | | | No (0 point) | | | | | | |
| 11. The study provides both point measures and measures of variability for at least one key outcome | | | | | | | | | | | | | | Yes (1 point) | | | | | | | | | No (0 point) | | | | | | |
|  | | | | | | | | | | | | | | | | | | | | | | | | | | | | | |
| **D. The Effective Practice and Organisation of Care (EPOC) RoB Tool for randomized trials (last introduced on August 22, 2017)**  **Website:** https://epoc.cochrane.org/resources/epoc-resources-review-authors | | | | | | | | | | | | | | | | | | | | | | | | | | | | | |
| Major Components | | | | Response options | | | | | | | | | | | | | | | | | | | | | | | | | |
| 1. Random sequence generation | | | | Lower risk | | | | | | | | | | Higher risk | | | | | | | | | Unclear risk | | | | | | |
| 2. Allocation concealment | | | | Lower risk | | | | | | | | | | Higher risk | | | | | | | | | Unclear risk | | | | | | |
| 3. Baseline outcome measurements similar^1,2^ | | | | Lower risk | | | | | | | | | | Higher risk | | | | | | | | | Unclear risk | | | | | | |
| 4. Baseline characteristics similar | | | | Lower risk | | | | | | | | | | Higher risk | | | | | | | | | Unclear risk | | | | | | |
| 5. Incomplete outcome data^1^ | | | | Lower risk | | | | | | | | | | Higher risk | | | | | | | | | Unclear risk | | | | | | |
| 6. Knowledge of the allocated interventions adequately prevented during the study ^1,3^ | | | | Lower risk | | | | | | | | | | Higher risk | | | | | | | | | Unclear risk | | | | | | |
| 7. Protection against contamination | | | | Lower risk | | | | | | | | | | Higher risk | | | | | | | | | Unclear risk | | | | | | |
| 8. Selective outcome reporting | | | | Lower risk | | | | | | | | | | Higher risk | | | | | | | | | Unclear risk | | | | | | |
| 9. Other risks of bias | | | | Lower risk | | | | | | | | | | Higher risk | | | | | | | | | Unclear risk | | | | | | |
| ^1^If some primary outcomes were imbalanced at baseline, assessed blindly or affected by missing data and others were not, each primary outcome can be scored separately;  ^2^If “Unclear risk” or “High risk”, but there is sufficient data in the paper to do an adjusted analysis (e.g. Baseline adjustment analysis or Intention to treat analysis) the criteria should be re scored as “Low risk”;  ^3^This refers to blinding of participants and personnel and blinding of outcome assessment. | | | | | | | | | | | | | | | | | | | | | | | | | | | | | |
|  | | | | | | | | | | | | | | | | | | | | | | | | | | | | | |
| **E. The Critical Appraisal Skills Programme (CASP) Checklist for RCT (last amended in 2018)**  **Website:** https://casp-uk.net/casp-tools-checklists/ | | | | | | | | | | | | | | | | | | | | | | | | | | | | | |
| Major Components | | | | Response options | | | | | | | | | | | | | | | | | | | | | | | | | |
| **Section A: Are the results of the trial valid?** | | | | | | | | | | | | | | | | | | | | | | | | | | | | | |
| 1. Did the trial address a clearly focused issue? | | | | Yes | | | | | | | | | | Can’t tell | | | | | | | | | No | | | | | | |
| 2. Was the assignment of patients to treatments randomised? | | | | Yes | | | | | | | | | | Can’t tell | | | | | | | | | No | | | | | | |
| 3. Were all of the patients who entered the trial properly accounted for at its conclusion? | | | | Yes | | | | | | | | | | Can’t tell | | | | | | | | | No | | | | | | |
| Is it worth continuing? | | | | | | | | | | | | | | | | | | | | | | | | | | | | | |
| 4. Were patients, health workers and study personnel ‘blind’ to treatment? | | | | Yes | | | | | | | | | | Can’t tell | | | | | | | | | No | | | | | | |
| 5. Were the groups similar at the start of the trial? | | | | Yes | | | | | | | | | | Can’t tell | | | | | | | | | No | | | | | | |
| 6. Aside from the experimental intervention, were the groups treated equally? | | | | Yes | | | | | | | | | | Can’t tell | | | | | | | | | No | | | | | | |
| **Section B: What are the results?** | | | | | | | | | | | | | | | | | | | | | | | | | | | | | |
| 7. How large was the treatment effect? | | | |  | | | | | | | | | | | | | | | | | | | | | | | | | |
| 8. How precise was the estimate of the treatment effect? | | | |  | | | | | | | | | | | | | | | | | | | | | | | | | |
| **Section C: Will the results help locally?** | | | | | | | | | | | | | | | | | | | | | | | | | | | | | |
| 9. Can the results be applied to the local population, or in your context? | | | | Yes | | | | | | | | | | Can’t tell | | | | | | | | | No | | | | | | |
| 10. Were all clinically important outcomes considered? | | | | Yes | | | | | | | | | | Can’t tell | | | | | | | | | No | | | | | | |
| 11. Are the benefits worth the harms and costs? | | | | Yes | | | | | | | | | | Can’t tell | | | | | | | | | No | | | | | | |
|  | | | | | | | | | | | | | | | | | | | | | | | | | | | | | |
| **F. The National Institutes of Health (NIH) quality assessment tool of controlled intervention study**  **Website:** https://www.nhlbi.nih.gov/health-topics/study-quality-assessment-tools | | | | | | | | | | | | | | | | | | | | | | | | | | | | | |
| Major Components | | | | | Response options | | | | | | | | | | | | | | | | | | | | | | | | |
| 1. Was the study described as randomized, a randomized trial, a randomized clinical trial, or an RCT? | | | | | Yes | | | | | No | | | Cannot Determine/ Not Applicable/ Not Reported | | | | | | | | | | | | | | | | |
| 2. Was the method of randomization adequate (i.e., use of randomly generated assignment)? | | | | | Yes | | | | | No | | | Cannot Determine/ Not Applicable/ Not Reported | | | | | | | | | | | | | | | | |
| 3. Was the treatment allocation concealed (so that assignments could not be predicted)? | | | | | Yes | | | | | No | | | Cannot Determine/ Not Applicable/ Not Reported | | | | | | | | | | | | | | | | |
| 4. Were study participants and providers blinded to treatment group assignment? | | | | | Yes | | | | | No | | | Cannot Determine/ Not Applicable/ Not Reported | | | | | | | | | | | | | | | | |
| 5. Were the people assessing the outcomes blinded to the participants' group assignments? | | | | | Yes | | | | | No | | | Cannot Determine/ Not Applicable/ Not Reported | | | | | | | | | | | | | | | | |
| 6. Were the groups similar at baseline on important characteristics that could affect outcomes (e.g., demographics, risk factors, co-morbid conditions)? | | | | | Yes | | | | | No | | | Cannot Determine/ Not Applicable/ Not Reported | | | | | | | | | | | | | | | | |
| 7. Was the overall drop-out rate from the study at endpoint 20% or lower of the number allocated to treatment? | | | | | Yes | | | | | No | | | Cannot Determine/ Not Applicable/ Not Reported | | | | | | | | | | | | | | | | |
| 8. Was the differential drop-out rate (between treatment groups) at endpoint 15 percentage points or lower? | | | | | Yes | | | | | No | | | Cannot Determine/ Not Applicable/ Not Reported | | | | | | | | | | | | | | | | |
| 9. Was there high adherence to the intervention protocols for each treatment group? | | | | | Yes | | | | | No | | | Cannot Determine/ Not Applicable/ Not Reported | | | | | | | | | | | | | | | | |
| 10. Were other interventions avoided or similar in the groups (e.g., similar background treatments)? | | | | | Yes | | | | | No | | | Cannot Determine/ Not Applicable/ Not Reported | | | | | | | | | | | | | | | | |
| 11. Were outcomes assessed using valid and reliable measures, implemented consistently across all study participants? | | | | | Yes | | | | | No | | | Cannot Determine/ Not Applicable/ Not Reported | | | | | | | | | | | | | | | | |
| 12. Did the authors report that the sample size was sufficiently large to be able to detect a difference in the main outcome between groups with at least 80% power? | | | | | Yes | | | | | No | | | Cannot Determine/ Not Applicable/ Not Reported | | | | | | | | | | | | | | | | |
| 13. Were outcomes reported or subgroups analyzed prespecified (i.e., identified before analyses were conducted)? | | | | | Yes | | | | | No | | | Cannot Determine/ Not Applicable/ Not Reported | | | | | | | | | | | | | | | | |
| 14. Were all randomized participants analyzed in the group to which they were originally assigned, i.e., did they use an intention-to-treat analysis? | | | | | Yes | | | | | No | | | Cannot Determine/ Not Applicable/ Not Reported | | | | | | | | | | | | | | | | |
| **Quality Rating** | | | | | Good | | | | | Fair | | | Poor | | | | | | | | | | | | | | | | |
| Additional Comments (If Poor, please state why): | | | | | | | | | | | | | | | | | | | | | | | | | | | | | |
|  | | | | | | | | | | | | | | | | | | | | | | | | | | | | | |
| **G. The Joanna Briggs Institute (JBI) Critical Appraisal Checklist for RCTs (last amended in 2017)**  **Website:** https://joannabriggs.org/critical_appraisal_tools  https://wiki.joannabriggs.org/display/MANUAL/Appendix+3.1%3A+JBI+Critical+appraisal+checklist+for+randomized+controlled+trials | | | | | | | | | | | | | | | | | | | | | | | | | | | | | |
| Major Components | | | | | | | | | Response options | | | | | | | | | | | | | | | | | | | | |
| 1. Was true randomization used for assignment of participants to treatment groups? | | | | | | | | | Yes | | | | | | No | | | | | | Unclear | | | | Not Applicable | | | | |
| 2. Was allocation to treatment groups concealed? | | | | | | | | | Yes | | | | | | No | | | | | | Unclear | | | | Not Applicable | | | | |
| 3. Were treatment groups similar at the baseline? | | | | | | | | | Yes | | | | | | No | | | | | | Unclear | | | | Not Applicable | | | | |
| 4. Were participants blind to treatment assignment? | | | | | | | | | Yes | | | | | | No | | | | | | Unclear | | | | Not Applicable | | | | |
| 5. Were those delivering treatment blind to treatment assignment? | | | | | | | | | Yes | | | | | | No | | | | | | Unclear | | | | Not Applicable | | | | |
| 6. Were outcomes assessors blind to treatment assignment? | | | | | | | | | Yes | | | | | | No | | | | | | Unclear | | | | Not Applicable | | | | |
| 7. Were treatment groups treated identically other than the intervention of interest? | | | | | | | | | Yes | | | | | | No | | | | | | Unclear | | | | Not Applicable | | | | |
| 8. Was follow up complete and if not, were differences between groups in terms of their follow up adequately described and analyzed? | | | | | | | | | Yes | | | | | | No | | | | | | Unclear | | | | Not Applicable | | | | |
| 9. Were participants analyzed in the groups to which they were randomized? | | | | | | | | | Yes | | | | | | No | | | | | | Unclear | | | | Not Applicable | | | | |
| 10. Were outcomes measured in the same way for treatment groups? | | | | | | | | | Yes | | | | | | No | | | | | | Unclear | | | | Not Applicable | | | | |
| 11. Were outcomes measured in a reliable way? | | | | | | | | | Yes | | | | | | No | | | | | | Unclear | | | | Not Applicable | | | | |
| 12. Was appropriate statistical analysis used? | | | | | | | | | Yes | | | | | | No | | | | | | Unclear | | | | Not Applicable | | | | |
| 13. Was the trial design appropriate, and any deviations from the standard RCT design (individual randomization, parallel groups) accounted for in the conduct and analysis of the trial? | | | | | | | | | Yes | | | | | | No | | | | | | Unclear | | | | Not Applicable | | | | |
| Overall appraisal: Include □ Exclude □ Seek further info □ | | | | | | | | | | | | | | | | | | | | | | | | | | | | | |
|  | | | | | | | | | | | | | | | | | | | | | | | | | | | | | |
| **H. The Scottish Intercollegiate Guidelines Network (SIGN) Methodology checklist: RCTs (last amended in 2014)**  **Website:** https://www.sign.ac.uk/checklists-and-notes.html | | | | | | | | | | | | | | | | | | | | | | | | | | | | | |
| Major Components | | | | | | | | Response options | | | | | | | | | | | | | | | | | | | | | |
| **SECTION 1: INTERNAL VALIDITY** | | | | | | | | | | | | | | | | | | | | | | | | | | | | | |
| 1.1. The study addresses an appropriate and clearly focused question | | | | | | | | Yes | | | | | | | No | | | | | | | Can’t say | | | | | | | |
| 1.2. The assignment of subjects to treatment groups is randomised | | | | | | | | Yes | | | | | | | No | | | | | | | Can’t say | | | | | | | |
| 1.3. An adequate concealment method is used | | | | | | | | Yes | | | | | | | No | | | | | | | Can’t say | | | | | | | |
| 1.4. The design keeps subjects and investigators ‘blind’ about treatment allocation | | | | | | | | Yes | | | | | | | No | | | | | | | Can’t say | | | | | | | |
| 1.5. The treatment and control groups are similar at the start of the trial | | | | | | | | Yes | | | | | | | No | | | | | | | Can’t say | | | | | | | |
| 1.6. The only difference between groups is the treatment under investigation | | | | | | | | Yes | | | | | | | No | | | | | | | Can’t say | | | | | | | |
| 1.7. All relevant outcomes are measured in a standard, valid and reliable way | | | | | | | | Yes | | | | | | | No | | | | | | | Can’t say | | | | | | | |
| 1.8. What percentage of the individuals or clusters recruited into each treatment arm of the study dropped out before the study was completed? | | | | | | | |  | | | | | | | | | | | | | | | | | | | | | |
| 1.9. All the subjects are analysed in the groups to which they were randomly allocated (often referred to as intention to treat analysis | | | | | | | | Yes | | | | | | | No | | | | | | | Can’t say | | | | | | | |
| 1.10. Where the study is carried out at more than one site, results are comparable for all sites | | | | | | | | Yes | | | | | | | No | | | | | | | Can’t say | | | | | | | |
| **SECTION 2: OVERALL ASSESSMENT OF THE STUDY** | | | | | | | | | | | | | | | | | | | | | | | | | | | | | |
| 2.1. How well was the study done to minimise bias?  Code as follows: | | | | | | | | High quality (++) | | | | | | | Acceptable (+) | | | | | | | Low quality (-) | | | | | Unacceptable - reject 0 | | |
| 2.2. Taking into account clinical considerations, your evaluation of the methodology used, and the statistical power of the study, are you certain that the overall effect is due to the study intervention? | | | | | | | |  | | | | | | | | | | | | | | | | | | | | | |
| 2.3. Are the results of this study directly applicable to the patient group targeted by this guideline? | | | | | | | |  | | | | | | | | | | | | | | | | | | | | | |
| 2.4. Notes. Summarise the authors’ conclusions. Add any comments on your own assessment of the study, and the extent to which it answers your question and mention any areas of uncertainty raised above. | | | | | | | | | | | | | | | | | | | | | | | | | | | | | |
|  | | | | | | | | | | | | | | | | | | | | | | | | | | | | | |
| **I. SYRCLE’s (SYstematic Review Centre for Laboratory animal Experimentation) RoB (Risk of Bias) tool (released in 2014)**  **Website:** https://www.radboudumc.nl/en/research/departments/health-evidence/systematic-review-center-for-laboratory-animal-experimentation | | | | | | | | | | | | | | | | | | | | | | | | | | | | | |
| Major Components | | | | | | | | | | | | | | | | | | | | | Response options | | | | | | | | |
| 1. Was the allocation sequence adequately generated and applied?   - Did the investigators describe a random component in the sequence generation process such as: Referring to a random number table; Using a computer random number generator | | | | | | | | | | | | | | | | | | | | | Yes | | | No | | | | Unclear | |
| 2. Were the groups similar at baseline or were they adjusted for confounders in the analysis?   - Was the distribution of relevant baseline characteristics balanced for the intervention and control groups? - If relevant, did the investigators adequately adjust for unequal distribution of some relevant baseline characteristics in the analysis? - Was the timing of disease induction adequate? | | | | | | | | | | | | | | | | | | | | | Yes | | | No | | | | Unclear | |
| 3. Was the allocation to the different groups adequately concealed during?   - Could the investigator allocating the animals to intervention or control group not foresee assignment due to one of the following or equivalent methods? Third-party coding of experimental and control group allocation Central randomization by a third party Sequentially numbered opaque, sealed envelopes | | | | | | | | | | | | | | | | | | | | | Yes | | | No | | | | Unclear | |
| 4. Were the animals randomly housed during the experiment?   - Did the authors randomly place the cages or animals within the animal room/facility? Animals were selected at random during outcome assessment (use signaling questions of entry 6). - Is it unlikely that the outcome or the outcome measurement was influenced by not randomly housing the animals? | | | | | | | | | | | | | | | | | | | | | Yes | | | No | | | | Unclear | |
| 5. Were the caregivers and/or investigators blinded from knowledge which intervention each animal received during the experiment?   - Was blinding of caregivers and investigators ensured, and was it unlikely that their blinding could have been broken? ID cards of individual animals, or cage/animal labels are coded and identical in appearance; Sequentially numbered drug containers are identical in appearance; The circumstances during the intervention are specified and similar in both groups (#).; Housing conditions of the animals during the experiment are randomized within the room (use criteria of entry 4). | | | | | | | | | | | | | | | | | | | | | Yes | | | No | | | | Unclear | |
| The relevance of the above-mentioned items depends on the experiment. Authors of the review need to judge for themselves which of the above-mentioned items could cause bias in the results when not similar. These should be assessed. | | | | | | | | | | | | | | | | | | | | | | | | | | | | | |
| 6. Were animals selected at random for outcome assessment?   - Did the investigators randomly pick an animal during outcome assessment, or did they use a random component in the sequence generation for outcome assessment? Referring to a random number table; Using a computer random number generator; Etc. | | | | | | | | | | | | | | | | | | | | | Yes | | | No | | | | Unclear | |
| 7. Was the outcome assessor blinded?   - Was blinding of the outcome assessor ensured, and was it unlikely that blinding could have been broken? Outcome assessment methods were the same in both groups; Animals were selected at random during outcome assessment (use signaling questions of entry 6). - Was the outcome assessor not blinded, but do review authors judge that the outcome is not likely to be influenced by lack of blinding? (e.g., mortality) | | | | | | | | | | | | | | | | | | | | | Yes | | | No | | | | Unclear | |
| 8. Were incomplete outcome data adequately addressed?   - Were all animals included in the analysis? - Were the reasons for missing outcome data unlikely to be related to true outcome? (e.g., technical failure) - Are missing outcome data balanced in numbers across intervention groups, with similar reasons for missing data across groups? - Are missing outcome data imputed using appropriate methods? | | | | | | | | | | | | | | | | | | | | | Yes | | | No | | | | Unclear | |
| 9. Are reports of the study free of selective outcome reporting?   - Was the study protocol available and were all of the study’s pre-specified primary and secondary outcomes reported in the current manuscript? - Was the study protocol not available, but was it clear that the published report included all expected outcomes (i.e. comparing methods and results section)? | | | | | | | | | | | | | | | | | | | | | Yes | | | No | | | | Unclear | |
| 10. Was the study apparently free of other problems that could result in high risk of bias?   - Was the study free of contamination (pooling drugs)? - Was the study free of inappropriate influence of funders? - Was the study free of unit of analysis errors? - Were design-specific risks of bias absent? - Were new animals added to the control and experimental groups to replace drop-outs from the original population? | | | | | | | | | | | | | | | | | | | | | Yes | | | No | | | | Unclear | |
|  | | | | | | | | | | | | | | | | | | | | | | | | | | | | | |
| **J. The Collaborative Approach to Meta-Analysis and Review of Animal Data from Experimental Studies (CAMARADES) tool (released in 2004)**  **Website:** http://www.camarades.info/ | | | | | | | | | | | | | | | | | | | | | | | | | | | | | |
| Major Components | | | | | | | | Response options | | | | | | | | | | | | | | | | | | | | | |
| 1. Sample size calculation | | | | | | | | Yes | | | | | | | | | | | No | | | | | | Unclear | | | | |
| 2. Random allocation to treatment or control | | | | | | | | Yes | | | | | | | | | | | No | | | | | | Unclear | | | | |
| 3. Blinded induction of ischemia* | | | | | | | | Yes | | | | | | | | | | | No | | | | | | Unclear | | | | |
| 4. Blinded assessment of outcome | | | | | | | | Yes | | | | | | | | | | | No | | | | | | Unclear | | | | |
| 5. Appropriate animal model | | | | | | | | Yes | | | | | | | | | | | No | | | | | | Unclear | | | | |
| 6. Use of anesthetic without significant intrinsic neuroprotective activity* | | | | | | | | Yes | | | | | | | | | | | No | | | | | | Unclear | | | | |
| 7. Statement of control of temperature* | | | | | | | | Yes | | | | | | | | | | | No | | | | | | Unclear | | | | |
| 8. Compliance with animal welfare regulations | | | | | | | | Yes | | | | | | | | | | | No | | | | | | Unclear | | | | |
| 9. Peer-reviewed publication | | | | | | | | Yes | | | | | | | | | | | No | | | | | | Unclear | | | | |
| 10. Statement of potential conflict of interests | | | | | | | | Yes | | | | | | | | | | | No | | | | | | Unclear | | | | |
| *, can be modified by user when using in another animal models. | | | | | | | | | | | | | | | | | | | | | | | | | | | | | |
|  | | | | | | | | | | | | | | | | | | | | | | | | | | | | | |
| **K. The Risk Of Bias In Non-randomised Studies - of Interventions (ROBINS-I) tool (last edited in October, 2016)**  **Website:** https://www.riskofbias.info/ | | | | | | | | | | | | | | | | | | | | | | | | | | | | | |
| Major Components | | Response options | | | | | | | | | | | | | | | | | | | | | | | | | | | |
| **Part 1: Bias due to confounding** | | | | | | | | | | | | | | | | | | | | | | | | | | | | | |
| 1.1 Is there potential for confounding of the effect of intervention in this study?  If No/ Probably No to 1.1: the study can be considered to be at low risk of bias due to confounding and no further signalling questions need be considered  If Yes/ Probably Yes to 1.1: determine whether there is a need to assess time-varying confounding: | | Yes/ Probably Yes | | | | | | | | | No/ Probably No | | | | | | | | |  | | | | | | |  | | |
| 1.2. Was the analysis based on splitting participants’ follow up time according to intervention received?  If No/ Probably No, answer questions relating to baseline confounding (1.4 to 1.6)  If Yes/ Probably Yes, go to question 1.3. | | Yes/ Probably Yes | | | | | | | | | No/ Probably No | | | | | | | | | No Information | | | | | | | Not Applicable | | |
| 1.3. Were intervention discontinuations or switches likely to be related to factors that are prognostic for the outcome?  If No/ Probably No, answer questions relating to baseline confounding (1.4 to 1.6)  If Yes/ Probably Yes, answer questions relating to both baseline and time-varying confounding (1.7 and 1.8) | | Yes/ Probably Yes | | | | | | | | | No/ Probably No | | | | | | | | | No Information | | | | | | | Not Applicable | | |
| Questions relating to baseline confounding only (1.4 to 1.6) | | | | | | | | | | | | | | | | | | | | | | | | | | | | | |
| 1.4. Did the authors use an appropriate analysis method that controlled for all the important confounding domains? | | Yes/ Probably Yes | | | | | | | | | No/ Probably No | | | | | | | | | No Information | | | | | | | Not Applicable | | |
| 1.5. If Yes/ Probably Yes to 1.4: Were confounding domains that were controlled for measured validly and reliably by the variables available in this study? | | Yes/ Probably Yes | | | | | | | | | No/ Probably No | | | | | | | | | No Information | | | | | | | Not Applicable | | |
| 1.6. Did the authors control for any post-intervention variables that could have been affected by the intervention? | | Yes/ Probably Yes | | | | | | | | | No/ Probably No | | | | | | | | | No Information | | | | | | | Not Applicable | | |
| Questions relating to baseline and time-varying confounding (1.7to 1.8) | | | | | | | | | | | | | | | | | | | | | | | | | | | | | |
| 1.7. Did the authors use an appropriate analysis method that controlled for all the important confounding domains and for time-varying confounding? | | Yes/ Probably Yes | | | | | | | | | No/ Probably No | | | | | | | | | No Information | | | | | | | Not Applicable | | |
| 1.8. If Yes/ Probably Yes to 1.7: Were confounding domains that were controlled for measured validly and reliably by the variables available in this study? | | Yes/ Probably Yes | | | | | | | | | No/ Probably No | | | | | | | | | No Information | | | | | | | Not Applicable | | |
| **Risk of bias judgement:** | | Low risk of bias/ Moderate risk of bias/ Serious risk of bias/ Critical risk of bias/ No information | | | | | | | | | | | | | | | | | | | | | | | | | | | |
| Optional: What is the predicted direction of bias due to confounding? | | Favours experimental/ Favours comparator/ Unpredictable | | | | | | | | | | | | | | | | | | | | | | | | | | | |
| **Part 2: Bias in selection of participants into the study** | | | | | | | | | | | | | | | | | | | | | | | | | | | | | |
| 2.1. Was selection of participants into the study (or into the analysis) based on participant characteristics observed after the start of intervention?  If No/ Probably No to 2.1: go to 2.4 | | Yes/ Probably Yes | | | | | | | | | No/ Probably No | | | | | | | | | No Information | | | | | | |  | | |
| 2.2. If Yes/ Probably Yes to 2.1: Were the post-intervention variables that influenced selection likely to be associated with intervention? | | Yes/ Probably Yes | | | | | | | | | No/ Probably No | | | | | | | | | No Information | | | | | | | Not Applicable | | |
| 2.3 If Yes/ Probably Yes to 2.2: Were the post-intervention variables that influenced selection likely to be influenced by the outcome or a cause of the outcome? | | Yes/ Probably Yes | | | | | | | | | No/ Probably No | | | | | | | | | No Information | | | | | | | Not Applicable | | |
| 2.4. Do start of follow-up and start of intervention coincide for most participants? | | Yes/ Probably Yes | | | | | | | | | No/ Probably No | | | | | | | | | No Information | | | | | | |  | | |
| 2.5. If Yes/ Probably Yes to 2.2 and 2.3, or No/ Probably No to 2.4: Were adjustment techniques used that are likely to correct for the presence of selection biases? | | Yes/ Probably Yes | | | | | | | | | No/ Probably No | | | | | | | | | No Information | | | | | | | Not Applicable | | |
| **Risk of bias judgement:** | | Low risk of bias/ Moderate risk of bias/ Serious risk of bias/ Critical risk of bias/ No information | | | | | | | | | | | | | | | | | | | | | | | | | | | |
| Optional: What is the predicted direction of bias due to selection of participants into the study? | | Favours experimental/ Favours comparator/ Towards null/ Away from null/ Unpredictable | | | | | | | | | | | | | | | | | | | | | | | | | | | |
| **Part 3: Bias in classification of interventions** | |  | | | | | | | | |  | | | | | | | | |  | | | | | | |  | | |
| 3.1 Were intervention groups clearly defined? | | Yes/ Probably Yes | | | | | | | | | No/ Probably No | | | | | | | | | No Information | | | | | | |  | | |
| 3.2 Was the information used to define intervention groups recorded at the start of the intervention? | | Yes/ Probably Yes | | | | | | | | | No/ Probably No | | | | | | | | | No Information | | | | | | |  | | |
| 3.3 Could classification of intervention status have been affected by knowledge of the outcome or risk of the outcome? | | Yes/ Probably Yes | | | | | | | | | No/ Probably No | | | | | | | | | No Information | | | | | | |  | | |
| **Risk of bias judgement:** | | Low risk of bias/ Moderate risk of bias/ Serious risk of bias/ Critical risk of bias/ No information | | | | | | | | | | | | | | | | | | | | | | | | | | | |
| Optional: What is the predicted direction of bias due to measurement of outcomes or interventions? | | Favours experimental/ Favours comparator/ Towards null/ Away from null/ Unpredictable | | | | | | | | | | | | | | | | | | | | | | | | | | | |
| **Part 4: Bias due to deviations from intended interventions** | | | | | | | | | | | | | | | | | | | | | | | | | | | | | |
| If your aim for this study is to assess the effect of assignment to intervention, answer questions 4.1 and 4.2 | | | | | | | | | | | | | | | | | | | | | | | | | | | | | |
| 4.1. Were there deviations from the intended intervention beyond what would be expected in usual practice? | | Yes/ Probably Yes | | | | | | | | | No/ Probably No | | | | | | | | | No Information | | | | | | |  | | |
| 4.2. If Yes/ Probably Yes to 4.1: Were these deviations from intended intervention unbalanced between groups and likely to have affected the outcome? | | Yes/ Probably Yes | | | | | | | | | No/ Probably No | | | | | | | | | No Information | | | | | | | Not Applicable | | |
| If your aim for this study is to assess the effect of starting and adhering to intervention, answer questions 4.3 to 4.6 | | | | | | | | | | | | | | | | | | | | | | | | | | | | | |
| 4.3. Were important co-interventions balanced across intervention groups? | | Yes/ Probably Yes | | | | | | | | | No/ Probably No | | | | | | | | | No Information | | | | | | |  | | |
| 4.4. Was the intervention implemented successfully for most participants? | | Yes/ Probably Yes | | | | | | | | | No/ Probably No | | | | | | | | | No Information | | | | | | |  | | |
| 4.5. Did study participants adhere to the assigned intervention regimen? | | Yes/ Probably Yes | | | | | | | | | No/ Probably No | | | | | | | | | No Information | | | | | | |  | | |
| 4.6. If No/ Probably No to 4.3, 4.4 or 4.5: Was an appropriate analysis used to estimate the effect of starting and adhering to the intervention? | | Yes/ Probably Yes | | | | | | | | | No/ Probably No | | | | | | | | | No Information | | | | | | | Not Applicable | | |
| **Risk of bias judgement:** | | Low risk of bias/ Moderate risk of bias/ Serious risk of bias/ Critical risk of bias/ No information | | | | | | | | | | | | | | | | | | | | | | | | | | | |
| Optional: What is the predicted direction of bias due to deviations from the intended interventions? | | Favours experimental/ Favours comparator/ Towards null/ Away from null/ Unpredictable | | | | | | | | | | | | | | | | | | | | | | | | | | | |
| **Part 5: Bias due to missing data** | | | | | | | | | | | | | | | | | | | | | | | | | | | | | |
| 5.1 Were outcome data available for all, or nearly all, participants? | | Yes/ Probably Yes | | | | | | | | | No/ Probably No | | | | | | | | | No Information | | | | | | |  | | |
| 5.2 Were participants excluded due to missing data on intervention status? | | Yes/ Probably Yes | | | | | | | | | No/ Probably No | | | | | | | | | No Information | | | | | | |  | | |
| 5.3 Were participants excluded due to missing data on other variables needed for the analysis? | | Yes/ Probably Yes | | | | | | | | | No/ Probably No | | | | | | | | | No Information | | | | | | |  | | |
| 5.4 If No/ Probably No to 5.1, or Yes/ Probably Yes to 5.2 or 5.3: Are the proportion of participants and reasons for missing data similar across interventions? | | Yes/ Probably Yes | | | | | | | | | No/ Probably No | | | | | | | | | No Information | | | | | | | Not Applicable | | |
| 5.5 If PN/N to 5.1, or Y/PY to 5.2 or 5.3: Is there evidence that results were robust to the presence of missing data? | | Yes/ Probably Yes | | | | | | | | | No/ Probably No | | | | | | | | | No Information | | | | | | | Not Applicable | | |
| **Risk of bias judgement:** | | Low risk of bias/ Moderate risk of bias/ Serious risk of bias/ Critical risk of bias/ No information | | | | | | | | | | | | | | | | | | | | | | | | | | | |
| Optional: What is the predicted direction of bias due to missing data? | | Favours experimental/ Favours comparator/ Towards null/ Away from null/ Unpredictable | | | | | | | | | | | | | | | | | | | | | | | | | | | |
| **Part 6: Bias in measurement of outcomes** | | | | | | | | | | | | | | | | | | | | | | | | | | | | | |
| 6.1 Could the outcome measure have been influenced by knowledge of the intervention received? | | Yes/ Probably Yes | | | | | | | | | No/ Probably No | | | | | | | | | No Information | | | | | | |  | | |
| 6.2 Were outcome assessors aware of the intervention received by study participants? | | Yes/ Probably Yes | | | | | | | | | No/ Probably No | | | | | | | | | No Information | | | | | | |  | | |
| 6.3 Were the methods of outcome assessment comparable across intervention groups? | | Yes/ Probably Yes | | | | | | | | | No/ Probably No | | | | | | | | | No Information | | | | | | |  | | |
| 6.4 Were any systematic errors in measurement of the outcome related to intervention received? | | Yes/ Probably Yes | | | | | | | | | No/ Probably No | | | | | | | | | No Information | | | | | | |  | | |
| **Risk of bias judgement:** | | Low risk of bias/ Moderate risk of bias/ Serious risk of bias/ Critical risk of bias/ No information | | | | | | | | | | | | | | | | | | | | | | | | | | | |
| Optional: What is the predicted direction of bias due to measurement of outcomes? | | Favours experimental/ Favours comparator/ Towards null/ Away from null/ Unpredictable | | | | | | | | | | | | | | | | | | | | | | | | | | | |
| **Part 7: Bias in selection of the reported result** | | | | | | | | | | | | | | | | | | | | | | | | | | | | | |
| Is the reported effect estimate likely to be selected, on the basis of the results, from... | |  | | | | | | | | |  | | | | | | | | |  | | | | | | |  | | |
| 7.1. ... multiple outcome measurements within the outcome domain? | | Yes/ Probably Yes | | | | | | | | | No/ Probably No | | | | | | | | | No Information | | | | | | |  | | |
| 7.2 ... multiple analyses of the intervention-outcome relationship? | | Yes/ Probably Yes | | | | | | | | | No/ Probably No | | | | | | | | | No Information | | | | | | |  | | |
| 7.3 ... different subgroups? | | Yes/ Probably Yes | | | | | | | | | No/ Probably No | | | | | | | | | No Information | | | | | | |  | | |
| **Risk of bias judgement:** | | Low risk of bias/ Moderate risk of bias/ Serious risk of bias/ Critical risk of bias/ No information | | | | | | | | | | | | | | | | | | | | | | | | | | | |
| Optional: What is the predicted direction of bias due to selection of the reported result? | | Favours experimental/ Favours comparator/ Towards null/ Away from null/ Unpredictable | | | | | | | | | | | | | | | | | | | | | | | | | | | |
| **Overall bias** | | | | | | | | | | | | | | | | | | | | | | | | | | | | | |
| **Risk of bias judgement:** | | Low risk of bias/ Moderate risk of bias/ Serious risk of bias/ Critical risk of bias/ No information | | | | | | | | | | | | | | | | | | | | | | | | | | | |
| Optional: What is the overall predicted direction of bias for this outcome? | | Favours experimental/ Favours comparator/ Towards null/ Away from null/ Unpredictable | | | | | | | | | | | | | | | | | | | | | | | | | | | |
|  | | | | | | | | | | | | | | | | | | | | | | | | | | | | | |
| **L. The Joanna Briggs Institute (JBI) Critical Appraisal Checklist for Quasi-Experimental Studies (non-randomized experimental studies)** **(last amended in 2017)**  **Website:** https://joannabriggs.org/critical_appraisal_tools  https://wiki.joannabriggs.org/pages/viewpage.action?pageId=9273720 | | | | | | | | | | | | | | | | | | | | | | | | | | | | | |
| Major Components | | Response options | | | | | | | | | | | | | | | | | | | | | | | | | | | |
| 1. Is it clear in the study what is the ‘cause’ and what is the ‘effect’ (i.e. there is no confusion about which variable comes first)? | | Yes | | | | | | | | | No | | | | | | | | | Unclear | | | | | | | Not Applicable | | |
| 2. Were the participants included in any comparisons similar? | | Yes | | | | | | | | | No | | | | | | | | | Unclear | | | | | | | Not Applicable | | |
| 3. Were the participants included in any comparisons receiving similar treatment/care, other than the exposure or intervention of interest? | | Yes | | | | | | | | | No | | | | | | | | | Unclear | | | | | | | Not Applicable | | |
| 4. Was there a control group? | | Yes | | | | | | | | | No | | | | | | | | | Unclear | | | | | | | Not Applicable | | |
| 5. Were there multiple measurements of the outcome both pre and post the intervention/exposure? | | Yes | | | | | | | | | No | | | | | | | | | Unclear | | | | | | | Not Applicable | | |
| 6. Was follow up complete and if not, were differences between groups in terms of their follow up adequately described and analyzed? | | Yes | | | | | | | | | No | | | | | | | | | Unclear | | | | | | | Not Applicable | | |
| 7. Were the outcomes of participants included in any comparisons measured in the same way? | | Yes | | | | | | | | | No | | | | | | | | | Unclear | | | | | | | Not Applicable | | |
| 8. Were outcomes measured in a reliable way? | | Yes | | | | | | | | | No | | | | | | | | | Unclear | | | | | | | Not Applicable | | |
| 9. Was appropriate statistical analysis used? | | Yes | | | | | | | | | No | | | | | | | | | Unclear | | | | | | | Not Applicable | | |
| Overall appraisal: Include □ Exclude □ Seek further info □ | | | | | | | | | | | | | | | | | | | | | | | | | | | | | |
|  | | | | | | | | | | | | | | | | | | | | | | | | | | | | | |
| **M. The Methodological item for non-randomized studies (MINORS) tool^1^**  ^1^The first eight apply to both non-comparative and comparative studies, while the remaining four relate only to studies with two or more groups. The global ideal score being 16 for non-comparative studies and 24 for comparative studies. | | | | | | | | | | | | | | | | | | | | | | | | | | | | | |
| Major Components | Response options | | | | | | | | | | | | | | | | | | | | | | | | | | | | |
| 1. A clearly stated aim | Not reported (0 point) | | Reported but inadequate (1 point) | | | | | | | | | | | | | | | | | Reported and adequate (2 point) | | | | | | | | | |
| 2. Inclusion of consecutive patients | Not reported (0 point) | | Reported but inadequate (1 point) | | | | | | | | | | | | | | | | | Reported and adequate (2 point) | | | | | | | | | |
| 3. Prospective collection of data | Not reported (0 point) | | Reported but inadequate (1 point) | | | | | | | | | | | | | | | | | Reported and adequate (2 point) | | | | | | | | | |
| 4. Endpoints appropriate to the aim of the study | Not reported (0 point) | | Reported but inadequate (1 point) | | | | | | | | | | | | | | | | | Reported and adequate (2 point) | | | | | | | | | |
| 5. Unbiased assessment of the study endpoint | Not reported (0 point) | | Reported but inadequate (1 point) | | | | | | | | | | | | | | | | | Reported and adequate (2 point) | | | | | | | | | |
| 6. Follow-up period appropriate to the aim of the study | Not reported (0 point) | | Reported but inadequate (1 point) | | | | | | | | | | | | | | | | | Reported and adequate (2 point) | | | | | | | | | |
| 7. Loss to follow up less than 5% | Not reported (0 point) | | Reported but inadequate (1 point) | | | | | | | | | | | | | | | | | Reported and adequate (2 point) | | | | | | | | | |
| 8. Prospective calculation of the study size | Not reported (0 point) | | Reported but inadequate (1 point) | | | | | | | | | | | | | | | | | Reported and adequate (2 point) | | | | | | | | | |
| 9. An adequate control group | Not reported (0 point) | | Reported but inadequate (1 point) | | | | | | | | | | | | | | | | | Reported and adequate (2 point) | | | | | | | | | |
| 10. Contemporary groups | Not reported (0 point) | | Reported but inadequate (1 point) | | | | | | | | | | | | | | | | | Reported and adequate (2 point) | | | | | | | | | |
| 11. Baseline equivalence of groups | Not reported (0 point) | | Reported but inadequate (1 point) | | | | | | | | | | | | | | | | | Reported and adequate (2 point) | | | | | | | | | |
| 12. Adequate statistical analyses | Not reported (0 point) | | Reported but inadequate (1 point) | | | | | | | | | | | | | | | | | Reported and adequate (2 point) | | | | | | | | | |
|  | | | | | | | | | | | | | | | | | | | | | | | | | | | | | |
| **N. The Canada Institute of Health Economics (IHE) Quality Appraisal Tool for Case Series (Interventional; last amended in 2012)**  **Website:** https://www.ihe.ca/research-programs/rmd/cssqac/cssqac-about | | | | | | | | | | | | | | | | | | | | | | | | | | | | | |
| Major Components | | | | | | | | | | | Response options | | | | | | | | | | | | | | | | | | |
| 1. Is the hypothesis/aim/objective of the study clearly stated? | | | | | | | | | | | Yes | | | | | | Unclear | | | | | | | | | | | | No |
| 2. Are the characteristics of the participants included in the study described? | | | | | | | | | | | Yes | | | | | | Partially Reported | | | | | | | | | | | | No |
| 3. Were the cases collected in more than one centre? | | | | | | | | | | | Yes | | | | | | Unclear | | | | | | | | | | | | No |
| 4. Are the eligibility criteria (i.e. inclusion and exclusion criteria) for entry into the study clearly stated? | | | | | | | | | | | Yes | | | | | | Partially Reported | | | | | | | | | | | | No |
| 5. Were participants recruited consecutively? | | | | | | | | | | | Yes | | | | | | Unclear | | | | | | | | | | | | No |
| 6. Did participants enter the study at a similar point in the disease? | | | | | | | | | | | Yes | | | | | | Unclear | | | | | | | | | | | | No |
| 7. Was the intervention of interest clearly described? | | | | | | | | | | | Yes | | | | | | Partially Reported | | | | | | | | | | | | No |
| 8. Were additional interventions (co-interventions) reported in the study? | | | | | | | | | | | Yes | | | | | | Unclear | | | | | | | | | | | | No |
| 9. Are the outcome measures established a priori? | | | | | | | | | | | Yes | | | | | | Partially Reported | | | | | | | | | | | | No |
| 10. Were the relevant outcomes measured with appropriate objective and/or subjective methods? | | | | | | | | | | | Yes | | | | | | Unclear | | | | | | | | | | | | No |
| 11. Were the relevant outcomes measured before and after the intervention? | | | | | | | | | | | Yes | | | | | | Unclear | | | | | | | | | | | | No |
| 12. Were the statistical tests used to assess the relevant outcomes appropriate? | | | | | | | | | | | Yes | | | | | | Unclear | | | | | | | | | | | | No |
| 13. Was the length of follow-up reported? | | | | | | | | | | | Yes | | | | | | Unclear | | | | | | | | | | | | No |
| 14. Was the loss to follow-up reported? | | | | | | | | | | | Yes | | | | | | Unclear | | | | | | | | | | | | No |
| 15. Does the study provide estimates of the random variability in the data analysis of relevant outcomes? | | | | | | | | | | | Yes | | | | | | Unclear/ Partially Reported | | | | | | | | | | | | No |
| 16. Are the adverse events related with the intervention reported? | | | | | | | | | | | Yes | | | | | | Unclear | | | | | | | | | | | | No |
| 17. Are the conclusions of the study supported by results? | | | | | | | | | | | Yes | | | | | | Partially Reported | | | | | | | | | | | | No |
| 18. Are both competing interests and sources of support for the study reported? | | | | | | | | | | | Yes | | | | | | Partially Reported | | | | | | | | | | | | No |
|  | | | | | | | | | | | | | | | | | | | | | | | | | | | | | |
| **O. The National Institutes of Health (NIH) quality assessment tool for case-series study (Interventional)**  **Website:** https://www.nhlbi.nih.gov/health-topics/study-quality-assessment-tools | | | | | | | | | | | | | | | | | | | | | | | | | | | | | |
| Major Components | | Response options | | | | | | | | | | | | | | | | | | | | | | | | | | | |
| 1. Was the study question or objective clearly stated? | | Yes | | | | | | No | | | | Cannot Determine/ Not Applicable/ Not Reported | | | | | | | | | | | | | | | | | |
| 2. Was the study population clearly and fully described, including a case definition? | | Yes | | | | | | No | | | | Cannot Determine/ Not Applicable/ Not Reported | | | | | | | | | | | | | | | | | |
| 3. Were the cases consecutive? | | Yes | | | | | | No | | | | Cannot Determine/ Not Applicable/ Not Reported | | | | | | | | | | | | | | | | | |
| 4. Were the subjects comparable? | | Yes | | | | | | No | | | | Cannot Determine/ Not Applicable/ Not Reported | | | | | | | | | | | | | | | | | |
| 5. Was the intervention clearly described? | | Yes | | | | | | No | | | | Cannot Determine/ Not Applicable/ Not Reported | | | | | | | | | | | | | | | | | |
| 6. Were the outcome measures clearly defined, valid, reliable, and implemented consistently across all study participants? | | Yes | | | | | | No | | | | Cannot Determine/ Not Applicable/ Not Reported | | | | | | | | | | | | | | | | | |
| 7. Was the length of follow-up adequate? | | Yes | | | | | | No | | | | Cannot Determine/ Not Applicable/ Not Reported | | | | | | | | | | | | | | | | | |
| 8. Were the statistical methods well-described? | | Yes | | | | | | No | | | | Cannot Determine/ Not Applicable/ Not Reported | | | | | | | | | | | | | | | | | |
| 9. Were the results well-described? | | Yes | | | | | | No | | | | Cannot Determine/ Not Applicable/ Not Reported | | | | | | | | | | | | | | | | | |
| **Quality Rating** | | Good | | | | | | Fair | | | | Poor | | | | | | | | | | | | | | | | | |
| Additional Comments (If Poor, please state why): | | | | | | | | | | | | | | | | | | | | | | | | | | | | | |
|  | | | | | | | | | | | | | | | | | | | | | | | | | | | | | |
| **P. The Effective Practice and Organisation of Care (EPOC) RoB Tool (last introduced on August 22, 2017)**  **Website:** https://epoc.cochrane.org/resources/epoc-resources-review-authors | | | | | | | | | | | | | | | | | | | | | | | | | | | | | |
| Major Components | | | | | | Response options | | | | | | | | | | | | | | | | | | | | | | | |
| 1. Intervention independent of other changes | | | | | | Low risk | | | | | | | | | | High risk | | | | | | | Unclear risk | | | | | | |
| 2. Shape of the intervention effect pre-specified | | | | | | Low risk | | | | | | | | | | High risk | | | | | | | Unclear risk | | | | | | |
| 3. Intervention unlikely to affect data collection | | | | | | Low risk | | | | | | | | | | High risk | | | | | | | Unclear risk | | | | | | |
| 4. Knowledge of the allocated interventions adequately prevented during the study^1,2^ | | | | | | Low risk | | | | | | | | | | High risk | | | | | | | Unclear risk | | | | | | |
| 5. Incomplete outcome data adequately^2^ | | | | | | Low risk | | | | | | | | | | High risk | | | | | | | Unclear risk | | | | | | |
| 6. Selective outcome reporting | | | | | | Low risk | | | | | | | | | | High risk | | | | | | | Unclear risk | | | | | | |
| 7. Other risks of bias | | | | | | Low risk | | | | | | | | | | High risk | | | | | | | Unclear risk | | | | | | |
| ^1^ This refers to blinding of participants and personnel and blinding of outcome assessment;  ^2^ If some primary outcomes were assessed blindly or affected by missing data and others were not, each primary outcome can be scored separately. | | | | | | | | | | | | | | | | | | | | | | | | | | | | | |
|  | | | | | | | | | | | | | | | | | | | | | | | | | | | | | |
| **Q. The National Institutes of Health (NIH) quality assessment tool for before-after (Pre-Post) study with no control group**  **Website:** https://www.nhlbi.nih.gov/health-topics/study-quality-assessment-tools | | | | | | | | | | | | | | | | | | | | | | | | | | | | | |
| Major Components | | | | | Response options | | | | | | | | | | | | | | | | | | | | | | | | |
| 1. Was the study question or objective clearly stated? | | | | | Yes | | | | | No | | | Cannot Determine/ Not Applicable/ Not Reported | | | | | | | | | | | | | | | | |
| 2. Were eligibility/selection criteria for the study population prespecified and clearly described? | | | | | Yes | | | | | No | | | Cannot Determine/ Not Applicable/ Not Reported | | | | | | | | | | | | | | | | |
| 3. Were the participants in the study representative of those who would be eligible for the test/service/intervention in the general or clinical population of interest? | | | | | Yes | | | | | No | | | Cannot Determine/ Not Applicable/ Not Reported | | | | | | | | | | | | | | | | |
| 4. Were all eligible participants that met the prespecified entry criteria enrolled? | | | | | Yes | | | | | No | | | Cannot Determine/ Not Applicable/ Not Reported | | | | | | | | | | | | | | | | |
| 5. Was the sample size sufficiently large to provide confidence in the findings? | | | | | Yes | | | | | No | | | Cannot Determine/ Not Applicable/ Not Reported | | | | | | | | | | | | | | | | |
| 6. Was the test/service/intervention clearly described and delivered consistently across the study population? | | | | | Yes | | | | | No | | | Cannot Determine/ Not Applicable/ Not Reported | | | | | | | | | | | | | | | | |
| 7. Were the outcome measures prespecified, clearly defined, valid, reliable, and assessed consistently across all study participants? | | | | | Yes | | | | | No | | | Cannot Determine/ Not Applicable/ Not Reported | | | | | | | | | | | | | | | | |
| 8. Were the people assessing the outcomes blinded to the participants' exposures/interventions? | | | | | Yes | | | | | No | | | Cannot Determine/ Not Applicable/ Not Reported | | | | | | | | | | | | | | | | |
| 9. Was the loss to follow-up after baseline 20% or less? Were those lost to follow-up accounted for in the analysis? | | | | | Yes | | | | | No | | | Cannot Determine/ Not Applicable/ Not Reported | | | | | | | | | | | | | | | | |
| 10. Did the statistical methods examine changes in outcome measures from before to after the intervention? Were statistical tests done that provided p values for the pre-to-post changes? | | | | | Yes | | | | | No | | | Cannot Determine/ Not Applicable/ Not Reported | | | | | | | | | | | | | | | | |
| 11. Were outcome measures of interest taken multiple times before the intervention and multiple times after the intervention (i.e., did they use an interrupted time-series design)? | | | | | Yes | | | | | No | | | Cannot Determine/ Not Applicable/ Not Reported | | | | | | | | | | | | | | | | |
| 12. If the intervention was conducted at a group level (e.g., a whole hospital, a community, etc.) did the statistical analysis take into account the use of individual-level data to determine effects at the group level? | | | | | Yes | | | | | No | | | Cannot Determine/ Not Applicable/ Not Reported | | | | | | | | | | | | | | | | |
| **Quality Rating** | | | | | Good | | | | | Fair | | | Poor | | | | | | | | | | | | | | | | |
| Additional Comments (If Poor, please state why): | | | | | | | | | | | | | | | | | | | | | | | | | | | | | |
