# Supplementary material for: Methodological quality (risk of bias) assessment tools for primary and secondary medical studies: what are they and which is better?
Source: Mil Med Res. 2020 Feb 29;7:7. doi: 10.1186/s40779-020-00238-8 (PMC7049186; doi:10.1186/s40779-020-00238-8)
Supplement: Supplementary file 2 — Additional file 2: Table S2. Major components of the tools for assessing observational studies and diagnostic study [file 40779_2020_238_MOESM2_ESM.docx]

**Table S2. Major components of the tools for assessing observational studies and diagnostic study**

| **A. The Critical Appraisal Skills Programme (CASP) checklist for cohort study (last amended in 2018)**  **Website:** http://www.casp-uk.net/#!casp-tools-checklists/c18f8 | | | | | | | | | | | | | | | | | | | | | | | | | | | | | | | | | | | | | | | | | | | | | | | | | |
| --- | --- | --- | --- | --- | --- | --- | --- | --- | --- | --- | --- | --- | --- | --- | --- | --- | --- | --- | --- | --- | --- | --- | --- | --- | --- | --- | --- | --- | --- | --- | --- | --- | --- | --- | --- | --- | --- | --- | --- | --- | --- | --- | --- | --- | --- | --- | --- | --- | --- |
| Major Components | | | | | | | | Response options | | | | | | | | | | | | | | | | | | | | | | | | | | | | | | | | | | | | | | | | | |
| **Section A: Are the results of the study valid?** | | | | | | | | | | | | | | | | | | | | | | | | | | | | | | | | | | | | | | | | | | | | | | | | | |
| 1. Did the study address a clearly focused issue? | | | | | | | | Yes | | | | | | | | | | | | | | | | | | | | No | | | | | | | | | | | | | | | | | | Can’t Tell | | | |
| 2. Was the cohort recruited in an acceptable way? | | | | | | | | Yes | | | | | | | | | | | | | | | | | | | | No | | | | | | | | | | | | | | | | | | Can’t Tell | | | |
| Is it worth continuing? | | | | | | | |  | | | | | | | | | | | | | | | | | | | |  | | | | | | | | | | | | | | | | | |  | | | |
| 3. Was the exposure accurately measured to minimise bias? | | | | | | | | Yes | | | | | | | | | | | | | | | | | | | | No | | | | | | | | | | | | | | | | | | Can’t Tell | | | |
| 4. Was the outcome accurately measured to minimise bias? | | | | | | | | Yes | | | | | | | | | | | | | | | | | | | | No | | | | | | | | | | | | | | | | | | Can’t Tell | | | |
| 5. (a) Have the authors identified all important confounding factors? | | | | | | | | Yes | | | | | | | | | | | | | | | | | | | | No | | | | | | | | | | | | | | | | | | Can’t Tell | | | |
| 5. (b) Have they taken account of the confounding factors in the design and/or analysis? | | | | | | | | Yes | | | | | | | | | | | | | | | | | | | | No | | | | | | | | | | | | | | | | | | Can’t Tell | | | |
| 6. (a) Was the follow up of subjects complete enough? | | | | | | | | Yes | | | | | | | | | | | | | | | | | | | | No | | | | | | | | | | | | | | | | | | Can’t Tell | | | |
| 6. (b) Was the follow up of subjects long enough? | | | | | | | | Yes | | | | | | | | | | | | | | | | | | | | No | | | | | | | | | | | | | | | | | | Can’t Tell | | | |
| **Section B: What are the results?** | | | | | | | | | | | | | | | | | | | | | | | | | | | | | | | | | | | | | | | | | | | | | | | | | |
| 7. What are the results of this study? | | | | | | | |  | | | | | | | | | | | | | | | | | | | | | | | | | | | | | | | | | | | | | | | | | |
| 8. How precise are the results? | | | | | | | |  | | | | | | | | | | | | | | | | | | | | | | | | | | | | | | | | | | | | | | | | | |
| 9. Do you believe the results? | | | | | | | | Yes | | | | | | | | | | | | | | | | | | | | No | | | | | | | | | | | | | | | | | | Can’t Tell | | | |
| **Section C: Will the results help locally?** | | | | | | | | | | | | | | | | | | | | | | | | | | | | | | | | | | | | | | | | | | | | | | | | | |
| 10. Can the results be applied to the local population? | | | | | | | | Yes | | | | | | | | | | | | | | | | | | | | No | | | | | | | | | | | | | | | | | | Can’t Tell | | | |
| 11. Do the results of this study fit with other available evidence? | | | | | | | | Yes | | | | | | | | | | | | | | | | | | | | No | | | | | | | | | | | | | | | | | | Can’t Tell | | | |
| 12. What are the implications of this study for practice? | | | | | | | | Yes | | | | | | | | | | | | | | | | | | | | No | | | | | | | | | | | | | | | | | | Can’t Tell | | | |
|  | | | | | | | | | | | | | | | | | | | | | | | | | | | | | | | | | | | | | | | | | | | | | | | | | |
| **B. The Scottish Intercollegiate Guidelines Network (SIGN) Methodology checklist: cohort study (last amended in 2014)**  **Website:** https://www.sign.ac.uk/checklists-and-notes.html | | | | | | | | | | | | | | | | | | | | | | | | | | | | | | | | | | | | | | | | | | | | | | | | | |
| Major Components | | | | | | | | | | | | | | | Response options | | | | | | | | | | | | | | | | | | | | | | | | | | | | | | | | | | |
| **SECTION 1: INTERNAL VALIDITY** | | | | | | | | | | | | | | | | | | | | | | | | | | | | | | | | | | | | | | | | | | | | | | | | | |
| 1.1. The study addresses an appropriate and clearly focused question. | | | | | | | | | | | | | | | Yes | | | | | | | | | | No | | | | | | | | | Can’t say | | | | | | | | | | / | | | | | |
| SELECTION OF SUBJECTS | | | | | | | | | | | | | | | | | | | | | | | | | | | | | | | | | | | | | | | | | | | | | | | | | |
| 1.2. The two groups being studied are selected from source populations that are comparable in all respects other than the factor under investigation. | | | | | | | | | | | | | | | Yes | | | | | | | | | | No | | | | | | | | | Can’t say | | | | | | | | | | Does not apply | | | | | |
| 1.3. The study indicates how many of the people asked to take part did so, in each of the groups being studied. | | | | | | | | | | | | | | | Yes | | | | | | | | | | No | | | | | | | | | / | | | | | | | | | | Does not apply | | | | | |
| 1.4. The likelihood that some eligible subjects might have the outcome at the time of enrolment is assessed and taken into account in the analysis. | | | | | | | | | | | | | | | Yes | | | | | | | | | | No | | | | | | | | | Can’t say | | | | | | | | | | Does not apply | | | | | |
| 1.5. What percentage of individuals or clusters recruited into each arm of the study dropped out before the study was completed. | | | | | | | | | | | | | | |  | | | | | | | | | | | | | | | | | | | | | | | | | | | | | | | | | | |
| 1.6. Comparison is made between full participants and those lost to follow up, by exposure status. | | | | | | | | | | | | | | | Yes | | | | | | | | | | No | | | | | | | | | Can’t say | | | | | | | | | | Does not apply | | | | | |
| ASSESSMENT | | | | | | | | | | | | | | | | | | | | | | | | | | | | | | | | | | | | | | | | | | | | | | | | | |
| 1.7. The outcomes are clearly defined. | | | | | | | | | | | | | | | Yes | | | | | | | | | | No | | | | | | | | | Can’t say | | | | | | | | | | / | | | | | |
| 1.8. The assessment of outcome is made blind to exposure status. If the study is retrospective this may not be applicable. | | | | | | | | | | | | | | | Yes | | | | | | | | | | No | | | | | | | | | Can’t say | | | | | | | | | | Does not apply | | | | | |
| 1.9. Where blinding was not possible, there is some recognition that knowledge of exposure status could have influenced the assessment of outcome. | | | | | | | | | | | | | | | Yes | | | | | | | | | | No | | | | | | | | | Can’t say | | | | | | | | | | / | | | | | |
| 1.10. The method of assessment of exposure is reliable. | | | | | | | | | | | | | | | Yes | | | | | | | | | | No | | | | | | | | | Can’t say | | | | | | | | | | / | | | | | |
| 1.11. Evidence from other sources is used to demonstrate that the method of outcome assessment is valid and reliable. | | | | | | | | | | | | | | | Yes | | | | | | | | | | No | | | | | | | | | Can’t say | | | | | | | | | | Does not apply | | | | | |
| 1.12. Exposure level or prognostic factor is assessed more than once. | | | | | | | | | | | | | | | Yes | | | | | | | | | | No | | | | | | | | | Can’t say | | | | | | | | | | Does not apply | | | | | |
| CONFOUNDING | | | | | | | | | | | | | | | | | | | | | | | | | | | | | | | | | | | | | | | | | | | | | | | | | |
| 1.13. The main potential confounders are identified and taken into account in the design and analysis. | | | | | | | | | | | | | | | Yes | | | | | | | | | | No | | | | | | | | | Can’t say | | | | | | | | | | / | | | | | |
| STATISTICAL ANALYSIS | | | | | | | | | | | | | | | | | | | | | | | | | | | | | | | | | | | | | | | | | | | | | | | | | |
| 1.14. Have confidence intervals been provided? | | | | | | | | | | | | | | | Yes | | | | | | | | | | No | | | | | | | | | / | | | | | | | | | | / | | | | | |
| **SECTION 2: OVERALL ASSESSMENT OF THE STUDY** | | | | | | | | | | | | | | | | | | | | | | | | | | | | | | | | | | | | | | | | | | | | | | | | | |
| 2.1 How well was the study done to minimise the risk of bias or confounding? | | | | | | | | | | | | | | | High quality (++) | | | | | | | | | | | | | | | | | | | Acceptable (+) | | | | | | | | | | Unacceptable – reject 0 | | | | | |
| 2.2. Taking into account clinical considerations, your evaluation of the methodology used, and the statistical power of the study, do you think there is clear evidence of an association between exposure and outcome? | | | | | | | | | | | | | | | Yes | | | | | | | | | | No | | | | | | | | | Can’t say | | | | | | | | | | / | | | | | |
| 2.3. Are the results of this study directly applicable to the patient group targeted in this guideline? | | | | | | | | | | | | | | | Yes | | | | | | | | | | No | | | | | | | | | / | | | | | | | | | | / | | | | | |
| 2.4. Notes. Summarise the authors conclusions. Add any comments on your own assessment of the study, and the extent to which it answers your question and mention any areas of uncertainty raised above. | | | | | | | | | | | | | | | | | | | | | | | | | | | | | | | | | | | | | | | | | | | | | | | | | |
|  | | | | | | | | | | | | | | | | | | | | | | | | | | | | | | | | | | | | | | | | | | | | | | | | | |
| **C. The National Institutes of Health (NIH) quality assessment tool for observational cohort and cross-sectional studies**  **Website:** https://www.nhlbi.nih.gov/health-topics/study-quality-assessment-tools | | | | | | | | | | | | | | | | | | | | | | | | | | | | | | | | | | | | | | | | | | | | | | | | | |
| Major Components | | | Response options | | | | | | | | | | | | | | | | | | | | | | | | | | | | | | | | | | | | | | | | | | | | | | |
| 1. Was the research question or objective in this paper clearly stated? | | | Yes | | | No | | | | | | Cannot Determine/ Not Applicable/ Not Reported | | | | | | | | | | | | | | | | | | | | | | | | | | | | | | | | | | | | | |
| 2. Was the study population clearly specified and defined? | | | Yes | | | No | | | | | | Cannot Determine/ Not Applicable/ Not Reported | | | | | | | | | | | | | | | | | | | | | | | | | | | | | | | | | | | | | |
| 3. Was the participation rate of eligible persons at least 50%? | | | Yes | | | No | | | | | | Cannot Determine/ Not Applicable/ Not Reported | | | | | | | | | | | | | | | | | | | | | | | | | | | | | | | | | | | | | |
| 4. Were all the subjects selected or recruited from the same or similar populations (including the same time period)? Were inclusion and exclusion criteria for being in the study prespecified and applied uniformly to all participants? | | | Yes | | | No | | | | | | Cannot Determine/ Not Applicable/ Not Reported | | | | | | | | | | | | | | | | | | | | | | | | | | | | | | | | | | | | | |
| 5. Was a sample size justification, power description, or variance and effect estimates provided? | | | Yes | | | No | | | | | | Cannot Determine/ Not Applicable/ Not Reported | | | | | | | | | | | | | | | | | | | | | | | | | | | | | | | | | | | | | |
| 6. For the analyses in this paper, were the exposure(s) of interest measured prior to the outcome(s) being measured? | | | Yes | | | No | | | | | | Cannot Determine/ Not Applicable/ Not Reported | | | | | | | | | | | | | | | | | | | | | | | | | | | | | | | | | | | | | |
| 7. Was the timeframe sufficient so that one could reasonably expect to see an association between exposure and outcome if it existed? | | | Yes | | | No | | | | | | Cannot Determine/ Not Applicable/ Not Reported | | | | | | | | | | | | | | | | | | | | | | | | | | | | | | | | | | | | | |
| 8. For exposures that can vary in amount or level, did the study examine different levels of the exposure as related to the outcome (e.g., categories of exposure, or exposure measured as continuous variable)? | | | Yes | | | No | | | | | | Cannot Determine/ Not Applicable/ Not Reported | | | | | | | | | | | | | | | | | | | | | | | | | | | | | | | | | | | | | |
| 9. Were the exposure measures (independent variables) clearly defined, valid, reliable, and implemented consistently across all study participants? | | | Yes | | | No | | | | | | Cannot Determine/ Not Applicable/ Not Reported | | | | | | | | | | | | | | | | | | | | | | | | | | | | | | | | | | | | | |
| 10. Was the exposure(s) assessed more than once over time? | | | Yes | | | No | | | | | | Cannot Determine/ Not Applicable/ Not Reported | | | | | | | | | | | | | | | | | | | | | | | | | | | | | | | | | | | | | |
| 11. Were the outcome measures (dependent variables) clearly defined, valid, reliable, and implemented consistently across all study participants? | | | Yes | | | No | | | | | | Cannot Determine/ Not Applicable/ Not Reported | | | | | | | | | | | | | | | | | | | | | | | | | | | | | | | | | | | | | |
| 12. Were the outcome assessors blinded to the exposure status of participants? | | | Yes | | | No | | | | | | Cannot Determine/ Not Applicable/ Not Reported | | | | | | | | | | | | | | | | | | | | | | | | | | | | | | | | | | | | | |
| 13. Was loss to follow-up after baseline 20% or less? | | | Yes | | | No | | | | | | Cannot Determine/ Not Applicable/ Not Reported | | | | | | | | | | | | | | | | | | | | | | | | | | | | | | | | | | | | | |
| 14. Were key potential confounding variables measured and adjusted statistically for their impact on the relationship between exposure(s) and outcome(s)? | | | Yes | | | No | | | | | | Cannot Determine/ Not Applicable/ Not Reported | | | | | | | | | | | | | | | | | | | | | | | | | | | | | | | | | | | | | |
| Quality Rating | | | Good | | | Fair | | | | | | Poor | | | | | | | | | | | | | | | | | | | | | | | | | | | | | | | | | | | | | |
| Additional Comments (If Poor, please state why): | | | | | | | | | | | | | | | | | | | | | | | | | | | | | | | | | | | | | | | | | | | | | | | | | |
|  | | | | | | | | | | | | | | | | | | | | | | | | | | | | | | | | | | | | | | | | | | | | | | | | | |
| **D. The Newcastle-Ottawa Scale (NOS) for cohort study**  **Website:** http://www.ohri.ca/programs/clinical_epidemiology/oxford.asp | | | | | | | | | | | | | | | | | | | | | | | | | | | | | | | | | | | | | | | | | | | | | | | | | |
| Major Components | | | | | | | | | | | | | | | | | | | | | | | | | | | | | | | | | | | | Response options | | | | | | | | | | | | | |
| **Selection** | | | | | | | | | | | | | | | | | | | | | | | | | | | | | | | | | | | | | | | | | | | | | | | | | |
| 1. Representativeness of the exposed cohort  1) truly representative of the average _______________ (describe) in the community  2) somewhat representative of the average ______________ in the community  3) selected group of users eg nurses, volunteers  4) no description of the derivation of the cohort | | | | | | | | | | | | | | | | | | | | | | | | | | | | | | | | | | | | ☆  ☆  /  / | | | | | | | | | | | | | |
| 2. Selection of the non exposed cohort  1) drawn from the same community as the exposed cohort  2) drawn from a different source  3) no description of the derivation of the non exposed cohort | | | | | | | | | | | | | | | | | | | | | | | | | | | | | | | | | | | | ☆  /  / | | | | | | | | | | | | | |
| 3. Ascertainment of exposure  1) secure record (eg surgical records)  2) structured interview   3) written self report  4) no description | | | | | | | | | | | | | | | | | | | | | | | | | | | | | | | | | | | | ☆  ☆  /  / | | | | | | | | | | | | | |
| 4. Demonstration that outcome of interest was not present at start of study  1) yes  2) no | | | | | | | | | | | | | | | | | | | | | | | | | | | | | | | | | | | | ☆  / | | | | | | | | | | | | | |
| **Comparability*** | | | | | | | | | | | | | | | | | | | | | | | | | | | | | | | | | | | | | | | | | | | | | | | | | |
| 5. Comparability of cohorts on the basis of the design or analysis  1) study controls for _____________ (select the most important factor)  2) study controls for any additional factor (This criteria could be modified to indicate specific control for a second important factor.) | | | | | | | | | | | | | | | | | | | | | | | | | | | | | | | | | | | | ☆  ☆ | | | | | | | | | | | | | |
| **Outcome** | | | | | | | | | | | | | | | | | | | | | | | | | | | | | | | | | | | | | | | | | | | | | | | | | |
| 6. Assessment of outcome  1) independent blind assessment  2) record linkage  3) self report  4) no description | | | | | | | | | | | | | | | | | | | | | | | | | | | | | | | | | | | | ☆  ☆  /  / | | | | | | | | | | | | | |
| 7. Was follow-up long enough for outcomes to occur  1) yes (select an adequate follow up period for outcome of interest)  2) no | | | | | | | | | | | | | | | | | | | | | | | | | | | | | | | | | | | | ☆  / | | | | | | | | | | | | | |
| 8. Adequacy of follow up of cohorts  1) complete follow up - all subjects accounted for ¯  2) subjects lost to follow up unlikely to introduce bias - small number lost - > ____ % (select an adequate %) follow up, or description provided of those lost)  3) follow up rate < ____% (select an adequate %) and no description of those lost  4) no statement | | | | | | | | | | | | | | | | | | | | | | | | | | | | | | | | | | | | ☆  ☆  /  / | | | | | | | | | | | | | |
| *, A study can be awarded a maximum of one star for each numbered item within the Selection and Exposure categories; a maximum of two stars can be given for Comparability. | | | | | | | | | | | | | | | | | | | | | | | | | | | | | | | | | | | | | | | | | | | | | | | | | |
|  | | | | | | | | | | | | | | | | | | | | | | | | | | | | | | | | | | | | | | | | | | | | | | | | | |
| **E. The Joanna Briggs Institute (JBI) Critical Appraisal Checklist for cohort study (last amended in 2017)**  **Website:** https://joannabriggs.org/critical_appraisal_tools  https://wiki.joannabriggs.org/display/MANUAL/Appendix+7.1++Critical+appraisal+checklist+for+cohort+studies | | | | | | | | | | | | | | | | | | | | | | | | | | | | | | | | | | | | | | | | | | | | | | | | | |
| Major Components | | | | | | | | | | | | | | | | Response options | | | | | | | | | | | | | | | | | | | | | | | | | | | | | | | | | |
| 1. Were the two groups similar and recruited from the same population? | | | | | | | | | | | | | | | | Yes | | | | | | | | | | No | | | | | | | | | | Unclear | | | | | | | | | | | Not applicable | | |
| 2. Were the exposures measured similarly to assign people to both exposed and unexposed groups? | | | | | | | | | | | | | | | | Yes | | | | | | | | | | No | | | | | | | | | | Unclear | | | | | | | | | | | Not applicable | | |
| 3. Was the exposure measured in a valid and reliable way? | | | | | | | | | | | | | | | | Yes | | | | | | | | | | No | | | | | | | | | | Unclear | | | | | | | | | | | Not applicable | | |
| 4. Were confounding factors identified? | | | | | | | | | | | | | | | | Yes | | | | | | | | | | No | | | | | | | | | | Unclear | | | | | | | | | | | Not applicable | | |
| 5. Were strategies to deal with confounding factors stated? | | | | | | | | | | | | | | | | Yes | | | | | | | | | | No | | | | | | | | | | Unclear | | | | | | | | | | | Not applicable | | |
| 6. Were the groups/participants free of the outcome at the start of the study (or at the moment of exposure)? | | | | | | | | | | | | | | | | Yes | | | | | | | | | | No | | | | | | | | | | Unclear | | | | | | | | | | | Not applicable | | |
| 7. Were the outcomes measured in a valid and reliable way? | | | | | | | | | | | | | | | | Yes | | | | | | | | | | No | | | | | | | | | | Unclear | | | | | | | | | | | Not applicable | | |
| 8. Was the follow up time reported and sufficient to be long enough for outcomes to occur? | | | | | | | | | | | | | | | | Yes | | | | | | | | | | No | | | | | | | | | | Unclear | | | | | | | | | | | Not applicable | | |
| 9. Was follow up complete, and if not, were the reasons to loss to follow up described and explored? | | | | | | | | | | | | | | | | Yes | | | | | | | | | | No | | | | | | | | | | Unclear | | | | | | | | | | | Not applicable | | |
| 10. Were strategies to address incomplete follow up utilized? | | | | | | | | | | | | | | | | Yes | | | | | | | | | | No | | | | | | | | | | Unclear | | | | | | | | | | | Not applicable | | |
| 11. Was appropriate statistical analysis used? | | | | | | | | | | | | | | | | Yes | | | | | | | | | | No | | | | | | | | | | Unclear | | | | | | | | | | | Not applicable | | |
| Overall appraisal: Include □ Exclude □ Seek further info □ | | | | | | | | | | | | | | | | | | | | | | | | | | | | | | | | | | | | | | | | | | | | | | | | | |
|  | | | | | | | | | | | | | | | | | | | | | | | | | | | | | | | | | | | | | | | | | | | | | | | | | |
| **F. The Critical Appraisal Skills Programme (CASP) Checklist for case control study (last amended in 2018)**  **Website:** https://casp-uk.net/casp-tools-checklists/ | | | | | | | | | | | | | | | | | | | | | | | | | | | | | | | | | | | | | | | | | | | | | | | | | |
| Major Components | | | | | | | | | | | | | | | | | | | | | | | | Response options | | | | | | | | | | | | | | | | | | | | | | | | | |
| **Section A: Are the results of the trial valid?** | | | | | | | | | | | | | | | | | | | | | | | | | | | | | | | | | | | | | | | | | | | | | | | | | |
| 1. Did the study address a clearly focused issue? | | | | | | | | | | | | | | | | | | | | | | | | Yes | | | | | | | | | | | | | No | | | | | | | | | | | Can’t Tell | |
| 2. Did the authors use an appropriate method to answer their question? | | | | | | | | | | | | | | | | | | | | | | | | Yes | | | | | | | | | | | | | No | | | | | | | | | | | Can’t Tell | |
| Is it worth continuing? | | | | | | | | | | | | | | | | | | | | | | | | | | | | | | | | | | | | | | | | | | | | | | | | | |
| 3. Were the cases recruited in an acceptable way? | | | | | | | | | | | | | | | | | | | | | | | | Yes | | | | | | | | | | | | | No | | | | | | | | | | | Can’t Tell | |
| 4. Were the controls selected in an acceptable way? | | | | | | | | | | | | | | | | | | | | | | | | Yes | | | | | | | | | | | | | No | | | | | | | | | | | Can’t Tell | |
| 5. Was the exposure accurately measured to minimise bias? | | | | | | | | | | | | | | | | | | | | | | | | Yes | | | | | | | | | | | | | No | | | | | | | | | | | Can’t Tell | |
| 6. (a) Aside from the experimental intervention, were the groups treated equally? | | | | | | | | | | | | | | | | | | | | | | | | List: | | | | | | | | | | | | | | | | | | | | | | | | | |
| 6. (b) Have the authors taken account of the potential confounding factors in the design and/or in their analysis? | | | | | | | | | | | | | | | | | | | | | | | | Yes | | | | | | | | | | | | | No | | | | | | | | | | | Can’t Tell | |
| **Section B: What are the results?** | | | | | | | | | | | | | | | | | | | | | | | | | | | | | | | | | | | | | | | | | | | | | | | | | |
| 7. How large was the treatment effect? | | | | | | | | | | | | | | | | | | | | | | | |  | | | | | | | | | | | | | | | | | | | | | | | | | |
| 8. How precise was the estimate of the treatment effect? | | | | | | | | | | | | | | | | | | | | | | | |  | | | | | | | | | | | | | | | | | | | | | | | | | |
| 9. Do you believe the results? | | | | | | | | | | | | | | | | | | | | | | | | Yes | | | | | | | | | | | | | No | | | | | | | | | | | / | |
| **Section C: Will the results help locally?** | | | | | | | | | | | | | | | | | | | | | | | | | | | | | | | | | | | | | | | | | | | | | | | | | |
| 10. Can the results be applied to the local population? | | | | | | | | | | | | | | | | | | | | | | | | Yes | | | | | | | | | | | | | No | | | | | | | | | | | Can’t Tell | |
| 11. Do the results of this study fit with other available evidence? | | | | | | | | | | | | | | | | | | | | | | | | Yes | | | | | | | | | | | | | No | | | | | | | | | | | Can’t Tell | |
|  | | | | | | | | | | | | | | | | | | | | | | | | | | | | | | | | | | | | | | | | | | | | | | | | | |
| **G. The Scottish Intercollegiate Guidelines Network (SIGN) Methodology checklist: case-control study (last amended in 2014)**  **Website:** https://www.sign.ac.uk/checklists-and-notes.html | | | | | | | | | | | | | | | | | | | | | | | | | | | | | | | | | | | | | | | | | | | | | | | | | |
| Major Components | | | | | | | | | Response options | | | | | | | | | | | | | | | | | | | | | | | | | | | | | | | | | | | | | | | | |
| **SECTION 1: INTERNAL VALIDITY** | | | | | | | | | | | | | | | | | | | | | | | | | | | | | | | | | | | | | | | | | | | | | | | | | |
| 1.1. The study addresses an appropriate and clearly focused question. | | | | | | | | | Yes | | | | | | | | | No | | | | | | | | | | | Can’t say | | | | | | | | | | | | | | | / | | | | | |
| SELECTION OF SUBJECTS | | | | | | | | | | | | | | | | | | | | | | | | | | | | | | | | | | | | | | | | | | | | | | | | | |
| 1.2. The cases and controls are taken from comparable populations. | | | | | | | | | Yes | | | | | | | | | No | | | | | | | | | | | Can’t say | | | | | | | | | | | | | | | / | | | | | |
| 1.3. The same exclusion criteria are used for both cases and controls. | | | | | | | | | Yes | | | | | | | | | No | | | | | | | | | | | Can’t say | | | | | | | | | | | | | | | / | | | | | |
| 1.4. What percentage of each group (cases and controls) participated in the study? | | | | | | | | | Cases: | | | | | | | | | | | | | | | | | | | | Controls: | | | | | | | | | | | | | | | | | | | | |
| 1.5. Comparison is made between participants and non-participants to establish their similarities or differences. | | | | | | | | | Yes | | | | | | | | | No | | | | | | | | | | | Can’t say | | | | | | | | | | | | | | | / | | | | | |
| 1.6. Cases are clearly defined and differentiated from controls. | | | | | | | | | Yes | | | | | | | | | No | | | | | | | | | | | Can’t say | | | | | | | | | | | | | | | / | | | | | |
| 1.7. It is clearly established that controls are non-cases. | | | | | | | | | Yes | | | | | | | | | No | | | | | | | | | | | Can’t say | | | | | | | | | | | | | | | / | | | | | |
| ASSESSMENT | | | | | | | | | | | | | | | | | | | | | | | | | | | | | | | | | | | | | | | | | | | | | | | | | |
| 1.8. Measures will have been taken to prevent knowledge of primary exposure influencing case ascertainment. | | | | | | | | | Yes | | | | | | | | | No | | | | | | | | | | | Can’t say | | | | | | | | | | | | | | | / | | | | | |
| 1.9. Exposure status is measured in a standard, valid and reliable way. | | | | | | | | | Yes | | | | | | | | | No | | | | | | | | | | | Can’t say | | | | | | | | | | | | | | | Does not apply | | | | | |
| CONFOUNDING | | | | | | | | | | | | | | | | | | | | | | | | | | | | | | | | | | | | | | | | | | | | | | | | | |
| 1.10. The main potential confounders are identified and taken into account in the design and analysis. | | | | | | | | | Yes | | | | | | | | | No | | | | | | | | | | | Can’t say | | | | | | | | | | | | | | | / | | | | | |
| STATISTICAL ANALYSIS | | | | | | | | | | | | | | | | | | | | | | | | | | | | | | | | | | | | | | | | | | | | | | | | | |
| 1.11. Confidence intervals are provided. | | | | | | | | | Yes | | | | | | | | | No | | | | | | | | | | | / | | | | | | | | | | | | | | | / | | | | | |
| **SECTION 2: OVERALL ASSESSMENT OF THE STUDY** | | | | | | | | | | | | | | | | | | | | | | | | | | | | | | | | | | | | | | | | | | | | | | | | | |
| 2.1. How well was the study done to minimise the risk of bias or confounding? | | | | | | | | | High quality (++) | | | | | | | | | | | | | | | | | | | | Acceptable (+) | | | | | | | | | | | | | | | Unacceptable – reject 0 | | | | | |
| 2.2. Taking into account clinical considerations, your evaluation of the methodology used, and the statistical power of the study, do you think there is clear evidence of an association between exposure and outcome? | | | | | | | | | Yes | | | | | | | | | No | | | | | | | | | | | Can’t say | | | | | | | | | | | | | | | / | | | | | |
| 2.3. Are the results of this study directly applicable to the patient group targeted by this guideline? | | | | | | | | | Yes | | | | | | | | | No | | | | | | | | | | | / | | | | | | | | | | | | | | | / | | | | | |
| 2.4. Notes. Summarise the authors conclusions. Add any comments on your own assessment of the study, and the extent to which it answers your question and mention any areas of uncertainty raised above. | | | | | | | | | | | | | | | | | | | | | | | | | | | | | | | | | | | | | | | | | | | | | | | | | |
|  | | | | | | | | | | | | | | | | | | | | | | | | | | | | | | | | | | | | | | | | | | | | | | | | | |
| **H. The National Institutes of Health (NIH) quality assessment tool of case-control study**  **Website:** https://www.nhlbi.nih.gov/health-topics/study-quality-assessment-tools | | | | | | | | | | | | | | | | | | | | | | | | | | | | | | | | | | | | | | | | | | | | | | | | | |
| Major Components | | | | Response options | | | | | | | | | | | | | | | | | | | | | | | | | | | | | | | | | | | | | | | | | | | | | |
| 1. Was the research question or objective in this paper clearly stated and appropriate? | | | | Yes | | | No | | | | | | Cannot Determine/ Not Applicable/ Not Reported | | | | | | | | | | | | | | | | | | | | | | | | | | | | | | | | | | | | |
| 2. Was the study population clearly specified and defined? | | | | Yes | | | No | | | | | | Cannot Determine/ Not Applicable/ Not Reported | | | | | | | | | | | | | | | | | | | | | | | | | | | | | | | | | | | | |
| 3. Did the authors include a sample size justification? | | | | Yes | | | No | | | | | | Cannot Determine/ Not Applicable/ Not Reported | | | | | | | | | | | | | | | | | | | | | | | | | | | | | | | | | | | | |
| 4. Were controls selected or recruited from the same or similar population that gave rise to the cases (including the same timeframe)? | | | | Yes | | | No | | | | | | Cannot Determine/ Not Applicable/ Not Reported | | | | | | | | | | | | | | | | | | | | | | | | | | | | | | | | | | | | |
| 5. Were the definitions, inclusion and exclusion criteria, algorithms or processes used to identify or select cases and controls valid, reliable, and implemented consistently across all study participants? | | | | Yes | | | No | | | | | | Cannot Determine/ Not Applicable/ Not Reported | | | | | | | | | | | | | | | | | | | | | | | | | | | | | | | | | | | | |
| 6. Were the cases clearly defined and differentiated from controls? | | | | Yes | | | No | | | | | | Cannot Determine/ Not Applicable/ Not Reported | | | | | | | | | | | | | | | | | | | | | | | | | | | | | | | | | | | | |
| 7. If less than 100 percent of eligible cases and/or controls were selected for the study, were the cases and/or controls randomly selected from those eligible? | | | | Yes | | | No | | | | | | Cannot Determine/ Not Applicable/ Not Reported | | | | | | | | | | | | | | | | | | | | | | | | | | | | | | | | | | | | |
| 8. Was there use of concurrent controls? | | | | Yes | | | No | | | | | | Cannot Determine/ Not Applicable/ Not Reported | | | | | | | | | | | | | | | | | | | | | | | | | | | | | | | | | | | | |
| 9. Were the investigators able to confirm that the exposure/risk occurred prior to the development of the condition or event that defined a participant as a case? | | | | Yes | | | No | | | | | | Cannot Determine/ Not Applicable/ Not Reported | | | | | | | | | | | | | | | | | | | | | | | | | | | | | | | | | | | | |
| 10. Were the measures of exposure/risk clearly defined, valid, reliable, and implemented consistently (including the same time period) across all study participants? | | | | Yes | | | No | | | | | | Cannot Determine/ Not Applicable/ Not Reported | | | | | | | | | | | | | | | | | | | | | | | | | | | | | | | | | | | | |
| 11. Were the assessors of exposure/risk blinded to the case or control status of participants? | | | | Yes | | | No | | | | | | Cannot Determine/ Not Applicable/ Not Reported | | | | | | | | | | | | | | | | | | | | | | | | | | | | | | | | | | | | |
| 12. Were key potential confounding variables measured and adjusted statistically in the analyses? If matching was used, did the investigators account for matching during study analysis? | | | | Yes | | | No | | | | | | Cannot Determine/ Not Applicable/ Not Reported | | | | | | | | | | | | | | | | | | | | | | | | | | | | | | | | | | | | |
| **Quality Rating:** | | | | Good | | | Fair | | | | | | Poor | | | | | | | | | | | | | | | | | | | | | | | | | | | | | | | | | | | | |
| Additional Comments (If Poor, please state why): | | | | | | | | | | | | | | | | | | | | | | | | | | | | | | | | | | | | | | | | | | | | | | | | | |
|  | | | | | | | | | | | | | | | | | | | | | | | | | | | | | | | | | | | | | | | | | | | | | | | | | |
| **I. The Joanna Briggs Institute (JBI) Critical Appraisal Checklist for case-control study (last amended in 2017)**  **Website:** https://joannabriggs.org/critical_appraisal_tools  https://wiki.joannabriggs.org/display/MANUAL/Appendix+7.2+Critical+appraisal+checklist+for+case-control+studies | | | | | | | | | | | | | | | | | | | | | | | | | | | | | | | | | | | | | | | | | | | | | | | | | |
| Major Components | | | | | | | | | Response options | | | | | | | | | | | | | | | | | | | | | | | | | | | | | | | | | | | | | | | | |
| 1. Were the groups comparable other than the presence of disease in cases or the absence of disease in controls? | | | | | | | | | Yes | | | | | | | | | | | | | No | | | | | | | | | Unclear | | | | | | | | | | | Not applicable | | | | | | | |
| 2. Were cases and controls matched appropriately? | | | | | | | | | Yes | | | | | | | | | | | | | No | | | | | | | | | Unclear | | | | | | | | | | | Not applicable | | | | | | | |
| 3. Were the same criteria used for identification of cases and controls? | | | | | | | | | Yes | | | | | | | | | | | | | No | | | | | | | | | Unclear | | | | | | | | | | | Not applicable | | | | | | | |
| 4. Was exposure measured in a standard, valid and reliable way? | | | | | | | | | Yes | | | | | | | | | | | | | No | | | | | | | | | Unclear | | | | | | | | | | | Not applicable | | | | | | | |
| 5. Was exposure measured in the same way for cases and controls? | | | | | | | | | Yes | | | | | | | | | | | | | No | | | | | | | | | Unclear | | | | | | | | | | | Not applicable | | | | | | | |
| 6. Were confounding factors identified? | | | | | | | | | Yes | | | | | | | | | | | | | No | | | | | | | | | Unclear | | | | | | | | | | | Not applicable | | | | | | | |
| 7. Were strategies to deal with confounding factors stated? | | | | | | | | | Yes | | | | | | | | | | | | | No | | | | | | | | | Unclear | | | | | | | | | | | Not applicable | | | | | | | |
| 8. Were outcomes assessed in a standard, valid and reliable way for cases and controls? | | | | | | | | | Yes | | | | | | | | | | | | | No | | | | | | | | | Unclear | | | | | | | | | | | Not applicable | | | | | | | |
| 9. Was the exposure period of interest long enough to be meaningful? | | | | | | | | | Yes | | | | | | | | | | | | | No | | | | | | | | | Unclear | | | | | | | | | | | Not applicable | | | | | | | |
| 10. Was appropriate statistical analysis used? | | | | | | | | | Yes | | | | | | | | | | | | | No | | | | | | | | | Unclear | | | | | | | | | | | Not applicable | | | | | | | |
| Overall appraisal: Include □ Exclude □ Seek further info □ | | | | | | | | | | | | | | | | | | | | | | | | | | | | | | | | | | | | | | | | | | | | | | | | | |
|  | | | | | | | | | | | | | | | | | | | | | | | | | | | | | | | | | | | | | | | | | | | | | | | | | |
| **J. The Newcastle-Ottawa Scale (NOS) for case-control study**  **Website:** http://www.ohri.ca/programs/clinical_epidemiology/oxford.asp | | | | | | | | | | | | | | | | | | | | | | | | | | | | | | | | | | | | | | | | | | | | | | | | | |
| Major Components | | | | | | | | | | | | | | | | | | | | | | | | | | | | | | | | | | | | | | Response options | | | | | | | | | | | |
| Selection | | | | | | | | | | | | | | | | | | | | | | | | | | | | | | | | | | | | | | | | | | | | | | | | | |
| 1. Is the case definition adequate?  1) yes, with independent validation  2) yes, eg record linkage or based on self reports  3) no description | | | | | | | | | | | | | | | | | | | | | | | | | | | | | | | | | | | | | | ☆  /  / | | | | | | | | | | | |
| 2. Representativeness of the cases  1) consecutive or obviously representative series of cases  2) potential for selection biases or not stated | | | | | | | | | | | | | | | | | | | | | | | | | | | | | | | | | | | | | | ☆  / | | | | | | | | | | | |
| 3. Selection of Controls  1) community controls  2) hospital controls  3) no description | | | | | | | | | | | | | | | | | | | | | | | | | | | | | | | | | | | | | | ☆  /  / | | | | | | | | | | | |
| 4. Definition of Controls  1) no history of disease (endpoint)  2) no description of source | | | | | | | | | | | | | | | | | | | | | | | | | | | | | | | | | | | | | | ☆  / | | | | | | | | | | | |
| Comparability* | | | | | | | | | | | | | | | | | | | | | | | | | | | | | | | | | | | | | | | | | | | | | | | | | |
| 5. Comparability of cases and controls on the basis of the design or analysis  1) study controls for _______________ (Select the most important factor.)  2) study controls for any additional factor (This criteria could be modified to indicate specific control for a second important factor.) | | | | | | | | | | | | | | | | | | | | | | | | | | | | | | | | | | | | | | ☆  ☆ | | | | | | | | | | | |
| Exposure | | | | | | | | | | | | | | | | | | | | | | | | | | | | | | | | | | | | | | | | | | | | | | | | | |
| 6. Ascertainment of exposure  1) secure record (eg surgical records)  2) structured interview where blind to case/control status  3) interview not blinded to case/control status  4) written self report or medical record only  5) no description | | | | | | | | | | | | | | | | | | | | | | | | | | | | | | | | | | | | | | ☆  ☆  /  /  / | | | | | | | | | | | |
| 7. Same method of ascertainment for cases and controls  1) yes  2) no | | | | | | | | | | | | | | | | | | | | | | | | | | | | | | | | | | | | | | ☆  / | | | | | | | | | | | |
| 8. Non-Response rate  1) same rate for both groups  2) non respondents described  3) rate different and no designation | | | | | | | | | | | | | | | | | | | | | | | | | | | | | | | | | | | | | | ☆  /  / | | | | | | | | | | | |
| *, A study can be awarded a maximum of one star for each numbered item within the Selection and Exposure categories; a maximum of two stars can be given for Comparability. | | | | | | | | | | | | | | | | | | | | | | | | | | | | | | | | | | | | | | | | | | | | | | | | | |
|  | | | | | | | | | | | | | | | | | | | | | | | | | | | | | | | | | | | | | | | | | | | | | | | | | |
| **K. The Joanna Briggs Institute (JBI) Critical Appraisal Checklist for analytical cross-sectional study (last amended in 2017)**  **Website:** https://joannabriggs.org/critical_appraisal_tools  https://wiki.joannabriggs.org/display/MANUAL/Appendix+7.5+Critical+appraisal+checklist+for+analytical+cross-sectional+studies | | | | | | | | | | | | | | | | | | | | | | | | | | | | | | | | | | | | | | | | | | | | | | | | | |
| Major Components | | | | | | | | | Response options | | | | | | | | | | | | | | | | | | | | | | | | | | | | | | | | | | | | | | | | |
| 1. Were the criteria for inclusion in the sample clearly defined? | | | | | | | | | Yes | | | | | | | | | | No | | | | | | | | | | Unclear | | | | | | | | | | | Not applicable | | | | | | | | | |
| 2. Were the study subjects and the setting described in detail? | | | | | | | | | Yes | | | | | | | | | | No | | | | | | | | | | Unclear | | | | | | | | | | | Not applicable | | | | | | | | | |
| 3. Was the exposure measured in a valid and reliable way? | | | | | | | | | Yes | | | | | | | | | | No | | | | | | | | | | Unclear | | | | | | | | | | | Not applicable | | | | | | | | | |
| 4. Were objective, standard criteria used for measurement of the condition? | | | | | | | | | Yes | | | | | | | | | | No | | | | | | | | | | Unclear | | | | | | | | | | | Not applicable | | | | | | | | | |
| 5. Were confounding factors identified? | | | | | | | | | Yes | | | | | | | | | | No | | | | | | | | | | Unclear | | | | | | | | | | | Not applicable | | | | | | | | | |
| 6. Were strategies to deal with confounding factors stated? | | | | | | | | | Yes | | | | | | | | | | No | | | | | | | | | | Unclear | | | | | | | | | | | Not applicable | | | | | | | | | |
| 7. Were the outcomes measured in a valid and reliable way? | | | | | | | | | Yes | | | | | | | | | | No | | | | | | | | | | Unclear | | | | | | | | | | | Not applicable | | | | | | | | | |
| 8. Was appropriate statistical analysis used? | | | | | | | | | Yes | | | | | | | | | | No | | | | | | | | | | Unclear | | | | | | | | | | | Not applicable | | | | | | | | | |
| Overall appraisal: Include □ Exclude □ Seek further info □ | | | | | | | | | | | | | | | | | | | | | | | | | | | | | | | | | | | | | | | | | | | | | | | | | |
|  | | | | | | | | | | | | | | | | | | | | | | | | | | | | | | | | | | | | | | | | | | | | | | | | | |
| **L. The Appraisal tool for Cross-Sectional Studies (AXIS tool; last introduced on December 8, 2016)** | | | | | | | | | | | | | | | | | | | | | | | | | | | | | | | | | | | | | | | | | | | | | | | | | |
| Major Components | | | | | | | | | Response options | | | | | | | | | | | | | | | | | | | | | | | | | | | | | | | | | | | | | | | | |
| Introduction | | | | | | | | | | | | | | | | | | | | | | | | | | | | | | | | | | | | | | | | | | | | | | | | | |
| 1. Were the aims/objectives of the study clear? | | | | | | | | | Yes | | | | | | | | | | | | | | | No | | | | | | | | | | | | | Do not know/ comment | | | | | | | | | | | | |
| Methods | | | | | | | | | | | | | | | | | | | | | | | | | | | | | | | | | | | | | | | | | | | | | | | | | |
| 2. Was the study design appropriate for the stated aim(s)? | | | | | | | | | Yes | | | | | | | | | | | | | | | No | | | | | | | | | | | | | Do not know/ comment | | | | | | | | | | | | |
| 3. Was the sample size justified? | | | | | | | | | Yes | | | | | | | | | | | | | | | No | | | | | | | | | | | | | Do not know/ comment | | | | | | | | | | | | |
| 4. Was the target/reference population clearly defined? (Is it clear who the research was about?) | | | | | | | | | Yes | | | | | | | | | | | | | | | No | | | | | | | | | | | | | Do not know/ comment | | | | | | | | | | | | |
| 5. Was the sample frame taken from an appropriate population base so that it closely represented the target/reference population under investigation? | | | | | | | | | Yes | | | | | | | | | | | | | | | No | | | | | | | | | | | | | Do not know/ comment | | | | | | | | | | | | |
| 6. Was the selection process likely to select subjects/participants that were representative of the target/reference population under investigation? | | | | | | | | | Yes | | | | | | | | | | | | | | | No | | | | | | | | | | | | | Do not know/ comment | | | | | | | | | | | | |
| 7. Were measures undertaken to address and categorise non-responders? | | | | | | | | | Yes | | | | | | | | | | | | | | | No | | | | | | | | | | | | | Do not know/ comment | | | | | | | | | | | | |
| 8. Were the risk factor and outcome variables measured appropriate to the aims of the study? | | | | | | | | | Yes | | | | | | | | | | | | | | | No | | | | | | | | | | | | | Do not know/ comment | | | | | | | | | | | | |
| 9. Were the risk factor and outcome variables measured correctly using instruments/ measurements that had been trialled, piloted or published previously? | | | | | | | | | Yes | | | | | | | | | | | | | | | No | | | | | | | | | | | | | Do not know/ comment | | | | | | | | | | | | |
| 10. Is it clear what was used to determined statistical significance and/or precision estimates? (eg, p values, CIs) | | | | | | | | | Yes | | | | | | | | | | | | | | | No | | | | | | | | | | | | | Do not know/ comment | | | | | | | | | | | | |
| 11. Were the methods (including statistical methods) sufficiently described to enable them to be repeated? | | | | | | | | | Yes | | | | | | | | | | | | | | | No | | | | | | | | | | | | | Do not know/ comment | | | | | | | | | | | | |
| Results | | | | | | | | | | | | | | | | | | | | | | | | | | | | | | | | | | | | | | | | | | | | | | | | | |
| 12. Were the basic data adequately described? | | | | | | | | | Yes | | | | | | | | | | | | | | | No | | | | | | | | | | | | | Do not know/ comment | | | | | | | | | | | | |
| 13. Does the response rate raise concerns about non-response bias? | | | | | | | | | Yes | | | | | | | | | | | | | | | No | | | | | | | | | | | | | Do not know/ comment | | | | | | | | | | | | |
| 14. If appropriate, was information about non-responders described? | | | | | | | | | Yes | | | | | | | | | | | | | | | No | | | | | | | | | | | | | Do not know/ comment | | | | | | | | | | | | |
| 15. Were the results internally consistent? | | | | | | | | | Yes | | | | | | | | | | | | | | | No | | | | | | | | | | | | | Do not know/ comment | | | | | | | | | | | | |
| 16. Were the results for the analyses described in the methods, presented? | | | | | | | | | Yes | | | | | | | | | | | | | | | No | | | | | | | | | | | | | Do not know/ comment | | | | | | | | | | | | |
|  | | | | | | | | | | | | | | | | | | | | | | | | | | | | | | | | | | | | | | | | | | | | | | | | | |
| **M. The Joanna Briggs Institute (JBI) Critical Appraisal Checklist for studies reporting prevalence data (last amended in 2017)**  **Website:** https://joannabriggs.org/critical_appraisal_tools  https://wiki.joannabriggs.org/display/MANUAL/Appendix+5.1%3A+Critical+Appraisal+Instrument+for+Studies+Reporting+Prevalence+Data | | | | | | | | | | | | | | | | | | | | | | | | | | | | | | | | | | | | | | | | | | | | | | | | | |
| Major Components | | | | | | | | | | | Response options | | | | | | | | | | | | | | | | | | | | | | | | | | | | | | | | | | | | | | |
| 1. Was the sample frame appropriate to address the target population? | | | | | | | | | | | Yes | | | | | | | | | | | | No | | | | | | | | | Unclear | | | | | | | | | | | | | Not applicable | | | | |
| 2. Were study participants sampled in an appropriate way? | | | | | | | | | | | Yes | | | | | | | | | | | | No | | | | | | | | | Unclear | | | | | | | | | | | | | Not applicable | | | | |
| 3. Was the sample size adequate? | | | | | | | | | | | Yes | | | | | | | | | | | | No | | | | | | | | | Unclear | | | | | | | | | | | | | Not applicable | | | | |
| 4. Were the study subjects and the setting described in detail? | | | | | | | | | | | Yes | | | | | | | | | | | | No | | | | | | | | | Unclear | | | | | | | | | | | | | Not applicable | | | | |
| 5. Was the data analysis conducted with sufficient coverage of the identified sample? | | | | | | | | | | | Yes | | | | | | | | | | | | No | | | | | | | | | Unclear | | | | | | | | | | | | | Not applicable | | | | |
| 6. Were valid methods used for the identification of the condition? | | | | | | | | | | | Yes | | | | | | | | | | | | No | | | | | | | | | Unclear | | | | | | | | | | | | | Not applicable | | | | |
| 7. Was the condition measured in a standard, reliable way for all participants? | | | | | | | | | | | Yes | | | | | | | | | | | | No | | | | | | | | | Unclear | | | | | | | | | | | | | Not applicable | | | | |
| 8. Was there appropriate statistical analysis? | | | | | | | | | | | Yes | | | | | | | | | | | | No | | | | | | | | | Unclear | | | | | | | | | | | | | Not applicable | | | | |
| 9. Was the response rate adequate, and if not, was the low response rate managed appropriately? | | | | | | | | | | | Yes | | | | | | | | | | | | No | | | | | | | | | Unclear | | | | | | | | | | | | | Not applicable | | | | |
| Overall appraisal: Include □ Exclude □ Seek further info □ | | | | | | | | | | | | | | | | | | | | | | | | | | | | | | | | | | | | | | | | | | | | | | | | | |
|  | | | | | | | | | | | | | | | | | | | | | | | | | | | | | | | | | | | | | | | | | | | | | | | | | |
| **N. The Agency for Healthcare Research and Quality (AHRQ) Methodology Checklist for Cross-Sectional/Prevalence Study**  **Website:** http://www.ncbi.nlm.nih.gov/books/NBK35156/ | | | | | | | | | | | | | | | | | | | | | | | | | | | | | | | | | | | | | | | | | | | | | | | | | |
| Major Components | | | | | | | | | | | | | | | | | | | | | | | | | | | | | | | | Response options | | | | | | | | | | | | | | | | | |
| 1. Define the source of information (survey, record review) | | | | | | | | | | | | | | | | | | | | | | | | | | | | | | | | Yes | | | | | | | No | | | | | | | | | | Unclear |
| 2. List inclusion and exclusion criteria for exposed and unexposed subjects (cases and controls) or refer to previous publications | | | | | | | | | | | | | | | | | | | | | | | | | | | | | | | | Yes | | | | | | | No | | | | | | | | | | Unclear |
| 3. Indicate time period used for identifying patients | | | | | | | | | | | | | | | | | | | | | | | | | | | | | | | | Yes | | | | | | | No | | | | | | | | | | Unclear |
| 4. Indicate whether or not subjects were consecutive if not population-based | | | | | | | | | | | | | | | | | | | | | | | | | | | | | | | | Yes | | | | | | | No | | | | | | | | | | Unclear |
| 5. Indicate if evaluators of subjective components of study were masked to other aspects of the status of the participants | | | | | | | | | | | | | | | | | | | | | | | | | | | | | | | | Yes | | | | | | | No | | | | | | | | | | Unclear |
| 6. Describe any assessments undertaken for quality assurance purposes (e.g., test/retest of primary outcome measurements) | | | | | | | | | | | | | | | | | | | | | | | | | | | | | | | | Yes | | | | | | | No | | | | | | | | | | Unclear |
| 7. Explain any patient exclusions from analysis | | | | | | | | | | | | | | | | | | | | | | | | | | | | | | | | Yes | | | | | | | No | | | | | | | | | | Unclear |
| 8. Describe how confounding was assessed and/or controlled | | | | | | | | | | | | | | | | | | | | | | | | | | | | | | | | Yes | | | | | | | No | | | | | | | | | | Unclear |
| 9. If applicable, explain how missing data were handled in the analysis | | | | | | | | | | | | | | | | | | | | | | | | | | | | | | | | Yes | | | | | | | No | | | | | | | | | | Unclear |
| 10. Summarize patient response rates and completeness of data collection | | | | | | | | | | | | | | | | | | | | | | | | | | | | | | | | Yes | | | | | | | No | | | | | | | | | | Unclear |
| 11. Clarify what follow-up, if any, was expected and the percentage of patients for which incomplete data or follow-up was obtained | | | | | | | | | | | | | | | | | | | | | | | | | | | | | | | | Yes | | | | | | | No | | | | | | | | | | Unclear |
|  | | | | | | | | | | | | | | | | | | | | | | | | | | | | | | | | | | | | | | | | | | | | | | | | | |
| O. **Crombie’s items^1^**  ^1^ Can be modified by user | | | | | | | | | | | | | | | | | | | | | | | | | | | | | | | | | | | | | | | | | | | | | | | | | |
| Major Components | | | | | Response options | | | | | | | | | | | | | | | | | | | | | | | | | | | | | | | | | | | | | | | | | | | | |
| 1. Appropriateness of design to meet the aims | | | | | Yes (1 point) | | | | | | | | | | | | | | | Unclear (0.5 point) | | | | | | | | | | | | | | | | | | | | | | No (0 point) | | | | | | | |
| 2. Adequate description of the data | | | | | Yes (1 point) | | | | | | | | | | | | | | | Unclear (0.5 point) | | | | | | | | | | | | | | | | | | | | | | No (0 point) | | | | | | | |
| 3. Report the response rates | | | | | Yes (1 point) | | | | | | | | | | | | | | | Unclear (0.5 point) | | | | | | | | | | | | | | | | | | | | | | No (0 point) | | | | | | | |
| 4. Adequate representativeness of the sample to total | | | | | Yes (1 point) | | | | | | | | | | | | | | | Unclear (0.5 point) | | | | | | | | | | | | | | | | | | | | | | No (0 point) | | | | | | | |
| 5. Clearly stated aims and likelihood of reliable and valid measurements | | | | | Yes (1 point) | | | | | | | | | | | | | | | Unclear (0.5 point) | | | | | | | | | | | | | | | | | | | | | | No (0 point) | | | | | | | |
| 6. Assessment of statistical significance | | | | | Yes (1 point) | | | | | | | | | | | | | | | Unclear (0.5 point) | | | | | | | | | | | | | | | | | | | | | | No (0 point) | | | | | | | |
| 7. Adequate description of statistical methods | | | | | Yes (1 point) | | | | | | | | | | | | | | | Unclear (0.5 point) | | | | | | | | | | | | | | | | | | | | | | No (0 point) | | | | | | | |
|  | | | | | | | | | | | | | | | | | | | | | | | | | | | | | | | | | | | | | | | | | | | | | | | | | |
| **P. The Joanna Briggs Institute (JBI) Critical Appraisal Checklist for Case Reports (last amended in 2017)**  **Website:** https://joannabriggs.org/critical_appraisal_tools  https://wiki.joannabriggs.org/display/MANUAL/Appendix+7.4+Critical+appraisal+checklist+for+case+reports | | | | | | | | | | | | | | | | | | | | | | | | | | | | | | | | | | | | | | | | | | | | | | | | | |
| Major Components | | | | | | | | | Response options | | | | | | | | | | | | | | | | | | | | | | | | | | | | | | | | | | | | | | | | |
| 1. Were patient’s demographic characteristics clearly described? | | | | | | | | | Yes | | | | | | | | | | | No | | | | | | | | | | | | Unclear | | | | | | | | | | Not applicable | | | | | | | |
| 2. Was the patient’s history clearly described and presented as a timeline? | | | | | | | | | Yes | | | | | | | | | | | No | | | | | | | | | | | | Unclear | | | | | | | | | | Not applicable | | | | | | | |
| 3. Was the current clinical condition of the patient on presentation clearly described? | | | | | | | | | Yes | | | | | | | | | | | No | | | | | | | | | | | | Unclear | | | | | | | | | | Not applicable | | | | | | | |
| 4. Were diagnostic tests or assessment methods and the results clearly described? | | | | | | | | | Yes | | | | | | | | | | | No | | | | | | | | | | | | Unclear | | | | | | | | | | Not applicable | | | | | | | |
| 5. Was the intervention(s) or treatment procedure(s) clearly described? | | | | | | | | | Yes | | | | | | | | | | | No | | | | | | | | | | | | Unclear | | | | | | | | | | Not applicable | | | | | | | |
| 6. Was the post-intervention clinical condition clearly described? | | | | | | | | | Yes | | | | | | | | | | | No | | | | | | | | | | | | Unclear | | | | | | | | | | Not applicable | | | | | | | |
| 7. Were adverse events (harms) or unanticipated events identified and described? | | | | | | | | | Yes | | | | | | | | | | | No | | | | | | | | | | | | Unclear | | | | | | | | | | Not applicable | | | | | | | |
| 8. Does the case report provide takeaway lessons? | | | | | | | | | Yes | | | | | | | | | | | No | | | | | | | | | | | | Unclear | | | | | | | | | | Not applicable | | | | | | | |
| Overall appraisal: Include □ Exclude □ Seek further info □ | | | | | | | | | | | | | | | | | | | | | | | | | | | | | | | | | | | | | | | | | | | | | | | | | |
|  | | | | | | | | | | | | | | | | | | | | | | | | | | | | | | | | | | | | | | | | | | | | | | | | | |
| **Q. The Good Research for Comparative Effectiveness (GRACE) Checklist v5.0 (last amended in 2016)**  **Website:** https://www.graceprinciples.org/ | | | | | | | | | | | | | | | | | | | | | | | | | | | | | | | | | | | | | | | | | | | | | | | | | |
| Major Components | Response options | | | | | | | | | | | | | | | | | | | | | | | | | | | | | | | | | | | | | | | | | | | | | | | | |
| Data | | | | | | | | | | | | | | | | | | | | | | | | | | | | | | | | | | | | | | | | | | | | | | | | | |
| D1. Were treatment and/or important details of treatment exposure adequately recorded for the study purpose in the data source(s)? | Yes | | | | | | | | | No | | | | | | | | | | | | | | | | | Not enough information | | | | | | | | | | | | | | / | | | | | | | | |
| D2. Were the primary outcomes adequately recorded for the study purpose (e.g., available in sufficient detail through data source(s))? | Yes | | | | | | | | | No | | | | | | | | | | | | | | | | | Not enough information | | | | | | | | | | | | | | / | | | | | | | | |
| D3. Was the primary clinical outcome(s) measured objectively rather than subject to clinical judgment (e.g., opinion about whether the patient’s condition has improved)? | Yes | | | | | | | | | No | | | | | | | | | | | | | | | | | Not enough information | | | | | | | | | | | | | | N/A | | | | | | | | |
| D4. Were primary outcomes validated, adjudicated, or otherwise known to be valid in a similar population? | Yes | | | | | | | | | No | | | | | | | | | | | | | | | | | Not enough information | | | | | | | | | | | | | | / | | | | | | | | |
| D5. Was the primary outcome(s) measured or identified in an equivalent manner between the treatment/ intervention group and the comparison group(s)? | Yes | | | | | | | | | No | | | | | | | | | | | | | | | | | Not enough information | | | | | | | | | | | | | | / | | | | | | | | |
| D6. Were important covariates that may be known confounders or effect modifiers available and recorded? | Yes | | | | | | | | | No | | | | | | | | | | | | | | | | | Not enough information | | | | | | | | | | | | | | / | | | | | | | | |
| Methods | | | | | | | | | | | | | | | | | | | | | | | | | | | | | | | | | | | | | | | | | | | | | | | | | |
| M1. Was the study (or analysis) population restricted to new initiators of treatment or those starting a new course of treatment? | Yes | | | | | | | | | No | | | | | | | | | | | | | | | | | Not enough information | | | | | | | | | | | | | | / | | | | | | | | |
| M2. If one or more comparison groups were used, were they concurrent comparators? If not, did the authors justify the use of historical comparisons group(s)? | Yes | | | | | | | | | No | | | | | | | | | | | | | | | | | Not enough information | | | | | | | | | | | | | | / | | | | | | | | |
| M3. Were important covariates, confounding and effect modifying variables taken into account in the design and/or analysis? | Yes | | | | | | | | | No | | | | | | | | | | | | | | | | | Not enough information | | | | | | | | | | | | | | At least one important covariate was not measured | | | | | | | | |
| M4. Is the classification of exposed and unexposed person-time free of “immortal time bias”? | Yes | | | | | | | | | No | | | | | | | | | | | | | | | | | Not enough information | | | | | | | | | | | | | | / | | | | | | | | |
| M5. Were any meaningful analyses conducted to test key assumptions on which primary results are based? | Yes (Primary results did not substantially change) | | | | | | | | | Yes (Primary results changed substantially) | | | | | | | | | | | | | | | | | Not enough information | | | | | | | | | | | | | | None reported | | | | | | | | |
|  | | | | | | | | | | | | | | | | | | | | | | | | | | | | | | | | | | | | | | | | | | | | | | | | | |
| **R. The Quality Assessment of Diagnostic Accuracy Studies (QUADAS)-2 tool**  **Website:** http://www.bristol.ac.uk/population-health-sciences/projects/quadas/ | | | | | | | | | | | | | | | | | | | | | | | | | | | | | | | | | | | | | | | | | | | | | | | | | |
| Major Components | | | | | | | | | | | | | | | | | Response options | | | | | | | | | | | | | | | | | | | | | | | | | | | | | | | | |
| **Patient Selection** | | | | | | | | | | | | | | | | | | | | | | | | | | | | | | | | | | | | | | | | | | | | | | | | | |
| 1. Was a consecutive or random sample of patients enrolled? | | | | | | | | | | | | | | | | | Yes | | | | | | | | | | | | | | | | No | | | | | | | | | | Unclear | | | | | | |
| 2. Was a case-control design avoided? | | | | | | | | | | | | | | | | | Yes | | | | | | | | | | | | | | | | No | | | | | | | | | | Unclear | | | | | | |
| 3. Did the study avoid inappropriate exclusions? | | | | | | | | | | | | | | | | | Yes | | | | | | | | | | | | | | | | No | | | | | | | | | | Unclear | | | | | | |
| 4. Could the selection of patients have introduced bias? | | | | | | | | | | | | | | | | | High | | | | | | | | | | | | | | | | Low | | | | | | | | | | Unclear | | | | | | |
| 5. Are there concerns that the included patients do not match the review question? | | | | | | | | | | | | | | | | | High | | | | | | | | | | | | | | | | Low | | | | | | | | | | Unclear | | | | | | |
| **Index Test** | | | | | | | | | | | | | | | | | | | | | | | | | | | | | | | | | | | | | | | | | | | | | | | | | |
| 6. Were the index test results interpreted without knowledge of the results of the reference standard? | | | | | | | | | | | | | | | | | Yes | | | | | | | | | | | | | | | | No | | | | | | | | | | Unclear | | | | | | |
| 7. If a threshold was used, was it pre-specified? | | | | | | | | | | | | | | | | | Yes | | | | | | | | | | | | | | | | No | | | | | | | | | | Unclear | | | | | | |
| 8. Could the conduct or interpretation of the index test have introduced bias? | | | | | | | | | | | | | | | | | High | | | | | | | | | | | | | | | | Low | | | | | | | | | | Unclear | | | | | | |
| 9. Are there concerns that the index test, its conduct, or interpretation differ from the review question? | | | | | | | | | | | | | | | | | High | | | | | | | | | | | | | | | | Low | | | | | | | | | | Unclear | | | | | | |
| **Reference Standard** | | | | | | | | | | | | | | | | | | | | | | | | | | | | | | | | | | | | | | | | | | | | | | | | | |
| 10. Is the reference standard likely to correctly classify the target condition? | | | | | | | | | | | | | | | | | Yes | | | | | | | | | | | | | | | | No | | | | | | | | | | Unclear | | | | | | |
| 11. Were the reference standard results interpreted without knowledge of the results of the index test? | | | | | | | | | | | | | | | | | Yes | | | | | | | | | | | | | | | | No | | | | | | | | | | Unclear | | | | | | |
| 12. Could the reference standard, its conduct, or its interpretation have introduced bias? | | | | | | | | | | | | | | | | | High | | | | | | | | | | | | | | | | Low | | | | | | | | | | Unclear | | | | | | |
| 13. Are there concerns that the target condition as defined by the reference standard does not match the review question? | | | | | | | | | | | | | | | | | High | | | | | | | | | | | | | | | | Low | | | | | | | | | | Unclear | | | | | | |
| **Flow and Timing** | | | | | | | | | | | | | | | | | | | | | | | | | | | | | | | | | | | | | | | | | | | | | | | | | |
| 14. Was there an appropriate interval between index test(s) and reference standard? | | | | | | | | | | | | | | | | | Yes | | | | | | | | | | | | | | | | No | | | | | | | | | | Unclear | | | | | | |
| 15. Did all patients receive a reference standard? | | | | | | | | | | | | | | | | | Yes | | | | | | | | | | | | | | | | No | | | | | | | | | | Unclear | | | | | | |
| 16. Did all patients receive the same reference standard? | | | | | | | | | | | | | | | | | Yes | | | | | | | | | | | | | | | | No | | | | | | | | | | Unclear | | | | | | |
| 17. Were all patients included in the analysis? | | | | | | | | | | | | | | | | | Yes | | | | | | | | | | | | | | | | No | | | | | | | | | | Unclear | | | | | | |
| 18. Could the patient flow have introduced bias? | | | | | | | | | | | | | | | | | High | | | | | | | | | | | | | | | | Low | | | | | | | | | | Unclear | | | | | | |
|  | | | | | | | | | | | | | | | | | | | | | | | | | | | | | | | | | | | | | | | | | | | | | | | | | |
| **S. The Critical Appraisal Skills Programme (CASP) Checklist for diagnostic test study (last amended in 2018)**  **Website:** https://casp-uk.net/casp-tools-checklists/ | | | | | | | | | | | | | | | | | | | | | | | | | | | | | | | | | | | | | | | | | | | | | | | | | |
| Major Components | | | | | | | | | Response options | | | | | | | | | | | | | | | | | | | | | | | | | | | | | | | | | | | | | | | | |
| **Section A: Are the results of the trial valid?** | | | | | | | | | | | | | | | | | | | | | | | | | | | | | | | | | | | | | | | | | | | | | | | | | |
| 1. Was there a clear question for the study to address? | | | | | | | | | Yes | | | | | | | | | | | | | | | No | | | | | | | | | | | | | Can’t Tell | | | | | | | | | | | | |
| 2. Was there a comparison with an appropriate reference standard? | | | | | | | | | Yes | | | | | | | | | | | | | | | No | | | | | | | | | | | | | Can’t Tell | | | | | | | | | | | | |
| Is it worth continuing? | | | | | | | | | Yes | | | | | | | | | | | | | | | No | | | | | | | | | | | | | Can’t Tell | | | | | | | | | | | | |
| 3. Did all patients get the diagnostic test and reference standard? | | | | | | | | | Yes | | | | | | | | | | | | | | | No | | | | | | | | | | | | | Can’t Tell | | | | | | | | | | | | |
| 4. Could the results of the test have been influenced by the results of the reference standard? | | | | | | | | | Yes | | | | | | | | | | | | | | | No | | | | | | | | | | | | | Can’t Tell | | | | | | | | | | | | |
| 5. Is the disease status of the tested population clearly described? | | | | | | | | | Yes | | | | | | | | | | | | | | | No | | | | | | | | | | | | | Can’t Tell | | | | | | | | | | | | |
| 6. Were the methods for performing the test described in sufficient detail? | | | | | | | | | Yes | | | | | | | | | | | | | | | No | | | | | | | | | | | | | Can’t Tell | | | | | | | | | | | | |
| **Section B: What are the results?** | | | | | | | | | | | | | | | | | | | | | | | | | | | | | | | | | | | | | | | | | | | | | | | | | |
| 7. What are the results? | | | | | | | | |  | | | | | | | | | | | | | | | | | | | | | | | | | | | | | | | | | | | | | | | | |
| 8. How sure are we about the results? Consequences and cost of alternatives performed? | | | | | | | | |  | | | | | | | | | | | | | | | | | | | | | | | | | | | | | | | | | | | | | | | | |
| **Section C: Will the results help locally?**  Consider whether you are primarily interested in the impact on a population or individual level | | | | | | | | | | | | | | | | | | | | | | | | | | | | | | | | | | | | | | | | | | | | | | | | | |
| 9. Can the results be applied to your patients/the population of interest? | | | | | | | | | Yes | | | | | | | | | | | | | | | No | | | | | | | | | | | | | Can’t Tell | | | | | | | | | | | | |
| 10. Can the test be applied to your patient or population of interest? | | | | | | | | | Yes | | | | | | | | | | | | | | | No | | | | | | | | | | | | | Can’t Tell | | | | | | | | | | | | |
| 11. Were all outcomes important to the individual or population considered? | | | | | | | | | Yes | | | | | | | | | | | | | | | No | | | | | | | | | | | | | Can’t Tell | | | | | | | | | | | | |
| 12. What would be the impact of using this test on your patients/population? | | | | | | | | |  | | | | | | | | | | | | | | | | | | | | | | | | | | | | | | | | | | | | | | | | |
|  | | | | | | | | | | | | | | | | | | | | | | | | | | | | | | | | | | | | | | | | | | | | | | | | | |
| **T. The Scottish Intercollegiate Guidelines Network (SIGN) Methodology checklist: diagnostic studies (last amended in 2014)**  **Website:** https://www.sign.ac.uk/checklists-and-notes.html | | | | | | | | | | | | | | | | | | | | | | | | | | | | | | | | | | | | | | | | | | | | | | | | | |
| Major Components | | Response options | | | | | | | | | | | | | | | | | | | | | | | | | | | | | | | | | | | | | | | | | | | | | | | |
| **DOMAIN 1 – PATIENT SELECTION** | | | | | | | | | | | | | | | | | | | | | | | | | | | | | | | | | | | | | | | | | | | | | | | | | |
| Risk of bias | | | | | | | | | | | | | | | | | | | | | | | | | | | | | | | | | | | | | | | | | | | | | | | | | |
| In a well conducted diagnostic study… | | Is that true in this study? | | | | | | | | | | | | | | | | | | | | | | | | | | | | | | | | | | | | | | | | | | | | | | | |
| 1.1. A consecutive sequence or random selection of patients is enrolled. | | Yes | | | | | | | | | | | | No | | | | | | | | | | | | | | | | | | | | | Can’t say | | | | | | | | | | | | | | |
| 1.2. Case – control methods are not used. | | Yes | | | | | | | | | | | | No | | | | | | | | | | | | | | | | | | | | | Can’t say | | | | | | | | | | | | | | |
| 1.3. Inappropriate exclusions are avoided. | | Yes | | | | | | | | | | | | No | | | | | | | | | | | | | | | | | | | | | Can’t say | | | | | | | | | | | | | | |
| Applicability | | | | | | | | | | | | | | | | | | | | | | | | | | | | | | | | | | | | | | | | | | | | | | | | | |
| 1.4. The included patients and settings match the key question. | | Yes | | | | | | | | | | | | No | | | | | | | | | | | | | | | | | | | | | Can’t say | | | | | | | | | | | | | | |
| **DOMAIN 2 – INDEX TEST** | | | | | | | | | | | | | | | | | | | | | | | | | | | | | | | | | | | | | | | | | | | | | | | | | |
| Risk of bias | | | | | | | | | | | | | | | | | | | | | | | | | | | | | | | | | | | | | | | | | | | | | | | | | |
| In a well conducted diagnostic study… | | Is that true in this study? | | | | | | | | | | | | | | | | | | | | | | | | | | | | | | | | | | | | | | | | | | | | | | | |
| 2.1. The index test results interpreted without knowledge of the results of the reference standard. | | Yes | | | | | | | | | | | | No | | | | | | | | | | | | | | | | | | | | | Can’t say | | | | | | | | | | | | | | |
| 2.2. If a threshold is used, it is pre-specified. | | Yes | | | | | | | | | | | | No | | | | | | | | | | | | | | | | | | | | | Can’t say | | | | | | | | | | | | | | |
| Applicability | | | | | | | | | | | | | | | | | | | | | | | | | | | | | | | | | | | | | | | | | | | | | | | | | |
| 2.3. The index test, its conduct, and its interpretation is similar to that used in practice with the target population of the guideline. | | Yes | | | | | | | | | | | | No | | | | | | | | | | | | | | | | | | | | | Can’t say | | | | | | | | | | | | | | |
| **DOMAIN 3 – REFERENCE STANDARD** | | | | | | | | | | | | | | | | | | | | | | | | | | | | | | | | | | | | | | | | | | | | | | | | | |
| Risk of bias | | | | | | | | | | | | | | | | | | | | | | | | | | | | | | | | | | | | | | | | | | | | | | | | | |
| In a well conducted diagnostic study… | | Is that true in this study? | | | | | | | | | | | | | | | | | | | | | | | | | | | | | | | | | | | | | | | | | | | | | | | |
| 3.1. The reference standard is likely to correctly identify the target condition. | | Yes | | | | | | | | | | | | No | | | | | | | | | | | | | | | | | | | | | Can’t say | | | | | | | | | | | | | | |
| 3.2. Reference standard results are interpreted without knowledge of the results of the index test. | | Yes | | | | | | | | | | | | No | | | | | | | | | | | | | | | | | | | | | Can’t say | | | | | | | | | | | | | | |
| Applicability | | | | | | | | | | | | | | | | | | | | | | | | | | | | | | | | | | | | | | | | | | | | | | | | | |
| 3.3. The target condition as defined by the reference standard matches that found in the target population of the guideline. | | Yes | | | | | | | | | | | | No | | | | | | | | | | | | | | | | | | | | | Can’t say | | | | | | | | | | | | | | |
| **DOMAIN 4 – FLOW AND TIMING** | | | | | | | | | | | | | | | | | | | | | | | | | | | | | | | | | | | | | | | | | | | | | | | | | |
| Risk of bias | | | | | | | | | | | | | | | | | | | | | | | | | | | | | | | | | | | | | | | | | | | | | | | | | |
| In a well conducted diagnostic study… | | Is that true in this study? | | | | | | | | | | | | | | | | | | | | | | | | | | | | | | | | | | | | | | | | | | | | | | | |
| 4.1. There is an appropriate interval between the index test and reference standard. | | Yes | | | | | | | | | | | | No | | | | | | | | | | | | | | | | | | | | | Can’t say | | | | | | | | | | | | | | |
| 4.2. All patients receive the same reference standard. | | Yes | | | | | | | | | | | | No | | | | | | | | | | | | | | | | | | | | | Can’t say | | | | | | | | | | | | | | |
| 4.3. All patients recruited into the study are included in the analysis. | | Yes | | | | | | | | | | | | No | | | | | | | | | | | | | | | | | | | | | Can’t say | | | | | | | | | | | | | | |
| **SECTION 5: OVERALL ASSESSMENT OF THE STUDY** | | | | | | | | | | | | | | | | | | | | | | | | | | | | | | | | | | | | | | | | | | | | | | | | | |
| 5.1. How well was the study done to minimise bias? Code as follows: | | High quality (++) | | | | | | | | | | | | Acceptable (+) | | | | | | | | | | | | | | | | | | | | | Unacceptable – reject 0 | | | | | | | | | | | | | | |
| 5.2. What is your assessment of the applicability of this study to our target population? | | Directly applicable | | | | | | | | | | | | Some indirectness (Please explain in the following section for Notes) | | | | | | | | | | | | | | | | | | | | | | | | | | | | | | | | | | | |
| 5.2. Notes. Summarise the authors conclusions. Add any comments on your own assessment of the study, and the extent to which it answers your question. | | | | | | | | | | | | | | | | | | | | | | | | | | | | | | | | | | | | | | | | | | | | | | | | | |
|  | | | | | | | | | | | | | | | | | | | | | | | | | | | | | | | | | | | | | | | | | | | | | | | | | |
| **U. The Joanna Briggs Institute (JBI) Critical Appraisal Checklist for diagnostic test accuracy studies (last amended in 2017)**  **Website:** https://joannabriggs.org/critical_appraisal_tools  https://wiki.joannabriggs.org/display/MANUAL/Appendix+9.1+Critical+appraisal+checklist | | | | | | | | | | | | | | | | | | | | | | | | | | | | | | | | | | | | | | | | | | | | | | | | | |
| Major Components | | | | | | | | | Response options | | | | | | | | | | | | | | | | | | | | | | | | | | | | | | | | | | | | | | | | |
| 1. Was a consecutive or random sample of patients enrolled? | | | | | | | | | Yes | | | | | | | | | | | | No | | | | | | | | | Unclear | | | | | | | | | | | Not applicable | | | | | | | | |
| 2. Was a case-control design avoided? | | | | | | | | | Yes | | | | | | | | | | | | No | | | | | | | | | Unclear | | | | | | | | | | | Not applicable | | | | | | | | |
| 3. Did the study avoid inappropriate exclusions? | | | | | | | | | Yes | | | | | | | | | | | | No | | | | | | | | | Unclear | | | | | | | | | | | Not applicable | | | | | | | | |
| 4. Were the index test results interpreted without knowledge of the results of the reference standard? | | | | | | | | | Yes | | | | | | | | | | | | No | | | | | | | | | Unclear | | | | | | | | | | | Not applicable | | | | | | | | |
| 5. If a threshold was used, was it pre-specified? | | | | | | | | | Yes | | | | | | | | | | | | No | | | | | | | | | Unclear | | | | | | | | | | | Not applicable | | | | | | | | |
| 6. Is the reference standard likely to correctly classify the target condition? | | | | | | | | | Yes | | | | | | | | | | | | No | | | | | | | | | Unclear | | | | | | | | | | | Not applicable | | | | | | | | |
| 7. Were the reference standard results interpreted without knowledge of the results of the index test? | | | | | | | | | Yes | | | | | | | | | | | | No | | | | | | | | | Unclear | | | | | | | | | | | Not applicable | | | | | | | | |
| 8. Was there an appropriate interval between the index test and the reference standard? | | | | | | | | | Yes | | | | | | | | | | | | No | | | | | | | | | Unclear | | | | | | | | | | | Not applicable | | | | | | | | |
| 9. Did all patients receive the same reference standard? | | | | | | | | | Yes | | | | | | | | | | | | No | | | | | | | | | Unclear | | | | | | | | | | | Not applicable | | | | | | | | |
| 10. Were all patients included in the analysis? | | | | | | | | | Yes | | | | | | | | | | | | No | | | | | | | | | Unclear | | | | | | | | | | | Not applicable | | | | | | | | |
| Overall appraisal: Include □ Exclude □ Seek further info □ | | | | | | | | | | | | | | | | | | | | | | | | | | | | | | | | | | | | | | | | | | | | | | | | | |
|  | | | | | | | | | | | | | | | | | | | | | | | | | | | | | | | | | | | | | | | | | | | | | | | | | |
| **V. The Cochrane risk of bias assessing tool for diagnostic test accuracy (last released in 2009)**  **Website:** https://methods.cochrane.org/sdt/handbook-dta-reviews | | | | | | | | | | | | | | | | | | | | | | | | | | | | | | | | | | | | | | | | | | | | | | | | | |
| Major Components | | | | | | | | | | | | | | | | | | | | | Response options | | | | | | | | | | | | | | | | | | | | | | | | | | | | |
| 1. Was the spectrum of patients representative of the patients who will receive the test in practice? (representative spectrum) | | | | | | | | | | | | | | | | | | | | | Yes | | | | | | | | | No | | | | | | | | | | | Unclear | | | | | | | | |
| 2. Is the reference standard likely to classify the target condition correctly? (acceptable reference standard) | | | | | | | | | | | | | | | | | | | | | Yes | | | | | | | | | No | | | | | | | | | | | Unclear | | | | | | | | |
| 3. Is the time period between reference standard and index test short enough to be reasonably sure that the target condition did not change between the two tests? (acceptable delay between tests) | | | | | | | | | | | | | | | | | | | | | Yes | | | | | | | | | No | | | | | | | | | | | Unclear | | | | | | | | |
| 4. Did the whole sample or a random selection of the sample, receive verification using the intended reference standard? (partial verification avoided) | | | | | | | | | | | | | | | | | | | | | Yes | | | | | | | | | No | | | | | | | | | | | Unclear | | | | | | | | |
| 5. Did patients receive the same reference standard irrespective of the index test result? (differential verification avoided) | | | | | | | | | | | | | | | | | | | | | Yes | | | | | | | | | No | | | | | | | | | | | Unclear | | | | | | | | |
| 6. Was the reference standard independent of the index test (i.e. the index test did not form part of the reference standard)? (incorporation avoided) | | | | | | | | | | | | | | | | | | | | | Yes | | | | | | | | | No | | | | | | | | | | | Unclear | | | | | | | | |
| 7. Were the reference standard results interpreted without knowledge of the results of the index test? (index test results blinded) | | | | | | | | | | | | | | | | | | | | | Yes | | | | | | | | | No | | | | | | | | | | | Unclear | | | | | | | | |
| 8. Were the index test results interpreted without knowledge of the results of the reference standard? (reference standard results blinded) | | | | | | | | | | | | | | | | | | | | | Yes | | | | | | | | | No | | | | | | | | | | | Unclear | | | | | | | | |
| 9. Were the same clinical data available when test results were interpreted as would be available when the test is used in practice? (relevant clinical information) | | | | | | | | | | | | | | | | | | | | | Yes | | | | | | | | | No | | | | | | | | | | | Unclear | | | | | | | | |
| 10. Were uninterpretable/ intermediate test results reported? (uninterpretable results reported) | | | | | | | | | | | | | | | | | | | | | Yes | | | | | | | | | No | | | | | | | | | | | Unclear | | | | | | | | |
| 11. Were withdrawals from the study explained? (withdrawals explained) | | | | | | | | | | | | | | | | | | | | | Yes | | | | | | | | | No | | | | | | | | | | | Unclear | | | | | | | | |
| 12. Were cut-off values established before the study was started? | | | | | | | | | | | | | | | | | | | | | Yes | | | | | | | | | No | | | | | | | | | | | Unclear | | | | | | | | |
| 13. Is the technology of the index test unchanged since the study was carried out? | | | | | | | | | | | | | | | | | | | | | Yes | | | | | | | | | No | | | | | | | | | | | Unclear | | | | | | | | |
| 14. Did the study provide a clear definition of what was considered to be a ‘positive’ result? | | | | | | | | | | | | | | | | | | | | | Yes | | | | | | | | | No | | | | | | | | | | | Unclear | | | | | | | | |
| 15. Had test operators had appropriate training? | | | | | | | | | | | | | | | | | | | | | Yes | | | | | | | | | No | | | | | | | | | | | Unclear | | | | | | | | |
| 16. Was treatment withheld until both the index test and reference standard were performed? | | | | | | | | | | | | | | | | | | | | | Yes | | | | | | | | | No | | | | | | | | | | | Unclear | | | | | | | | |
| 17. Were data on observer variation reported and within an acceptable range? | | | | | | | | | | | | | | | | | | | | | Yes | | | | | | | | | No | | | | | | | | | | | Unclear | | | | | | | | |
| 18. Were data on instrument variation reported and within an acceptable range? | | | | | | | | | | | | | | | | | | | | | Yes | | | | | | | | | No | | | | | | | | | | | Unclear | | | | | | | | |
| 19. Were objectives pre-specified? | | | | | | | | | | | | | | | | | | | | | Yes | | | | | | | | | No | | | | | | | | | | | Unclear | | | | | | | | |
| 20. Was the study free of commercial funding? | | | | | | | | | | | | | | | | | | | | | Yes | | | | | | | | | No | | | | | | | | | | | Unclear | | | | | | | | |
