# Supplementary material for: Methodological quality (risk of bias) assessment tools for primary and secondary medical studies: what are they and which is better?
Source: Mil Med Res. 2020 Feb 29;7:7. doi: 10.1186/s40779-020-00238-8 (PMC7049186; doi:10.1186/s40779-020-00238-8)
Supplement: Supplementary file 3 — Additional file 3: Table S3. Major components of the tools for assessing other primary medical studies [file 40779_2020_238_MOESM3_ESM.docx]

**Table S3. Major components of the tools for assessing other primary medical studies**

| **A. The Scottish Intercollegiate Guidelines Network (SIGN) Methodology checklist: ecnomic evaluations (last amended in 2014)**  **Website:** https://www.sign.ac.uk/checklists-and-notes.html | | | | | | | | | | | | | | | | | | | | | | | |
| --- | --- | --- | --- | --- | --- | --- | --- | --- | --- | --- | --- | --- | --- | --- | --- | --- | --- | --- | --- | --- | --- | --- | --- |
| Major Components | | | Response options | | | | | | | | | | | | | | | | | | | | |
| **SECTION 1: INTERNAL VALIDITY** | | | | | | | | | | | | | | | | | | | | | | | |
| In a well conducted economic study... | | | Does this study do it? | | | | | | | | | | | | | | | | | | | | |
| 1.1. The study addresses an appropriate and clearly focused question. | | | Yes | | | | No | | | | | Can’t say | | | | | | | / | | | | |
| 1.2. The economic importance of the question is clear. | | | Yes | | | | No | | | | | Can’t say | | | | | | | / | | | | |
| 1.3. The choice of study design is justified. | | | Yes | | | | No | | | | | Can’t say | | | | | | | / | | | | |
| 1.4. All costs that are relevant from the viewpoint of the study are included and are measured and valued appropriately. | | | Yes | | | | No | | | | | / | | | | | | | / | | | | |
| 1.5. The outcome measures used to answer the study question are relevant to that purpose and are measured and valued appropriately. | | | Yes | | | | No | | | | | Can’t say | | | | | | | Not applicable | | | | |
| 1.6. If discounting of future costs and outcomes is necessary, it been performed correctly | | | Yes | | | | No | | | | | Can’t say | | | | | | | Not applicable | | | | |
| 1.7. Assumptions are made explicit and a sensitivity analysis performed | | | Yes | | | | No | | | | | Can’t say | | | | | | | / | | | | |
| 1.8. The decision rule is made explicit and comparisons are made on the basis of incremental costs and outcomes. | | | Yes | | | | No | | | | | / | | | | | | | / | | | | |
| 1.9. The results provide information of relevance to policy makers. | | | Yes | | | | No | | | | | / | | | | | | | / | | | | |
| **SECTION 2: OVERALL ASSESSMENT OF THE STUDY** | | | | | | | | | | | | | | | | | | | | | | | |
| 2.1. How well was the study conducted? | | | High quality (++) | | | | | | | | Acceptable (+) | | | | | | | Unacceptable – reject 0 | | | | | |
| 2.2. Are the results of this study directly applicable to the patient group targeted by this guideline? | | | Yes | | | | | | | | No | | | | | | | | | | | | |
| 2.3. Notes. Summarise the author’s conclusions. Add any comments on your own assessment of the study, and the extent to which it answers your question and mention any areas of uncertainty raised above. | | | | | | | | | | | | | | | | | | | | | | | |
|  | | | | | | | | | | | | | | | | | | | | | | | |
| **B. The Critical Appraisal Skills Programme (CASP) Checklist for ecnomic evaluation (last amended in 2018)**  **Website:** https://casp-uk.net/casp-tools-checklists/ | | | | | | | | | | | | | | | | | | | | | | | |
| Major Components | | | | | | | | | | | | | Response options | | | | | | | | | | |
| **Section A: Is the economic evaluation valid?** | | | | | | | | | | | | | | | | | | | | | | | |
| 1. Was a well-defined question posed? | | | | | | | | | | | | | Yes | | | No | | | | | Can’t Tell | | |
| 2. Was a comprehensive description of the competing alternatives given? | | | | | | | | | | | | | Yes | | | No | | | | | Can’t Tell | | |
| Is it worth continuing? | | | | | | | | | | | | | | | | | | | | | | | |
| 3. Does the paper provide evidence that the programme would be effective? (i.e. would the programme do more good than harm?) | | | | | | | | | | | | | Yes | | | No | | | | | Can’t Tell | | |
| 4. Were the effects of the intervention identified, measured and valued appropriately? | | | | | | | | | | | | | Yes | | | No | | | | | Can’t Tell | | |
| **Section B: How were consequences and costs assessed and compared?** | | | | | | | | | | | | | | | | | | | | | | | |
| 5. Were all important and relevant resources required, and health outcome costs for each alternative identified, measured in appropriate units and valued credibly? | | | | | | | | | | | | | Yes | | | No | | | | | Can’t Tell | | |
| 6. Were costs and consequences adjusted for different times at which they occurred (discounting)? | | | | | | | | | | | | | Yes | | | No | | | | | Can’t Tell | | |
| 7. What were the results of the evaluation? | | | | | | | | | | | | | Yes | | | No | | | | | Can’t Tell | | |
| 8. Was an incremental analysis of the consequences and cost of alternatives performed? | | | | | | | | | | | | | Yes | | | No | | | | | Can’t Tell | | |
| 9. Was an adequate sensitivity analysis performed? | | | | | | | | | | | | | Yes | | | No | | | | | Can’t Tell | | |
| Section C: Will the results help in purchasing for local people? | | | | | | | | | | | | | | | | | | | | | | | |
| 10. Is the programme likely to be equally effective in your context or setting? | | | | | | | | | | | | | Yes | | | No | | | | | Can’t Tell | | |
| 11. Are the costs translatable to your setting? | | | | | | | | | | | | | Yes | | | No | | | | | Can’t Tell | | |
| 12. Is it worth doing in your setting? | | | | | | | | | | | | | Yes | | | No | | | | | Can’t Tell | | |
|  | | | | | | | | | | | | | | | | | | | | | | | |
| **C. The Joanna Briggs Institute (JBI) Critical Appraisal Checklist for economic evaluations (last amended in 2014)**  **Website:** https://joannabriggs.org/critical_appraisal_tools | | | | | | | | | | | | | | | | | | | | | | | |
| Major Components | | | | Response options | | | | | | | | | | | | | | | | | | | |
| 1. Is there a well-defined question/objective? | | | | Yes | | | | No | | | | Unclear | | | | | | Not applicable | | | | | |
| 2. Is there a comprehensive description of alternatives? | | | | Yes | | | | No | | | | Unclear | | | | | | Not applicable | | | | | |
| 3. Are all important and relevant costs and outcomes for each alternative identified? | | | | Yes | | | | No | | | | Unclear | | | | | | Not applicable | | | | | |
| 4. Has clinical effectiveness been established? | | | | Yes | | | | No | | | | Unclear | | | | | | Not applicable | | | | | |
| 5. Are costs and outcomes measured accurately? | | | | Yes | | | | No | | | | Unclear | | | | | | Not applicable | | | | | |
| 6. Are costs and outcomes valued credibly? | | | | Yes | | | | No | | | | Unclear | | | | | | Not applicable | | | | | |
| 7. Are costs and outcomes adjusted for differential timing? | | | | Yes | | | | No | | | | Unclear | | | | | | Not applicable | | | | | |
| 8. Is there any incremental analysis of costs and consequences? | | | | Yes | | | | No | | | | Unclear | | | | | | Not applicable | | | | | |
| 9. Were sensitivity analysis conducted to investigate uncertainty in estimates of costs or outcomes? | | | | Yes | | | | No | | | | Unclear | | | | | | Not applicable | | | | | |
| 10. Do study results include all issues of concern to users? | | | | Yes | | | | No | | | | Unclear | | | | | | Not applicable | | | | | |
| 11. Are the results generalizable to the setting of interest in the review? | | | | Yes | | | | No | | | | Unclear | | | | | | Not applicable | | | | | |
| Overall appraisal: Include □ Exclude □ Seek further info □ | | | | | | | | | | | | | | | | | | | | | | | |
|  | | | | | | | | | | | | | | | | | | | | | | | |
| **D. The National Institute for Clinical Excellence (NICE) methodology checklist for economic evaluation (last amended in February 2019)**  **Website:** https://www.nice.org.uk/process/pmg20/resources/appendix-h-pdf-2549710190 | | | | | | | | | | | | | | | | | | | | | | | |
| Major Components | | | | | | Response options | | | | | | | | | | | | | | | | | |
| **Section 1: Applicability** (relevance to specific review questions and the NICE reference case as described in section 7.5)  This checklist should be used first to filter out irrelevant studies. | | | | | | | | | | | | | | | | | | | | | | | |
| 1.1. Is the study population appropriate for the review question? | | | | | | Yes/ No | | | | Partly | | | | Unclear | | | | | Not applicable | | | | |
| 1.2. Are the interventions appropriate for the review question? | | | | | | Yes/ No | | | | Partly | | | | Unclear | | | | | Not applicable | | | | |
| 1.3. Is the system in which the study was conducted sufficiently similar to the current UK context? | | | | | | Yes/ No | | | | Partly | | | | Unclear | | | | | Not applicable | | | | |
| 1.4. Is the perspective for costs appropriate for the review question? | | | | | | Yes/ No | | | | Partly | | | | Unclear | | | | | Not applicable | | | | |
| 1.5. Is the perspective for outcomes appropriate for the review question? | | | | | | Yes/ No | | | | Partly | | | | Unclear | | | | | Not applicable | | | | |
| 1.6. Are all future costs and outcomes discounted appropriately? | | | | | | Yes/ No | | | | Partly | | | | Unclear | | | | | Not applicable | | | | |
| 1.7. Are QALYs, derived using NICE’s preferred methods, or an appropriate social care-related equivalent used as an outcome? If not, describe rationale and outcomes used in line with analytical perspectives taken (item 1.5 above). | | | | | | Yes/ No | | | | Partly | | | | Unclear | | | | | Not applicable | | | | |
| 1.8. **Overall judgement:**  Directly applicable/ partially applicable/ not applicable  There is no need to use section 2 of the checklist if the study is considered ‘not applicable’. | | | | | | | | | | | | | | | | | | | | | | | |
| Other comments: | | | | | | | | | | | | | | | | | | | | | | | |
| **Section 2: Study limitations** (the level of methodological quality)  This checklist should be used once it has been decided that the study is sufficiently applicable to the context of the guideline | | | | | | | | | | | | | | | | | | | | | | | |
| 2.1. Does the model structure adequately reflect the nature of the topic under evaluation? | | | | | | Yes/ No | | | | Partly | | | | Unclear | | | | | Not applicable | | | | |
| 2.2. Is the time horizon sufficiently long to reflect all important differences in costs and outcomes? | | | | | | Yes/ No | | | | Partly | | | | Unclear | | | | | Not applicable | | | | |
| 2.3. Are all important and relevant outcomes included? | | | | | | Yes/ No | | | | Partly | | | | Unclear | | | | | Not applicable | | | | |
| 2.4. Are the estimates of baseline outcomes from the best available source? | | | | | | Yes/ No | | | | Partly | | | | Unclear | | | | | Not applicable | | | | |
| 2.5. Are the estimates of relative intervention effects from the best available source? | | | | | | Yes/ No | | | | Partly | | | | Unclear | | | | | Not applicable | | | | |
| 2.6. Are all important and relevant costs included? | | | | | | Yes/ No | | | | Partly | | | | Unclear | | | | | Not applicable | | | | |
| 2.7. Are the estimates of resource use from the best available source? | | | | | | Yes/ No | | | | Partly | | | | Unclear | | | | | Not applicable | | | | |
| 2.8. Are the unit costs of resources from the best available source? | | | | | | Yes/ No | | | | Partly | | | | Unclear | | | | | Not applicable | | | | |
| 2.9. Is an appropriate incremental analysis presented or can it be calculated from the data? | | | | | | Yes/ No | | | | Partly | | | | Unclear | | | | | Not applicable | | | | |
| 2.10. Are all important parameters whose values are uncertain subjected to appropriate sensitivity analysis? | | | | | | Yes/ No | | | | Partly | | | | Unclear | | | | | Not applicable | | | | |
| 2.11. Has no potential financial conflict of interest been declared? | | | | | | Yes/ No | | | | Partly | | | | Unclear | | | | | Not applicable | | | | |
| 2.12 **Overall assessment:** Minor limitations/ potentially serious limitations/ very serious limitations | | | | | | | | | | | | | | | | | | | | | | | |
| Other comments: | | | | | | | | | | | | | | | | | | | | | | | |
|  | | | | | | | | | | | | | | | | | | | | | | | |
| **E. The Critical Appraisal Skills Programme (CASP) Checklist for qualitative research (last amended in 2018)**  **Website:** https://casp-uk.net/casp-tools-checklists/ | | | | | | | | | | | | | | | | | | | | | | | |
| Major Components | | | | | | Response options | | | | | | | | | | | | | | | | | |
| **Section A: Are the results valid?** | | | | | | | | | | | | | | | | | | | | | | | |
| 1. Was there a clearstatement of the aims ofthe research? | | | | | | Yes | | | | | No | | | | Can’t Tell | | | | | | | | |
| 2. Is a qualitativemethodologyappropriate? | | | | | | Yes | | | | | No | | | | Can’t Tell | | | | | | | | |
| Is it worth continuing? | | | | | | | | | | | | | | | | | | | | | | | |
| 3. Was the researchdesign appropriate toaddress the aims of theresearch? | | | | | | Yes | | | | | No | | | | Can’t Tell | | | | | | | | |
| 4. Was the recruitment strategy appropriate to the aims of the research? | | | | | | Yes | | | | | No | | | | Can’t Tell | | | | | | | | |
| 5. Was the data collected in a way that addressed the research issue? | | | | | | Yes | | | | | No | | | | Can’t Tell | | | | | | | | |
| 6. Has the relationship between researcher and participants been adequately considered? | | | | | | Yes | | | | | No | | | | Can’t Tell | | | | | | | | |
| **Section B: What are the results?** | | | | | | | | | | | | | | | | | | | | | | | |
| 7. Have ethical issues been taken into consideration? | | | | | | Yes | | | | | No | | | | Can’t Tell | | | | | | | | |
| 8. Was the data analysis sufficiently rigorous? | | | | | | Yes | | | | | No | | | | Can’t Tell | | | | | | | | |
| 9. Is there a clear statement of findings? | | | | | | Yes | | | | | No | | | | Can’t Tell | | | | | | | | |
| **Section C: Will the results help locally?** | | | | | | | | | | | | | | | | | | | | | | | |
| 10. How valuable is the research? | | | | | | Yes | | | | | No | | | | Can’t Tell | | | | | | | | |
|  | | | | | | | | | | | | | | | | | | | | | | | |
| **F. The Joanna Briggs Institute (JBI) Critical Appraisal Checklist for qualitative research (last amended in 2017)**  **Website:** https://joannabriggs.org/critical_appraisal_tools | | | | | | | | | | | | | | | | | | | | | | | |
| Major Components | | | | | | Response options | | | | | | | | | | | | | | | | | |
| 1. Is there congruity between the stated philosophical perspective and the research methodology? | | | | | | Yes | | | | No | | | | Unclear | | | | | Not applicable | | | | |
| 2. Is there congruity between the research methodology and the research question or objectives? | | | | | | Yes | | | | No | | | | Unclear | | | | | Not applicable | | | | |
| 3. Is there congruity between the research methodology and the methods used to collect data? | | | | | | Yes | | | | No | | | | Unclear | | | | | Not applicable | | | | |
| 4. Is there congruity between the research methodology and the representation and analysis of data? | | | | | | Yes | | | | No | | | | Unclear | | | | | Not applicable | | | | |
| 5. Is there congruity between the research methodology and the interpretation of results? | | | | | | Yes | | | | No | | | | Unclear | | | | | Not applicable | | | | |
| 6. Is there a statement locating the researcher culturally or theoretically? | | | | | | Yes | | | | No | | | | Unclear | | | | | Not applicable | | | | |
| 7. Is the influence of the researcher on the research, and vice-versa, addressed? | | | | | | Yes | | | | No | | | | Unclear | | | | | Not applicable | | | | |
| 8. Are participants, and their voices, adequately represented? | | | | | | Yes | | | | No | | | | Unclear | | | | | Not applicable | | | | |
| 9. Is the research ethical according to current criteria or, for recent studies, and is there evidence of ethical approval by an appropriate body? | | | | | | Yes | | | | No | | | | Unclear | | | | | Not applicable | | | | |
| 10. Do the conclusions drawn in the research report flow from the analysis, or interpretation, of the data? | | | | | | Yes | | | | No | | | | Unclear | | | | | Not applicable | | | | |
| Overall appraisal: Include □ Exclude □ Seek further info □ | | | | | | | | | | | | | | | | | | | | | | | |
|  | | | | | | | | | | | | | | | | | | | | | | | |
| **G. The Quality Framework: Cabinet Office checklist for social research (last amended in 2003)**  **Website:** https://webarchive.nationalarchives.gov.uk/20140402165901/http://www.civilservice.gov.uk/wp-content/uploads/2011/09/a_quality_framework_tcm6-7314.pdf | | | | | | | | | | | | | | | | | | | | | | | |
| Major Components | | | | | | | | | | | | Response options | | | | | | | | | | | |
| FINDINGS | | | | | | | | | | | | | | | | | | | | | | | |
| 1. How credible are the findings? | | | | | | | | | | | |  | | | | | | | | | | | |
| 2. How has knowledge/ understanding been extended by the research? | | | | | | | | | | | |  | | | | | | | | | | | |
| 3. How well does the evaluation address its original aims and purpose? | | | | | | | | | | | |  | | | | | | | | | | | |
| 4. Scope for drawing wider inference – how well is this explained? | | | | | | | | | | | |  | | | | | | | | | | | |
| 5. How clear is the basis of evaluative appraisal? | | | | | | | | | | | |  | | | | | | | | | | | |
| DESIGN | | | | | | | | | | | | | | | | | | | | | | | |
| 6. How defensible is the research design? | | | | | | | | | | | |  | | | | | | | | | | | |
| SAMPLE | | | | | | | | | | | | | | | | | | | | | | | |
| 7. How well defended is the sample design/ target selection of cases/documents? | | | | | | | | | | | |  | | | | | | | | | | | |
| 8. Sample composition/case inclusion – how well is the eventual coverage described? | | | | | | | | | | | |  | | | | | | | | | | | |
| DATA COLLECTION | | | | | | | | | | | | | | | | | | | | | | | |
| 9. How well was the data collection carried out? | | | | | | | | | | | |  | | | | | | | | | | | |
| ANALYSIS | | | | | | | | | | | | | | | | | | | | | | | |
| 10. How well has the approach to, and formulation of, the analysis been conveyed? | | | | | | | | | | | |  | | | | | | | | | | | |
| 11. Contexts of data sources – how well are they retained and portrayed? | | | | | | | | | | | |  | | | | | | | | | | | |
| 12. How well has diversity of perspective and content been explored? | | | | | | | | | | | |  | | | | | | | | | | | |
| 13. How well has detail, depth and complexity (i.e. richness) of the data been conveyed? | | | | | | | | | | | |  | | | | | | | | | | | |
| REPORTING | | | | | | | | | | | | | | | | | | | | | | | |
| 14. How clear are the links between data, interpretation and conclusions – i.e. how well can the route to any conclusions be seen? | | | | | | | | | | | |  | | | | | | | | | | | |
| 15. How clear and coherent is the reporting? | | | | | | | | | | | |  | | | | | | | | | | | |
| REFLEXIVITY & NEUTRALITY | | | | | | | | | | | | | | | | | | | | | | | |
| 16. How clear are the assumptions/theoretical perspectives/values that have shaped the form and output of the evaluation? | | | | | | | | | | | |  | | | | | | | | | | | |
| ETHICS | | | | | | | | | | | | | | | | | | | | | | | |
| 17. What evidence is there of attention to ethical issues? | | | | | | | | | | | |  | | | | | | | | | | | |
| AUDITABILITY | | | | | | | | | | | | | | | | | | | | | | | |
| 18. How adequately has the research process been documented? | | | | | | | | | | | |  | | | | | | | | | | | |
|  | | | | | | | | | | | | | | | | | | | | | | | |
| **H. The Quality In Prognosis Studies (QIPS) tool (last released in 2013)** | | | | | | | | | | | | | | | | | | | | | | | |
| Major Components | | | | | | | | | | | | | | Response options | | | | | | | | | |
| **1. Study Participation**  Goal: To judge the risk of selection bias (likelihood that relationship between prognostic factor and outcome is different for participants and eligible non-participants) | | | | | | | | | | | | | | | | | | | | | | | |
| Source of target population | The source population or population of interest is adequately described for key characteristics (LIST) | | | | | | | | | | | | | High | | | Moderate | | | | | | Low |
| Method used to identify population | The sampling frame and recruitment are adequately described, including methods to identify the sample sufficient to limit potential bias (number and type used, e.g., referral patterns in health care) | | | | | | | | | | | | | High | | | Moderate | | | | | | Low |
| Recruitment period | Period of recruitment is adequately described | | | | | | | | | | | | | High | | | Moderate | | | | | | Low |
| Place of recruitment | Place of recruitment (setting and geographic location) are adequately described | | | | | | | | | | | | | High | | | Moderate | | | | | | Low |
| Inclusion and exclusion criteria | Inclusion and exclusion criteria are adequately described (e.g., including explicit diagnostic criteria or “zero time” description) | | | | | | | | | | | | | High | | | Moderate | | | | | | Low |
| Adequate study participation | There is adequate participation in the study by eligible individuals | | | | | | | | | | | | | High | | | Moderate | | | | | | Low |
| Baseline characteristics | The baseline study sample (i.e., individuals entering the study) is adequately described for key characteristics (LIST) | | | | | | | | | | | | | High | | | Moderate | | | | | | Low |
| **Summary Study participation** | The study sample represents the population of interest on key characteristics, sufficient to limit potential bias of the observed relationship between prognostic factor and outcome | | | | | | | | | | | | | High | | | Moderate | | | | | | Low |
| **2. Study Attrition**  Goal: To judge the risk of attrition bias (likelihood that relationship between prognostic factor and outcome are different for completing and non-completing participants) | | | | | | | | | | | | | | | | | | | | | | | |
| Proportion of baseline sample available for analysis | Response rate (i.e., proportion of study sample completing the study and providing outcome data) is adequate | | | | | | | | | | | | | High | | | Moderate | | | | | | Low |
| Attempts to collect information on participants who dropped out | Attempts to collect information on participants who dropped out of the study are described | | | | | | | | | | | | | High | | | Moderate | | | | | | Low |
| Reasons and potential impact of subjects lost to follow-up | Reasons for loss to follow-up are provided | | | | | | | | | | | | | High | | | Moderate | | | | | | Low |
| Outcome and prognostic factor information on those lost to follow-up | Participants lost to follow-up are adequately described for key characteristics (LIST) | | | | | | | | | | | | | High | | | Moderate | | | | | | Low |
|  | There are no important differences between key characteristics (LIST) and outcomes in participants who completed the study and those who did not | | | | | | | | | | | | | High | | | Moderate | | | | | | Low |
| **Study Attrition Summary** | Loss to follow-up (from baseline sample to study population analyzed) is not associated with key characteristics (i.e., the study data adequately represent the sample) sufficient to limit potential bias to the observed relationship between prognostic factor and outcome | | | | | | | | | | | | | High | | | Moderate | | | | | | Low |
| **3. Prognostic Factor Measurement**  Goal: To judge the risk of measurement bias related to how prognostic factor was measured (differential measurement of prognostic factor related to the level of outcome) | | | | | | | | | | | | | | | | | | | | | | | |
| Definition of the prognostic factor | A clear definition or description of 'Prognostic Factor' is provided (e.g., including dose, level, duration of exposure, and clear specification of the method of measurement) | | | | | | | | | | | | | High | | | Moderate | | | | | | Low |
| Valid and Reliable Measurement of prognostic factor | Method of prognostic factor measurement is adequately valid and reliable to limit misclassification bias (e.g., may include relevant outside sources of information on measurement properties, also characteristics, such as blind measurement and limited reliance on recall) | | | | | | | | | | | | | High | | | Moderate | | | | | | Low |
|  | Continuous variables are reported or appropriate cut-points (i.e., not data-dependent) are used | | | | | | | | | | | | | High | | | Moderate | | | | | | Low |
| Method and Setting of Prognostic Factor Measurement | The method and setting of measurement of prognostic factor is the same for all study participants | | | | | | | | | | | | | High | | | Moderate | | | | | | Low |
| Proportion of data on prognostic factor available for analysis | Adequate proportion of the study sample has complete data for prognostic factor variable | | | | | | | | | | | | | High | | | Moderate | | | | | | Low |
| Method used for missing data | Appropriate methods of imputation are used for missing 'Prognostic Factor' data | | | | | | | | | | | | | High | | | Moderate | | | | | | Low |
| **Prognostic Factor Measurement Summary** | Prognostic factor is adequately measured in study participants to sufficiently limit potential bias | | | | | | | | | | | | | High | | | Moderate | | | | | | Low |
| **4. Outcome Measurement**  Goal: To judge the risk of bias related to the measurement of outcome (differential measurement of outcome related to the baseline level of prognostic factor) | | | | | | | | | | | | | | | | | | | | | | | |
| Definition of the Outcome | A clear definition of outcome is provided, including duration of follow-up and level and extent of the outcome construct | | | | | | | | | | | | | High | | | Moderate | | | | | | Low |
| Valid and Reliable Measurement of Outcome | The method of outcome measurement used is adequately valid and reliable to limit misclassification bias (e.g., may include relevant outside sources of information on measurement properties, also characteristics, such as blind measurement and confirmation of outcome with valid and reliable test) | | | | | | | | | | | | | High | | | Moderate | | | | | | Low |
| Method and Setting of Outcome Measurement | The method and setting of outcome measurement is the same for all study participants | | | | | | | | | | | | | High | | | Moderate | | | | | | Low |
| **Outcome Measurement Summary** | Outcome of interest is adequately measured in study participants to sufficiently limit potential bias | | | | | | | | | | | | | High | | | Moderate | | | | | | Low |
| **5. Study Confounding**  Goal: To judge the risk of bias due to confounding (i.e. the effect of prognostic factor is distorted by another factor that is related to prognostic factor and outcome) | | | | | | | | | | | | | | | | | | | | | | | |
| Important Confounders Measured | All important confounders, including treatments (key variables in conceptual model: LIST), are measured | | | | | | | | | | | | | High | | | Moderate | | | | | | Low |
| Definition of the confounding factor | Clear definitions of the important confounders measured are provided (e.g., including dose, level, and duration of exposures) | | | | | | | | | | | | | High | | | Moderate | | | | | | Low |
| Valid and Reliable Measurement of Confounders | Measurement of all important confounders is adequately valid and reliable (e.g., may include relevant outside sources of information on measurement properties, also characteristics, such as blind measurement and limited reliance on recall) | | | | | | | | | | | | | High | | | Moderate | | | | | | Low |
| Method and Setting of Confounding Measurement | The method and setting of confounding measurement are the same for all study participants | | | | | | | | | | | | | High | | | Moderate | | | | | | Low |
| Method used for missing data | Appropriate methods are used if imputation is used for missing confounder data | | | | | | | | | | | | | High | | | Moderate | | | | | | Low |
| Appropriate Accounting for Confounding | Important potential confounders are accounted for in the study design (e.g., matching for key variables, stratification, or initial assembly of comparable groups) | | | | | | | | | | | | | High | | | Moderate | | | | | | Low |
|  | Important potential confounders are accounted for in the analysis (i.e., appropriate adjustment) | | | | | | | | | | | | | High | | | Moderate | | | | | | Low |
| **Study Confounding Summary** | Important potential confounders are appropriately accounted for, limiting potential bias with respect to the relationship between PF and outcome | | | | | | | | | | | | | High | | | Moderate | | | | | | Low |
| **6. Statistical Analysis and Reporting**  Goal: To judge the risk of bias related to the statistical analysis and presentation of results | | | | | | | | | | | | | | | | | | | | | | | |
| Presentation of analytical strategy | There is sufficient presentation of data to assess the adequacy of the analysis | | | | | | | | | | | | | High | | | Moderate | | | | | | Low |
| Model development strategy | The strategy for model building (i.e., inclusion of variables in the statistical model) is appropriate and is based on a conceptual framework or model | | | | | | | | | | | | | High | | | Moderate | | | | | | Low |
|  | The selected statistical model is adequate for the design of the study | | | | | | | | | | | | | High | | | Moderate | | | | | | Low |
| Reporting of results | There is no selective reporting of results | | | | | | | | | | | | | High | | | Moderate | | | | | | Low |
| **Statistical Analysis and Presentation Summary** | The statistical analysis is appropriate for the design of the study, limiting potential for presentation of invalid or spurious results | | | | | | | | | | | | | High | | | Moderate | | | | | | Low |
|  | | | | | | | | | | | | | | | | | | | | | | | |
| **I. The Prediction model Risk Of Bias ASsessment Tool (PROBAST) (last released in 2019)**  **Website:** http://www.probast.org | | | | | | | | | | | | | | | | | | | | | | | |
| Major Components | | | | | | Response options | | | | | | | | | | | | | | | | | |
| 1. Participants | | | | | | | | | | | | | | | | | | | | | | | |
| **Signaling questions** | | | | | | | | | | | | | | | | | | | | | | | |
| 1.1. Were appropriate data sources used, e.g., cohort, randomized controlled trial, or nested case-control study data? | | | Yes/ Probably yes | | | | | | | | No/ Probably no | | | | | | | | No information | | | | |
| 1.2. Were all inclusions andexclusions of participants appropriate? | | | Yes/ Probably yes | | | | | | | | No/ Probably no | | | | | | | | No information | | | | |
| **Risk of bias** | | | | | | | | | | | | | | | | | | | | | | | |
| Selection of participants | | | High | | | | | | | | Low | | | | | | | | Unclear | | | | |
| **Applicability** | | | | | | | | | | | | | | | | | | | | | | | |
| Included participants or setting does not match the review question | | | High | | | | | | | | Low | | | | | | | | Unclear | | | | |
| 2. Predictors | | | | | | | | | | | | | | | | | | | | | | | |
| 2.1. Were predictors defined andassessed in a similar way for allparticipants? | | | Yes/ Probably yes | | | | | | | | No/ Probably no | | | | | | | | No information | | | | |
| 2.2. Were predictor assessmentsmade without knowledge of outcome data? | | | Yes/ Probably yes | | | | | | | | No/ Probably no | | | | | | | | No information | | | | |
| 2.3. Are all predictors available at the time the model is intended to be used? | | | Yes/ Probably yes | | | | | | | | No/ Probably no | | | | | | | | No information | | | | |
| **Risk of bias** | | | | | | | | | | | | | | | | | | | | | | | |
| Predictors or their assessment | | | High | | | | | | | | Low | | | | | | | | Unclear | | | | |
| **Applicability** | | | | | | | | | | | | | | | | | | | | | | | |
| Definition, assessment, or timing of predictors does not match thereview question | | | High | | | | | | | | Low | | | | | | | | Unclear | | | | |
| 3. Outcome | | | | | | | | | | | | | | | | | | | | | | | |
| 3.1. Was the outcome determinedappropriately? | | | Yes/ Probably yes | | | | | | | | No/ Probably no | | | | | | | | No information | | | | |
| 3.2. Was a prespecified or standard outcome definition used? | | | Yes/ Probably yes | | | | | | | | No/ Probably no | | | | | | | | No information | | | | |
| 3.3. Were predictors excluded fromthe outcome definition? | | | Yes/ Probably yes | | | | | | | | No/ Probably no | | | | | | | | No information | | | | |
| 3.4. Was the outcome defined anddetermined in a similar way for all participants? | | | Yes/ Probably yes | | | | | | | | No/ Probably no | | | | | | | | No information | | | | |
| 3.5. Was the outcome determinedwithout knowledge of predictorinformation? | | | Yes/ Probably yes | | | | | | | | No/ Probably no | | | | | | | | No information | | | | |
| 3.6. Was the time interval betweenpredictors assessment and outcome determination appropriate? | | | Yes/ Probably yes | | | | | | | | No/ Probably no | | | | | | | | No information | | | | |
| **Risk of bias** | | | | | | | | | | | | | | | | | | | | | | | |
| Outcome or its determination | | | High | | | | | | | | Low | | | | | | | | Unclear | | | | |
| **Applicability** | | | | | | | | | | | | | | | | | | | | | | | |
| Its definition, timing, ordetermination does not match the review question | | | High | | | | | | | | Low | | | | | | | | Unclear | | | | |
| 4. Analysis | | | | | | | | | | | | | | | | | | | | | | | |
| 4.1. Were there a reasonable number of participants with the outcome? | | | Yes/ Probably yes | | | | | | | | No/ Probably no | | | | | | | | No information | | | | |
| 4.2. Were continuous and categoricalpredictors handled appropriately? | | | Yes/ Probably yes | | | | | | | | No/ Probably no | | | | | | | | No information | | | | |
| 4.3 Were all enrolled participantsincluded in the analysis? | | | Yes/ Probably yes | | | | | | | | No/ Probably no | | | | | | | | No information | | | | |
| 4.4. Were participants withmissingdata handled appropriately? | | | Yes/ Probably yes | | | | | | | | No/ Probably no | | | | | | | | No information | | | | |
| 4.5. Was selection of predictorsbased on univariable analysisavoided?^ꝉ^ | | | Yes/ Probably yes | | | | | | | | No/ Probably no | | | | | | | | No information | | | | |
| 4.6. Were complexities in the data (e.g., censoring, competing risks, sampling of control participants) accounted for appropriately? | | | Yes/ Probably yes | | | | | | | | No/ Probably no | | | | | | | | No information | | | | |
| 4.7. Were relevant modelperformance measures evaluatedappropriately? | | | Yes/ Probably yes | | | | | | | | No/ Probably no | | | | | | | | No information | | | | |
| 4.8. Were model overfitting, underfitting, and optimism inmodel performance accountedfor?^ꝉ^ | | | Yes/ Probably yes | | | | | | | | No/ Probably no | | | | | | | | No information | | | | |
| 4.9. Do predictors and their assignedweights in the final modelcorrespond to the results from thereported multivariable analysis?^ꝉ^ | | | Yes/ Probably yes | | | | | | | | No/ Probably no | | | | | | | | No information | | | | |
| **Risk of bias** | | | High | | | | | | | | Low | | | | | | | | Unclear | | | | |
| ^ꝉ^Development studies only | | | | | | | | | | | | | | | | | | | | | | | |
|  | | | | | | | | | | | | | | | | | | | | | | | |
| **J. The Critical Appraisal Skills Programme (CASP) Checklist for clinical prediction rule (last amended in 2018)**  **Website:** https://casp-uk.net/casp-tools-checklists/ | | | | | | | | | | | | | | | | | | | | | | | |
| Major Components | | | | | | | | | | | | | | Response options | | | | | | | | | |
| **Section A: Are the results of the study valid?** | | | | | | | | | | | | | | | | | | | | | | | |
| 1. Is the clinical prediction rule clearly defined? | | | | | | | | | | | | | | Yes | | | No | | | | | Can’t Tell | |
| 2. Did the population from which the rule was derived include an appropriate spectrum of patients? | | | | | | | | | | | | | | Yes | | | No | | | | | Can’t Tell | |
| 3. Was the rule validated in a different group of patients? | | | | | | | | | | | | | | Yes | | | No | | | | | Can’t Tell | |
| Is it worth continuing? | | | | | | | | | | | | | | | | | | | | | | | |
| 4. Were the predictor variables and the outcome evaluated in a blinded fashion? | | | | | | | | | | | | | | Yes | | | No | | | | | Can’t Tell | |
| 5. Were the predictor variables and the outcome evaluates in the whole sample selected initially? | | | | | | | | | | | | | | Yes | | | No | | | | | Can’t Tell | |
| 6. Are the statistical methods used to construct and validate the rule clearly described? | | | | | | | | | | | | | | Yes | | | No | | | | | Can’t Tell | |
| **Section B: What are the results?** | | | | | | | | | | | | | | | | | | | | | | | |
| 7. Can the performance of the rule be calculated? | | | | | | | | | | | | | |  | | | | | | | | | |
| 8. How precise was the estimate of the treatment effect?  (did they try to refine the rule with other variables to see whether the precision could be improved or the rule simplified?) | | | | | | | | | | | | | |  | | | | | | | | | |
| **Section C: Will the results help locally? Are the findings applicable to the scenario?** | | | | | | | | | | | | | | | | | | | | | | | |
| 9. Would the prediction rule be reliable and the results interpretable if used for your patient? | | | | | | | | | | | | | | Yes | | | No | | | | | Can’t Tell | |
| 10. Is the rule acceptable in your case? | | | | | | | | | | | | | | Yes | | | No | | | | | Can’t Tell | |
| 11. Would the results of the rule modify your decision about the management of the patient, or the information you can give to him/her? | | | | | | | | | | | | | | Yes | | | No | | | | | Can’t Tell | |
|  | | | | | | | | | | | | | | | | | | | | | | | |
| **K. The Joanna Briggs Institute (JBI) Critical Appraisal Checklist for text and expert opinion papers (last amended in 2017)**  **Website:** https://joannabriggs.org/critical_appraisal_tools  https://wiki.joannabriggs.org/display/MANUAL/Appendix+4.1%3A+JBI+Critical+Appraisal+Checklist+for+Text+and+Opinion+Papers | | | | | | | | | | | | | | | | | | | | | | | |
| Major Components | | | | | | Response options | | | | | | | | | | | | | | | | | |
| 1. Is the source of the opinion clearly identified? | | | | | | Yes | | | | No | | | | Unclear | | | | | Not applicable | | | | |
| 2. Does the source of opinion have standing in the field of expertise? | | | | | | Yes | | | | No | | | | Unclear | | | | | Not applicable | | | | |
| 3. Are the interests of the relevant population the central focus of the opinion? | | | | | | Yes | | | | No | | | | Unclear | | | | | Not applicable | | | | |
| 4. Is the stated position the result of an analytical process, and is there logic in the opinion expressed? | | | | | | Yes | | | | No | | | | Unclear | | | | | Not applicable | | | | |
| 5. Is there reference to the extant literature? | | | | | | Yes | | | | No | | | | Unclear | | | | | Not applicable | | | | |
| 6. Is any incongruence with the literature/ sources logically defended? | | | | | | Yes | | | | No | | | | Unclear | | | | | Not applicable | | | | |
| Overall appraisal: Include □ Exclude □ Seek further info □ | | | | | | | | | | | | | | | | | | | | | | | |
|  | | | | | | | | | | | | | | | | | | | | | | | |
| **L. The COnsensus-based Standards for the selection of health Measurement INstruments (COSMIN) Risk of Bias checklist (last amended in July 2018)**  **Website:** https://www.cosmin.nl/tools/checklists-assessing-methodological-study-qualities/ | | | | | | | | | | | | | | | | | | | | | | | |
| **LA. The patient - reported outcome measure (PROM) development** | | | | | | | | | | | | | | | | | | | | | | | |
| Major Components | | Response options | | | | | | | | | | | | | | | | | | | | | |
| **1a. PROM design** | | | | | | | | | | | | | | | | | | | | | | | |
| General design requirements | | | | | | | | | | | | | | | | | | | | | | | |
| 1. Is a clear description provided of the construct to be measured? | | Very good | | | Adequate | | | | Doubtful | | | | Inadequate | | | | | | | Not applicable | | | |
| 2. Is the origin of the construct clear: was a theory, conceptual framework or disease model used or clear rationale provided to define the construct to be measured? | | Very good | | | Adequate | | | | Doubtful | | | | Inadequate | | | | | | | Not applicable | | | |
| 3. Is a clear description provided of the target population for which the PROM was developed? | | Very good | | | Adequate | | | | Doubtful | | | | Inadequate | | | | | | | Not applicable | | | |
| 4. Is a clear description provided of the context of use | | Very good | | | Adequate | | | | Doubtful | | | | Inadequate | | | | | | | Not applicable | | | |
| 5. Was the PROM development study performed in a sample representing the target population for which the PROM was developed? | | Very good | | | Adequate | | | | Doubtful | | | | Inadequate | | | | | | | Not applicable | | | |
| Concept elicitation (relevance and comprehensiveness) | | | | | | | | | | | | | | | | | | | | | | | |
| 6. Was an appropriate qualitative data collection method used to identify relevant items for a new PROM? | | Very good | | | Adequate | | | | Doubtful | | | | Inadequate | | | | | | | Not applicable | | | |
| 7. Were skilled group moderators/interviewers used? | | Very good | | | Adequate | | | | Doubtful | | | | Inadequate | | | | | | | Not applicable | | | |
| 8. Were the group meetings or interviews based on an appropriate topic or interview guide? | | Very good | | | Adequate | | | | Doubtful | | | | Inadequate | | | | | | | Not applicable | | | |
| 9. Were the group meetings or interviews recorded and transcribed verbatim? | | Very good | | | Adequate | | | | Doubtful | | | | Inadequate | | | | | | | Not applicable | | | |
| 10. Was an appropriate approach used to analyse the data? | | Very good | | | Adequate | | | | Doubtful | | | | Inadequate | | | | | | | Not applicable | | | |
| 11. Was at least part of the data coded independently? | | Very good | | | Adequate | | | | Doubtful | | | | Inadequate | | | | | | | Not applicable | | | |
| 12. Was data collection continued until saturation was reached? | | Very good | | | Adequate | | | | Doubtful | | | | Inadequate | | | | | | | Not applicable | | | |
| 13. For quantitative studies (surveys): was the sample size appropriate? | | Very good | | | Adequate | | | | Doubtful | | | | Inadequate | | | | | | | Not applicable | | | |
| **1b. Cognitive interview study or other pilot test** | | | | | | | | | | | | | | | | | | | | | | | |
| 14. Was a cognitive interview study or other pilot test conducted? | | Very good | | | Adequate | | | | Doubtful | | | | Inadequate | | | | | | | Not applicable | | | |
| General design requirements | | | | | | | | | | | | | | | | | | | | | | | |
| 15. Was the cognitive interview study or other pilot test performed in a sample representing the target population? | | Very good | | | Adequate | | | | Doubtful | | | | Inadequate | | | | | | | Not applicable | | | |
| Comprehensibility | | | | | | | | | | | | | | | | | | | | | | | |
| 16. Were patients asked about the comprehensibility of the PROM? | | Very good | | | Adequate | | | | Doubtful | | | | Inadequate | | | | | | | Not applicable | | | |
| 17. Were all items tested in their final form? | | Very good | | | Adequate | | | | Doubtful | | | | Inadequate | | | | | | | Not applicable | | | |
| 18. Was an appropriate qualitative method used to assess the comprehensibility of the PROM instructions, items, response options, and recall period? | | Very good | | | Adequate | | | | Doubtful | | | | Inadequate | | | | | | | Not applicable | | | |
| 19. Was each item tested in an appropriate number of patients?  For qualitative studies  For quantitative (survey) studies | | Very good | | | Adequate | | | | Doubtful | | | | Inadequate | | | | | | | Not applicable | | | |
| 20. Were skilled interviewers used? | | Very good | | | Adequate | | | | Doubtful | | | | Inadequate | | | | | | | Not applicable | | | |
| 21. Were the interviews based on an appropriate interview guide? | | Very good | | | Adequate | | | | Doubtful | | | | Inadequate | | | | | | | Not applicable | | | |
| 22. Were the interviews recorded and transcribed verbatim? | | Very good | | | Adequate | | | | Doubtful | | | | Inadequate | | | | | | | Not applicable | | | |
| 23. Was an appropriate approach used to analyse the data? | | Very good | | | Adequate | | | | Doubtful | | | | Inadequate | | | | | | | Not applicable | | | |
| 24. Were at least two researchers involved in the analysis? | | Very good | | | Adequate | | | | Doubtful | | | | Inadequate | | | | | | | Not applicable | | | |
| 25. Were problems regarding the comprehensibility of the PROM instructions, items, response options, and recall period appropriately addressed by adapting the PROM? | | Very good | | | Adequate | | | | Doubtful | | | | Inadequate | | | | | | | Not applicable | | | |
| Comprehensiveness | | | | | | | | | | | | | | | | | | | | | | | |
| 26. Were patients asked about the comprehensiveness of the PROM? | | Very good | | | Adequate | | | | Doubtful | | | | Inadequate | | | | | | | Not applicable | | | |
| 27. Was the final set of items tested? | | Very good | | | Adequate | | | | Doubtful | | | | Inadequate | | | | | | | Not applicable | | | |
| 28. Was an appropriate method used for assessing the comprehensiveness of the PROM? | | Very good | | | Adequate | | | | Doubtful | | | | Inadequate | | | | | | | Not applicable | | | |
| 29. Was each item tested in an appropriate number of patients?  For qualitative studies  For quantitative (survey) studies | | Very good | | | Adequate | | | | Doubtful | | | | Inadequate | | | | | | | Not applicable | | | |
| 30. Were skilled interviewers used? | | Very good | | | Adequate | | | | Doubtful | | | | Inadequate | | | | | | | Not applicable | | | |
| 31. Were the interviews based on an appropriate interview guide? | | Very good | | | Adequate | | | | Doubtful | | | | Inadequate | | | | | | | Not applicable | | | |
| 32. Were the interviews recorded and transcribed verbatim? | | Very good | | | Adequate | | | | Doubtful | | | | Inadequate | | | | | | | Not applicable | | | |
| 33. Was an appropriate approach used to analyse the data? | | Very good | | | Adequate | | | | Doubtful | | | | Inadequate | | | | | | | Not applicable | | | |
| 34. Were at least two researchers involved in the analysis? | | Very good | | | Adequate | | | | Doubtful | | | | Inadequate | | | | | | | Not applicable | | | |
| 35. Were problems regarding the comprehensiveness of the PROM appropriately addressed by adapting the PROM? | | Very good | | | Adequate | | | | Doubtful | | | | Inadequate | | | | | | | Not applicable | | | |
|  | | | | | | | | | | | | | | | | | | | | | | | |
| **LB. Content validity** | | | | | | | | | | | | | | | | | | | | | | | |
| **2a. Asking patients about relevance** | | | | | | | | | | | | | | | | | | | | | | | |
| Design requirements | | | | | | | | | | | | | | | | | | | | | | | |
| 1. Was an appropriate method used to ask patients whether each item is relevant for their experience with the condition? | | Very good | | | Adequate | | | | Doubtful | | | | Inadequate | | | | | | | Not applicable | | | |
| 2. Was each item tested in an appropriate number of patients?  For qualitative studies  For quantitative (survey) studies | | Very good | | | Adequate | | | | Doubtful | | | | Inadequate | | | | | | | Not applicable | | | |
| 3. Were skilled group moderators/interviewers used? | | Very good | | | Adequate | | | | Doubtful | | | | Inadequate | | | | | | | Not applicable | | | |
| 4. Were the group meetings or interviews based on an appropriate topic or interview guide? | | Very good | | | Adequate | | | | Doubtful | | | | Inadequate | | | | | | | Not applicable | | | |
| 5. Were the group meetings or interviews recorded and transcribed verbatim? | | Very good | | | Adequate | | | | Doubtful | | | | Inadequate | | | | | | | Not applicable | | | |
| Analyses | | | | | | | | | | | | | | | | | | | | | | | |
| 6. Was an appropriate approach used to analyse the data? | | Very good | | | Adequate | | | | Doubtful | | | | Inadequate | | | | | | | Not applicable | | | |
| 7. Were at least two researchers involved in the analysis? | | Very good | | | Adequate | | | | Doubtful | | | | Inadequate | | | | | | | Not applicable | | | |
| **2b Asking patients about comprehensiveness** | | | | | | | | | | | | | | | | | | | | | | | |
| Design requirements | | | | | | | | | | | | | | | | | | | | | | | |
| 8. Was an appropriate method used for assessing the comprehensiveness of the PROM? | | Very good | | | Adequate | | | | Doubtful | | | | Inadequate | | | | | | | Not applicable | | | |
| 9. Was each item tested in an appropriate number of patients?  For qualitative studies  For quantitative (survey) studies | | Very good | | | Adequate | | | | Doubtful | | | | Inadequate | | | | | | | Not applicable | | | |
| 10. Were skilled group moderators/interviewers used? | | Very good | | | Adequate | | | | Doubtful | | | | Inadequate | | | | | | | Not applicable | | | |
| 11. Were the group meetings or interviews based on an appropriate topic or interview guide? | | Very good | | | Adequate | | | | Doubtful | | | | Inadequate | | | | | | | Not applicable | | | |
| 12. Were the group meetings or interviews recorded and transcribed verbatim? | | Very good | | | Adequate | | | | Doubtful | | | | Inadequate | | | | | | | Not applicable | | | |
| Analyses | | | | | | | | | | | | | | | | | | | | | | | |
| 13. Was an appropriate approach used to analyse the data? | | Very good | | | Adequate | | | | Doubtful | | | | Inadequate | | | | | | | Not applicable | | | |
| 14. Were at least two researchers involved in the analysis? | | Very good | | | Adequate | | | | Doubtful | | | | Inadequate | | | | | | | Not applicable | | | |
| **2c Asking patients about comprehensibility** | | | | | | | | | | | | | | | | | | | | | | | |
| Design requirements | | | | | | | | | | | | | | | | | | | | | | | |
| 15. Was an appropriate qualitative method used for assessing the comprehensibility of the PROM instructions, items, response options, and recall period? | | Very good | | | Adequate | | | | Doubtful | | | | Inadequate | | | | | | | Not applicable | | | |
| 16. Was each item tested in an appropriate number of patients?  For qualitative studies  For quantitative (survey) studies | | Very good | | | Adequate | | | | Doubtful | | | | Inadequate | | | | | | | Not applicable | | | |
| 17. Were skilled group moderators/interviewers used? | | Very good | | | Adequate | | | | Doubtful | | | | Inadequate | | | | | | | Not applicable | | | |
| 18. Were the group meetings or interviews based on an appropriate topic or interview guide? | | Very good | | | Adequate | | | | Doubtful | | | | Inadequate | | | | | | | Not applicable | | | |
| 19. Were the group meetings or interviews recorded and transcribed verbatim? | | Very good | | | Adequate | | | | Doubtful | | | | Inadequate | | | | | | | Not applicable | | | |
| Analyses | | | | | | | | | | | | | | | | | | | | | | | |
| 20. Was an appropriate approach used to analyse the data? | | Very good | | | Adequate | | | | Doubtful | | | | Inadequate | | | | | | | Not applicable | | | |
| 21. Were at least two researchers involved in the analysis? | | Very good | | | Adequate | | | | Doubtful | | | | Inadequate | | | | | | | Not applicable | | | |
| **2d. Asking professionals about relevance** | | | | | | | | | | | | | | | | | | | | | | | |
| Design requirements | | | | | | | | | | | | | | | | | | | | | | | |
| 22. Was an appropriate method used to ask professionals whether each item is relevant for the construct of interest? | | Very good | | | Adequate | | | | Doubtful | | | | Inadequate | | | | | | | Not applicable | | | |
| 23. Were professionals from all relevant disciplines included? | | Very good | | | Adequate | | | | Doubtful | | | | Inadequate | | | | | | | Not applicable | | | |
| 24. Was each item tested in an appropriate number of professionals?  For qualitative studies  For quantitative (survey) studies | | Very good | | | Adequate | | | | Doubtful | | | | Inadequate | | | | | | | Not applicable | | | |
| Analyses | | | | | | | | | | | | | | | | | | | | | | | |
| 25. Was an appropriate approach used to analyse the data? | | Very good | | | Adequate | | | | Doubtful | | | | Inadequate | | | | | | | Not applicable | | | |
| 26. Were at least two researchers involved in the analysis? | | Very good | | | Adequate | | | | Doubtful | | | | Inadequate | | | | | | | Not applicable | | | |
| **2e. Asking professionals about comprehensiveness** | | | | | | | | | | | | | | | | | | | | | | | |
| Design requirement | | | | | | | | | | | | | | | | | | | | | | | |
| 27. Was an appropriate method used for assessing the comprehensiveness of the PROM? | | Very good | | | Adequate | | | | Doubtful | | | | Inadequate | | | | | | | Not applicable | | | |
| 28. Were professionals from all relevant disciplines included? | | Very good | | | Adequate | | | | Doubtful | | | | Inadequate | | | | | | | Not applicable | | | |
| 29. Was each item tested in an appropriate number of professionals?  For qualitative studies  For quantitative (survey) studies | | Very good | | | Adequate | | | | Doubtful | | | | Inadequate | | | | | | | Not applicable | | | |
| Analyses | | | | | | | | | | | | | | | | | | | | | | | |
| 30. Was an appropriate approach used to analyse the data? | | Very good | | | Adequate | | | | Doubtful | | | | Inadequate | | | | | | | Not applicable | | | |
| 31. Were at least two researchers involved in the analysis? | | Very good | | | Adequate | | | | Doubtful | | | | Inadequate | | | | | | | Not applicable | | | |
|  | | | | | | | | | | | | | | | | | | | | | | | |
| **LC. Structural validity** | | | | | | | | | | | | | | | | | | | | | | | |
| Does the scale consist of effect indicators, i.e. is it based on a reflective model? ^1^ | | Yes | | | No | | | | / | | | | / | | | | | | | / | | | |
| Does the study concern unidimensionality or structural validity? ^2^ | | Unidimensionality | | | | | | | Structural validity | | | | | | | | | | | / | | | |
| Statistical methods | | | | | | | | | | | | | | | | | | | | | | | |
| 1. For classical test theory (CTT): Was exploratory or confirmatory factor analysis performed? | | Very good | | | Adequate | | | | Doubtful | | | | Inadequate | | | | | | | Not applicable | | | |
| 2. For item response theory (IRT)/ Rasch: does the chosen model fit to the research question? | | Very good | | | Adequate | | | | Doubtful | | | | Inadequate | | | | | | | Not applicable | | | |
| 3. Was the sample size included in the analysis adequate? | | Very good | | | Adequate | | | | Doubtful | | | | Inadequate | | | | | | | Not applicable | | | |
| Other | | | | | | | | | | | | | | | | | | | | | | | |
| 4. Were there any other important flaws in the design or statistical methods of the study? | | Very good | | | Adequate | | | | Doubtful | | | | Inadequate | | | | | | | Not applicable | | | |
| ^1^ If the scale is not based on a reflective model, unidimensionality or structural validity is not relevant.  ^2^ In a systematic review, it is helpful to make a distinction between studies where factor analysis is performed on each (sub)scale separately to evaluate whether the (sub)scales are unidimensional (unidimensionality studies) and studies where factor analysis is performed on all items of an instrument to evaluate the (expected) number of subscales in the instrument and the clustering of items within subscales (structural validity studies). | | | | | | | | | | | | | | | | | | | | | | | |
|  | | | | | | | | | | | | | | | | | | | | | | | |
| **LD. Internal consistency** | | | | | | | | | | | | | | | | | | | | | | | |
| Does the scale consist of effect indicators, i.e. is it based on a reflective model? ^1^ | | Yes | | | No | | | | / | | | | / | | | | | | | / | | | |
| Design requirements | | | | | | | | | | | | | | | | | | | | | | | |
| 1. Was an internal consistency statistic calculated for each unidimensional scale or subscale separately? | | Very good | | | Adequate | | | | Doubtful | | | | Inadequate | | | | | | | Not applicable | | | |
| Statistical methods | | | | | | | | | | | | | | | | | | | | | | | |
| 2. For continuous scores: Was Cronbach’s alpha or omega calculated? | | Very good | | | Adequate | | | | Doubtful | | | | Inadequate | | | | | | | Not applicable | | | |
| 3. For dichotomous scores: Was Cronbach’s alpha or KR‐ 20 calculated? | | Very good | | | Adequate | | | | Doubtful | | | | Inadequate | | | | | | | Not applicable | | | |
| 4. For item response theory (IRT) - based scores: Was standard error of the theta (SE (θ)) or reliability coefficient of estimated latent trait value (index of (subject or item) separation) calculated? | | Very good | | | Adequate | | | | Doubtful | | | | Inadequate | | | | | | | Not applicable | | | |
| Other | | | | | | | | | | | | | | | | | | | | | | | |
| 5. Were there any other important flaws in the design or statistical methods of the study? | | Very good | | | Adequate | | | | Doubtful | | | | Inadequate | | | | | | | Not applicable | | | |
| ^1^ If the scale is not based on a reflective model, internal consistency is not relevant. | | | | | | | | | | | | | | | | | | | | | | | |
|  | | | | | | | | | | | | | | | | | | | | | | | |
| **LE. Cross - cultural validity\Measurement invariance** | | | | | | | | | | | | | | | | | | | | | | | |
| Design requirements | | | | | | | | | | | | | | | | | | | | | | | |
| 1. Were the samples similar for relevant characteristics except for the group variable? | | Very good | | | Adequate | | | | Doubtful | | | | Inadequate | | | | | | | Not applicable | | | |
| Statistical methods | | | | | | | | | | | | | | | | | | | | | | | |
| 2. Was an appropriate approach used to analyse the data? | | Very good | | | Adequate | | | | Doubtful | | | | Inadequate | | | | | | | Not applicable | | | |
| 3. Was the sample size included in the analysis adequate? | | Very good | | | Adequate | | | | Doubtful | | | | Inadequate | | | | | | | Not applicable | | | |
| Other | | | | | | | | | | | | | | | | | | | | | | | |
| 4. Were there any other important flaws in the design or statistical methods of the study? | | Very good | | | Adequate | | | | Doubtful | | | | Inadequate | | | | | | | Not applicable | | | |
|  | | | | | | | | | | | | | | | | | | | | | | | |
| **LF. Reliability** | | | | | | | | | | | | | | | | | | | | | | | |
| Design requirements | | | | | | | | | | | | | | | | | | | | | | | |
| 1. Were patients stable in the interim period on the construct to be measured? | | Very good | | | Adequate | | | | Doubtful | | | | Inadequate | | | | | | | Not applicable | | | |
| 2. Was the time interval appropriate? | | Very good | | | Adequate | | | | Doubtful | | | | Inadequate | | | | | | | Not applicable | | | |
| 3. Were the test conditions similar for the measurements? e.g. type of administration, environment, instructions | | Very good | | | Adequate | | | | Doubtful | | | | Inadequate | | | | | | | Not applicable | | | |
| Statistical methods | | | | | | | | | | | | | | | | | | | | | | | |
| 4. For continuous scores: Was an intraclass correlation coefficient (ICC) calculated? | | Very good | | | Adequate | | | | Doubtful | | | | Inadequate | | | | | | | Not applicable | | | |
| 5. For dichotomous/ nominal/ ordinal scores: Was kappa calculated? | | Very good | | | Adequate | | | | Doubtful | | | | Inadequate | | | | | | | Not applicable | | | |
| 6. For ordinal scores: Was a weighted kappa calculated? | | Very good | | | Adequate | | | | Doubtful | | | | Inadequate | | | | | | | Not applicable | | | |
| 7. For ordinal scores: Was the weighting scheme described? e.g. linear, quadratic | | Very good | | | Adequate | | | | Doubtful | | | | Inadequate | | | | | | | Not applicable | | | |
| Other | | | | | | | | | | | | | | | | | | | | | | | |
| 8. Were there any other important flaws in the design or statistical methods of the study? | | Very good | | | Adequate | | | | Doubtful | | | | Inadequate | | | | | | | Not applicable | | | |
|  | | | | | | | | | | | | | | | | | | | | | | | |
| **LG. Measurement error** | | | | | | | | | | | | | | | | | | | | | | | |
| Design requirements | | | | | | | | | | | | | | | | | | | | | | | |
| 1. Were patients stable in the interim period on the construct to be measured? | | Very good | | | Adequate | | | | Doubtful | | | | Inadequate | | | | | | | Not applicable | | | |
| 2. Was the time interval appropriate? | | Very good | | | Adequate | | | | Doubtful | | | | Inadequate | | | | | | | Not applicable | | | |
| 3. Were the test conditions similar for the measurements? (e.g. type of administration, environment, instructions) | | Very good | | | Adequate | | | | Doubtful | | | | Inadequate | | | | | | | Not applicable | | | |
| Statistical methods | | | | | | | | | | | | | | | | | | | | | | | |
| 4. For continuous scores: Was the Standard Error of Measurement (SEM), Smallest Detectable Change (SDC) or Limits of Agreement (LoA) calculated? | | Very good | | | Adequate | | | | Doubtful | | | | Inadequate | | | | | | | Not applicable | | | |
| 5. For dichotomous/nominal/ordinal scores: Was the percentage (positive and negative) agreement calculated? | | Very good | | | Adequate | | | | Doubtful | | | | Inadequate | | | | | | | Not applicable | | | |
| Other | | | | | | | | | | | | | | | | | | | | | | | |
| 6. Were there any other important flaws in the design or statistical methods of the study? | | Very good | | | Adequate | | | | Doubtful | | | | Inadequate | | | | | | | Not applicable | | | |
|  | | | | | | | | | | | | | | | | | | | | | | | |
| **LH. Criterion validity** | | | | | | | | | | | | | | | | | | | | | | | |
| Statistical methods | | | | | | | | | | | | | | | | | | | | | | | |
| 1. For continuous scores: Were correlations, or the area under the receiver operating curve calculated? | | Very good | | | Adequate | | | | Doubtful | | | | Inadequate | | | | | | | Not applicable | | | |
| 2. For dichotomous scores: Were sensitivity and specificity determined? | | Very good | | | Adequate | | | | Doubtful | | | | Inadequate | | | | | | | Not applicable | | | |
| Other | | | | | | | | | | | | | | | | | | | | | | | |
| 3. Were there any other important flaws in the design or statistical methods of the study? | | Very good | | | Adequate | | | | Doubtful | | | | Inadequate | | | | | | | Not applicable | | | |
|  | | | | | | | | | | | | | | | | | | | | | | | |
| **LI. Hypotheses testing for construct validity** | | | | | | | | | | | | | | | | | | | | | | | |
| **9a. Comparison with other outcome measurement instruments (convergent validity)** | | | | | | | | | | | | | | | | | | | | | | | |
| Design requirements | | | | | | | | | | | | | | | | | | | | | | | |
| 1. Is it clear what the comparator instrument(s) measure(s)? | | Very good | | | Adequate | | | | Doubtful | | | | Inadequate | | | | | | | Not applicable | | | |
| 2. Were the measurement properties of the comparator instrument(s) sufficient? | | Very good | | | Adequate | | | | Doubtful | | | | Inadequate | | | | | | | Not applicable | | | |
| Statistical methods | | | | | | | | | | | | | | | | | | | | | | | |
| 3. Was the statistical method appropriate for the hypotheses to be tested? | | Very good | | | Adequate | | | | Doubtful | | | | Inadequate | | | | | | | Not applicable | | | |
| Other | | | | | | | | | | | | | | | | | | | | | | | |
| 4. Were there any other important flaws in the design or statistical methods of the study? | | Very good | | | Adequate | | | | Doubtful | | | | Inadequate | | | | | | | Not applicable | | | |
| **9b. Comparison between subgroups (discriminative or known - groups validity)** | | | | | | | | | | | | | | | | | | | | | | | |
| Design requirements | | Very good | | | Adequate | | | | Doubtful | | | | Inadequate | | | | | | | Not applicable | | | |
| 5. Was an adequate description provided of important characteristics of the subgroups? | | Very good | | | Adequate | | | | Doubtful | | | | Inadequate | | | | | | | Not applicable | | | |
| Statistical methods | | Very good | | | Adequate | | | | Doubtful | | | | Inadequate | | | | | | | Not applicable | | | |
| 6. Was the statistical method appropriate for the hypotheses to be tested? | | Very good | | | Adequate | | | | Doubtful | | | | Inadequate | | | | | | | Not applicable | | | |
| Other | | | | | | | | | | | | | | | | | | | | | | | |
| 7. Were there any other important flaws in the design or statistical methods of the study? | | Very good | | | Adequate | | | | Doubtful | | | | Inadequate | | | | | | | Not applicable | | | |
|  | | | | | | | | | | | | | | | | | | | | | | | |
| **LJ. Responsiveness** | | | | | | | | | | | | | | | | | | | | | | | |
| **10a. Criterion approach (i.e. comparison to a gold standard)** | | | | | | | | | | | | | | | | | | | | | | | |
| Statistical methods | | | | | | | | | | | | | | | | | | | | | | | |
| 1. For continuous scores: Were correlations between change scores, or the area under the Receiver Operator Curve (ROC) curve calculated? | | Very good | | | Adequate | | | | Doubtful | | | | Inadequate | | | | | | | Not applicable | | | |
| 2. For dichotomous scales: Were sensitivity and specificity (changed versus not changed) determined? | | Very good | | | Adequate | | | | Doubtful | | | | Inadequate | | | | | | | Not applicable | | | |
| Other | | | | | | | | | | | | | | | | | | | | | | | |
| 3. Were there any other important flaws in the design or statistical methods of the study? | | Very good | | | Adequate | | | | Doubtful | | | | Inadequate | | | | | | | Not applicable | | | |
| **10b. Construct approach (i.e. hypotheses testing; comparison with other outcome measurement instruments)** | | | | | | | | | | | | | | | | | | | | | | | |
| Design requirements | | | | | | | | | | | | | | | | | | | | | | | |
| 4. Is it clear what the comparator instrument(s) measure(s)? | | Very good | | | Adequate | | | | Doubtful | | | | Inadequate | | | | | | | Not applicable | | | |
| 5. Were the measurement properties of the comparator instrument(s) sufficient? | | Very good | | | Adequate | | | | Doubtful | | | | Inadequate | | | | | | | Not applicable | | | |
| Statistical methods | | | | | | | | | | | | | | | | | | | | | | | |
| 6. Was the statistical method appropriate for the hypotheses to be tested? | | Very good | | | Adequate | | | | Doubtful | | | | Inadequate | | | | | | | Not applicable | | | |
| Other | | | | | | | | | | | | | | | | | | | | | | | |
| 7. Were there any other important flaws in the design or statistical methods of the study? | | Very good | | | Adequate | | | | Doubtful | | | | Inadequate | | | | | | | Not applicable | | | |
| **10c. Construct approach: (i.e. hypotheses testing: comparison between subgroups)** | | | | | | | | | | | | | | | | | | | | | | | |
| Design requirements | | | | | | | | | | | | | | | | | | | | | | | |
| 8. Was an adequate description provided of important characteristics of the subgroups? | | Very good | | | Adequate | | | | Doubtful | | | | Inadequate | | | | | | | Not applicable | | | |
| Statistical methods | | | | | | | | | | | | | | | | | | | | | | | |
| 9. Was the statistical method appropriate for the hypotheses to be tested? | | Very good | | | Adequate | | | | Doubtful | | | | Inadequate | | | | | | | Not applicable | | | |
| Other | | | | | | | | | | | | | | | | | | | | | | | |
| 10. Were there any other important flaws in the design or statistical methods of the study? | | Very good | | | Adequate | | | | Doubtful | | | | Inadequate | | | | | | | Not applicable | | | |
| **10d. Construct approach: (i.e. hypotheses testing: before and after intervention)** | | | | | | | | | | | | | | | | | | | | | | | |
| Design requirements | | | | | | | | | | | | | | | | | | | | | | | |
| 11. Was an adequate description provided of the intervention given? | | Very good | | | Adequate | | | | Doubtful | | | | Inadequate | | | | | | | Not applicable | | | |
| Statistical methods | | | | | | | | | | | | | | | | | | | | | | | |
| 12. Was the statistical method appropriate for the hypotheses to be tested? | | Very good | | | Adequate | | | | Doubtful | | | | Inadequate | | | | | | | Not applicable | | | |
| Other | | | | | | | | | | | | | | | | | | | | | | | |
| 13. Were there any other important flaws in the design or statistical methods of the study? | | Very good | | | Adequate | | | | Doubtful | | | | Inadequate | | | | | | | Not applicable | | | |
