# Supplementary material for: Methodological quality (risk of bias) assessment tools for primary and secondary medical studies: what are they and which is better?
Source: Mil Med Res. 2020 Feb 29;7:7. doi: 10.1186/s40779-020-00238-8 (PMC7049186; doi:10.1186/s40779-020-00238-8)
Supplement: Supplementary file 4 — Additional file 4: Table S4. Major components of the tools for assessing secondary medical studies [file 40779_2020_238_MOESM4_ESM.docx]

**Table S4. Major components of the tools for assessing secondary medical studies**

| **A. The A Measurement Tool to Assess Systematic Reviews (AMSTAR) – 2 for systematic review and meta-analysis ((last introduced in 2017)**  **Website:** http://www.amstar.ca/ | | | | | | | | | | | | | | | | | | | | | |
| --- | --- | --- | --- | --- | --- | --- | --- | --- | --- | --- | --- | --- | --- | --- | --- | --- | --- | --- | --- | --- | --- |
| Major Components | | | | | | | | | Response options | | | | | | | | | | | | |
| 1. Did the research questions and inclusion criteria for the review include the components of PICO? | | | | | | | | | Yes | | | No | | | | / | | | | | |
| 2. Did the report of the review contain an explicit statement that the review methods were established prior to the conduct of the review and did the report justify any significant deviations from the protocol? | | | | | | | | | Yes | | | No | | | | Partial Yes | | | | | |
| 3. Did the review authors explain their selection of the study designs for inclusion in the review? | | | | | | | | | Yes | | | No | | | | / | | | | | |
| 4. Did the review authors use a comprehensive literature search strategy? | | | | | | | | | Yes | | | No | | | | Partial Yes | | | | | |
| 5. Did the review authors perform study selection in duplicate? | | | | | | | | | Yes | | | No | | | | / | | | | | |
| 6. Did the review authors perform data extraction in duplicate? | | | | | | | | | Yes | | | No | | | | / | | | | | |
| 7. Did the review authors provide a list of excluded studies and justify the exclusions? | | | | | | | | | Yes | | | No | | | | Partial Yes | | | | | |
| 8. Did the review authors describe the included studies in adequate detail? | | | | | | | | | Yes | | | No | | | | Partial Yes | | | | | |
| 9. Did the review authors use a satisfactory technique for assessing the risk of bias (RoB) in individual studies that were included in the review? | | | | | | | | | Yes | | | No | | | | Partial Yes | | | | | |
| 10. Did the review authors report on the sources of funding for the studies included in the review? | | | | | | | | | Yes | | | No | | | | / | | | | | |
| 11. If meta-analysis was performed did the review authors use appropriate methods for statistical combination of results? | | | | | | | | | Yes | | | No | | | | No meta-analysis conducted | | | | | |
| 12. If meta-analysis was performed, did the review authors assess the potential impact of RoB in individual studies on the results of the meta-analysis or other evidence synthesis? | | | | | | | | | Yes | | | No | | | | No meta-analysis conducted | | | | | |
| 13. Did the review authors account for RoB in individual studies when interpreting/ discussing the results of the review? | | | | | | | | | Yes | | | No | | | | / | | | | | |
| 14. Did the review authors provide a satisfactory explanation for, and discussion of, any heterogeneity observed in the results of the review? | | | | | | | | | Yes | | | No | | | | / | | | | | |
| 15. If they performed quantitative synthesis did the review authors carry out an adequate investigation of publication bias (small study bias) and discuss its likely impact on the results of the review? | | | | | | | | | Yes | | | No | | | | No meta-analysis conducted | | | | | |
| 16. Did the review authors report any potential sources of conflict of interest, including any funding they received for conducting the review? | | | | | | | | | Yes | | | No | | | | / | | | | | |
|  | | | | | | | | | | | | | | | | | | | | | |
| **B. The Critical Appraisal Skills Programme (CASP) Checklist for systematic review (last amended in 2018)**  **Website:** https://casp-uk.net/casp-tools-checklists/ | | | | | | | | | | | | | | | | | | | | | |
| Major Components | | | | | | | Response options | | | | | | | | | | | | | | |
| **Section A: Are the results of the review valid?** | | | | | | | | | | | | | | | | | | | | | |
| 1. Did the review address a clearly focused question? | | | | | | | Yes | | | | | | No | | | | | Can’t Tell | | | |
| 2. Did the authors look for the right type of papers? | | | | | | | Yes | | | | | | No | | | | | Can’t Tell | | | |
| Is it worth continuing? | | | | | | | | | | | | | | | | | | | | | |
| 3. Do you think all the important, relevant studies were included? | | | | | | | Yes | | | | | | No | | | | | Can’t Tell | | | |
| 4. Did the review’s authors do enough to assess quality of the included studies? | | | | | | | Yes | | | | | | No | | | | | Can’t Tell | | | |
| 5. If the results of the review have been combined, was it reasonable to do so? | | | | | | | Yes | | | | | | No | | | | | Can’t Tell | | | |
| **Section B: What are the results?** | | | | | | | | | | | | | | | | | | | | | |
| 6. What are the overall results of the review? | | | | | | |  | | | | | | | | | | | | | | |
| 7. How precise are the results? | | | | | | |  | | | | | | | | | | | | | | |
| **Section C: Will the results help locally?** | | | | | | | | | | | | | | | | | | | | | |
| 8. Can the results be applied to the local population? | | | | | | | Yes | | | | | | No | | | | | Can’t Tell | | | |
| 9. Were all important outcomes considered? | | | | | | | Yes | | | | | | No | | | | | Can’t Tell | | | |
| 10. Are the benefits worth the harms and costs? | | | | | | | Yes | | | | | | No | | | | | Can’t Tell | | | |
|  | | | | | | | | | | | | | | | | | | | | | |
| **C. The Scottish Intercollegiate Guidelines Network (SIGN) Methodology checklist: systematic reviews and meta-analyses (last amended in 2014)**  **Website:** https://www.sign.ac.uk/checklists-and-notes.html | | | | | | | | | | | | | | | | | | | | | |
| Major Components | Response options | | | | | | | | | | | | | | | | | | | | |
| **Section 1: Internal validity** | | | | | | | | | | | | | | | | | | | | | |
| In a well conducted systematic review: | Does this study do it? | | | | | | | | | | | | | | | | | | | | |
| 1.1. The research question is clearly defined and the inclusion/ exclusion criteria must be listed in the paper | Yes | | No | | | | | | | | If no reject | | | | | | / | | | | |
| 1.2. A comprehensive literature search is carried out | Yes | | No | | | | | | | | Not applicable | | | | | | If no reject | | | | |
| 1.3. At least two people should have selected studies | Yes | | No | | | | | | | | Can’t say | | | | | | / | | | | |
| 1.4. At least two people should have extracted data | Yes | | No | | | | | | | | Can’t say | | | | | | / | | | | |
| 1.5. The status of publication was not used as an inclusion criterion | Yes | | No | | | | | | | | / | | | | | | / | | | | |
| 1.6. The excluded studies are listed | Yes | | No | | | | | | | | / | | | | | | / | | | | |
| 1.7. The relevant characteristics of the included studies are provided | Yes | | No | | | | | | | | / | | | | | | / | | | | |
| 1.8. The scientific quality of the included studies was assessed and reported | Yes | | No | | | | | | | | / | | | | | | / | | | | |
| 1.9. Was the scientific quality of the included studies used appropriately? | Yes | | No | | | | | | | | / | | | | | | / | | | | |
| 1.10. Appropriate methods are used to combine the individual study findings | Yes | | No | | | | | | | | Not applicable | | | | | | Can’t say | | | | |
| 1.11. The likelihood of publication bias was assessed appropriately | Yes | | No | | | | | | | | Not applicable | | | | | | / | | | | |
| 1.12. Conflicts of interest are declared | Yes | | No | | | | | | | | / | | | | | | / | | | | |
| **Section 2: Overall assessment of the study** | | | | | | | | | | | | | | | | | | | | | |
| 2.1. What is your overall assessment of the methodological quality of this review? | High quality (++) | | Acceptable (+) | | | | | | | | Low quality (-) | | | | | | Unacceptable – reject 0 | | | | |
| 2.2. Are the results of this study directly applicable to the patient group targeted by this guideline? | Yes | | No | | | | | | | | / | | | | | | / | | | | |
| 2.3. Notes: | | | | | | | | | | | | | | | | | | | | | |
|  | | | | | | | | | | | | | | | | | | | | | |
| **D. The Joanna Briggs Institute (JBI) Critical Appraisal Checklist for systematic reviews and research syntheses (last amended in 2017)**  **Website:** https://joannabriggs.org/critical_appraisal_tools  https://wiki.joannabriggs.org/display/MANUAL/Appendix+10.1+JBI+Critical+Appraisal+Checklist+for+Systematic+reviews+and+Research+Syntheses | | | | | | | | | | | | | | | | | | | | | |
| Major Components | | | | Response options | | | | | | | | | | | | | | | | | |
| 1. Is the review question clearly and explicitly stated? | | | | Yes | | | | | | No | | | | | Unclear | | | | Not applicable | | |
| 2. Were the inclusion criteria appropriate for review question? | | | | Yes | | | | | | No | | | | | Unclear | | | | Not applicable | | |
| 3. Was the search strategy appropriate? | | | | Yes | | | | | | No | | | | | Unclear | | | | Not applicable | | |
| 4. Were the sources and resources used to search for studies adequate? | | | | Yes | | | | | | No | | | | | Unclear | | | | Not applicable | | |
| 5. Were the criteria for appraising studies appropriate? | | | | Yes | | | | | | No | | | | | Unclear | | | | Not applicable | | |
| 6. Was critical appraisal conducted by two or more reviewers independently? | | | | Yes | | | | | | No | | | | | Unclear | | | | Not applicable | | |
| 7. Were there methods to minimize errors in data extraction? | | | | Yes | | | | | | No | | | | | Unclear | | | | Not applicable | | |
| 8. Were the methods used to combine studies appropriate? | | | | Yes | | | | | | No | | | | | Unclear | | | | Not applicable | | |
| 9. Was the likelihood of publication bias assessed? | | | | Yes | | | | | | No | | | | | Unclear | | | | Not applicable | | |
| 10. Were recommendations for policy and/ or practice supported by the reported data? | | | | Yes | | | | | | No | | | | | Unclear | | | | Not applicable | | |
| 11. Were the specific directives for new research appropriate? | | | | Yes | | | | | | No | | | | | Unclear | | | | Not applicable | | |
| Overall appraisal: Include □ Exclude □ Seek further info □ | | | | | | | | | | | | | | | | | | | | | |
|  | | | | | | | | | | | | | | | | | | | | | |
| **E. The National Institutes of Health (NIH) quality assessment tool for systematic reviews and meta-analyses**  **Website:** https://www.nhlbi.nih.gov/health-topics/study-quality-assessment-tools | | | | | | | | | | | | | | | | | | | | | |
| Major Components | | Response options | | | | | | | | | | | | | | | | | | | |
| 1. Is the review based on a focused question that is adequately formulated and described? | | Yes | | | No | | | Cannot Determine/ Not Applicable/ Not Reported | | | | | | | | | | | | | |
| 2. Were eligibility criteria for included and excluded studies predefined and specified? | | Yes | | | No | | | Cannot Determine/ Not Applicable/ Not Reported | | | | | | | | | | | | | |
| 3. Did the literature search strategy use a comprehensive, systematic approach? | | Yes | | | No | | | Cannot Determine/ Not Applicable/ Not Reported | | | | | | | | | | | | | |
| 4. Were titles, abstracts, and full-text articles dually and independently reviewed for inclusion and exclusion to minimize bias? | | Yes | | | No | | | Cannot Determine/ Not Applicable/ Not Reported | | | | | | | | | | | | | |
| 5. Was the quality of each included study rated independently by two or more reviewers using a standard method to appraise its internal validity? | | Yes | | | No | | | Cannot Determine/ Not Applicable/ Not Reported | | | | | | | | | | | | | |
| 6. Were the included studies listed along with important characteristics and results of each study? | | Yes | | | No | | | Cannot Determine/ Not Applicable/ Not Reported | | | | | | | | | | | | | |
| 7. Was publication bias assessed? | | Yes | | | No | | | Cannot Determine/ Not Applicable/ Not Reported | | | | | | | | | | | | | |
| 8. Was heterogeneity assessed? (This question applies only to meta-analyses.) | | Yes | | | No | | | Cannot Determine/ Not Applicable/ Not Reported | | | | | | | | | | | | | |
| **Quality Rating** | | Good | | | Fair | | | Poor | | | | | | | | | | | | | |
| Additional Comments (If Poor, please state why): | | | | | | | | | | | | | | | | | | | | | |
|  | | | | | | | | | | | | | | | | | | | | | |
| **F. The Decision Support Unit (DSU) network meta-analysis (NMA) methodology checklist (last introduced in January 2012)**  **Website:** http://nicedsu.org.uk/technical-support-documents/evidence-synthesis-tsd-series/ | | | | | | | | | | | | | | | | | | | | | |
| Major Components | | | | | | | | | | | | | | | Response options | | | | | | |
| **A. DEFINITION OF THE DECISION PROBLEM** | | | | | | | | | | | | | | | | | | | | | |
| A1. Target population for decision | | | | | | | | | | | | | | | | | | | | | |
| A1.1. Has the target patient population for decision been clearly defined? | | | | | | | | | | | | | | | Yes | | No | | | | Not applicable |
| A2. Comparators | | | | | | | | | | | | | | | | | | | | | |
| A2.1. Decision Comparator Set: Have all the appropriate treatments in the decision been identified? | | | | | | | | | | | | | | | Yes | | No | | | | Not applicable |
| A2.2. Synthesis Comparator Set: Are there additional treatments in the Synthesis Comparator Set, which are not in the Decision Comparator Set? If so, is this adequately justified? | | | | | | | | | | | | | | | Yes | | No | | | | Not applicable |
| A3. Trial inclusion/ exclusion | | | | | | | | | | | | | | | | | | | | | |
| A3.1. Is the search strategy technically adequate and appropriately reported? | | | | | | | | | | | | | | | Yes | | No | | | | Not applicable |
| A3.2. Have all trials involving at least two of the treatments in the Synthesis Comparator Set been included? | | | | | | | | | | | | | | | Yes | | No | | | | Not applicable |
| A3.3. Have all trials reporting relevant outcomes been included? | | | | | | | | | | | | | | | Yes | | No | | | | Not applicable |
| A3.4. Have additional trials been included? If so, is this adequately justified? | | | | | | | | | | | | | | | Yes | | No | | | | Not applicable |
| A4. Treatment Definition | | | | | | | | | | | | | | | | | | | | | |
| A4.1. Are all the treatment options restricted to specific doses and co-treatments, or have different doses and co-treatments been “lumped” together? If the latter, is it adequately justified? | | | | | | | | | | | | | | | Yes | | No | | | | Not applicable |
| A4.2. Are there any additional modelling assumptions? | | | | | | | | | | | | | | | Yes | | No | | | | Not applicable |
| A5. Trial outcomes and scale of measurement chosen for the synthesis | | | | | | | | | | | | | | | | | | | | | |
| A5.1. Where alternative outcomes are available, has the choice of outcome measure used in the synthesis been justified? | | | | | | | | | | | | | | | Yes | | No | | | | Not applicable |
| A5.2. Have the assumptions behind the choice of scale been justified? | | | | | | | | | | | | | | | Yes | | No | | | | Not applicable |
| A6. Patient population: trials with patients outside the target population | | | | | | | | | | | | | | | | | | | | | |
| A6.1. Do some trials include patients outside the target population? If so, is this adequately justified? | | | | | | | | | | | | | | | Yes | | No | | | | Not applicable |
| A6.2. What assumptions are made about the impact, or lack of impact this may have on the relative treatment effects? Are they adequately justified? | | | | | | | | | | | | | | | Yes | | No | | | | Not applicable |
| A6.3. Has an adjustment been made to account for these differences? If so, comment on the adequacy of the evidence presented in support of this adjustment, and on the need for a sensitivity analysis. | | | | | | | | | | | | | | | Yes | | No | | | | Not applicable |
| A7 Patient population: heterogeneity within the target population | | | | | | | | | | | | | | | | | | | | | |
| A7.1. Has there been a review of the literature concerning potential modifiers of treatment effect? | | | | | | | | | | | | | | | Yes | | No | | | | Not applicable |
| A7.2. Are there apparent or potential differences between trials in their patient populations, albeit within the target population? If so, has this been adequately taken into account? | | | | | | | | | | | | | | | Yes | | No | | | | Not applicable |
| A8. Risk of Bias | | | | | | | | | | | | | | | | | | | | | |
| A8.1. Is there a discussion of the biases to which these trials, or this ensemble of trials, are vulnerable? | | | | | | | | | | | | | | | Yes | | No | | | | Not applicable |
| A8.2. If a bias risk was identified, was any adjustment made to the analysis and was this adequately justified? | | | | | | | | | | | | | | | Yes | | No | | | | Not applicable |
| A9. Presentation of the data | | | | | | | | | | | | | | | | | | | | | |
| A9.1. Is there a clear table or diagram showing which data have been included in the base-case analysis? | | | | | | | | | | | | | | | Yes | | No | | | | Not applicable |
| A9.2. Is there a clear table or diagram showing which data have been excluded and why? | | | | | | | | | | | | | | | Yes | | No | | | | Not applicable |
| **B. METHODS OF ANALYSIS AND PRESENTATION OF RESULTS** | | | | | | | | | | | | | | | | | | | | | |
| B1. Meta-analytic methods | | | | | | | | | | | | | | | | | | | | | |
| B1.1. Is the statistical model clearly described? | | | | | | | | | | | | | | | Yes | | No | | | | Not applicable |
| B1.2. Has the software implementation been documented? | | | | | | | | | | | | | | | Yes | | No | | | | Not applicable |
| B2. Heterogeneity in the relative treatment effects | | | | | | | | | | | | | | | | | | | | | |
| B2.1. Have numerical estimates been provided of the degree of heterogeneity in the relative treatment effects? | | | | | | | | | | | | | | | Yes | | No | | | | Not applicable |
| B2.2. Has a justification been given for choice of random or fixed effect models? Should sensitivity analyses be considered? | | | | | | | | | | | | | | | Yes | | No | | | | Not applicable |
| B2.3. Has there been adequate response to heterogeneity? | | | | | | | | | | | | | | | Yes | | No | | | | Not applicable |
| B2.4. Does the extent of unexplained variation in relative treatment effects threaten the robustness of conclusions? | | | | | | | | | | | | | | | Yes | | No | | | | Not applicable |
| B2.5. Has the statistical heterogeneity between baseline arms been discussed? | | | | | | | | | | | | | | | Yes | | No | | | | Not applicable |
| B3. Baseline model for trial outcomes | | | | | | | | | | | | | | | | | | | | | |
| B3.1. Are baseline effects and relative effects estimated in the same model? If so, has this been justified? | | | | | | | | | | | | | | | Yes | | No | | | | Not applicable |
| B3.2. Has the choice of studies to inform the baseline model been explained? | | | | | | | | | | | | | | | Yes | | No | | | | Not applicable |
| B4. Presentation of results of analyses of trial data | | | | | | | | | | | | | | | | | | | | | |
| B4.1. Are the relative treatment effects (relative to a placebo or “standard” comparator) tabulated, alongside measures of between-study heterogeneity if a RE model is used? | | | | | | | | | | | | | | | Yes | | No | | | | Not applicable |
| B4.2. Are the absolute effects on each treatment, as they are used in the cost effectiveness analysis (CEA), reported? | | | | | | | | | | | | | | | Yes | | No | | | | Not applicable |
| B5. Synthesis in other parts of the natural history model | | | | | | | | | | | | | | | | | | | | | |
| B5.1. Is the choice of data sources to inform the other parameters in the natural history model adequately described and justified? | | | | | | | | | | | | | | | Yes | | No | | | | Not applicable |
| B5.2. In the natural history model, can the longer-term differences between treatments be explained by their differences on randomised trial outcomes? | | | | | | | | | | | | | | | Yes | | No | | | | Not applicable |
| **C. ISSUES SPECIFIC TO NETWORK SYNTHESIS** | | | | | | | | | | | | | | | | | | | | | |
| C1. Adequacy of information on model specification and software implementation | | | | | | | | | | | | | | | | | | | | | |
| C2. Multi-arm trials | | | | | | | | | | | | | | | | | | | | | |
| C2.1. If there are multi-arm trials, have the correlations between the relative treatment effects been taken into account? | | | | | | | | | | | | | | | Yes | | No | | | | Not applicable |
| C3. Connected and disconnected networks | | | | | | | | | | | | | | | | | | | | | |
| C3.1. Is the network of evidence based on randomised trials connected? | | | | | | | | | | | | | | | Yes | | No | | | | Not applicable |
| C4. Inconsistency | | | | | | | | | | | | | | | | | | | | | |
| C4.1. How many inconsistencies could there be in the network? | | | | | | | | | | | | | | | Yes | | No | | | | Not applicable |
| C4.2. Are there any a priori reasons for concern that inconsistency might exist, due to systematic clinical differences between the patients in trials comparing treatments A and B, and the patients in trials comparing treatments A and C, etc? | | | | | | | | | | | | | | | Yes | | No | | | | Not applicable |
| C4.3. Have adequate checks for inconsistency been made? | | | | | | | | | | | | | | | Yes | | No | | | | Not applicable |
| C4.4. If inconsistency was detected, what adjustments were made to the analysis, and how was this justified? | | | | | | | | | | | | | | | Yes | | No | | | | Not applicable |
| **D. EMBEDDING THE SYNTHESIS IN A PROBABILISTIC** **COST EFFECTIVENESS ANALYSIS** | | | | | | | | | | | | | | | | | | | | | |
| D1. Uncertainty Propagation | | | | | | | | | | | | | | | | | | | | | |
| D1.1. Has the uncertainty in parameter estimates been propagated through the CEA model? | | | | | | | | | | | | | | | Yes | | No | | | | Not applicable |
| D2. Correlations | | | | | | | | | | | | | | | | | | | | | |
| D2.1. Are there correlations between parameters? If so, have the correlations been propagated through the CEA model? | | | | | | | | | | | | | | | Yes | | No | | | | Not applicable |
|  | | | | | | | | | | | | | | | | | | | | | |
| **G. The Risk of Bias in Systematic Review (ROBIS) tool (last released in 2014)**  **Website:** www.robis-tool.info | | | | | | | | | | | | | | | | | | | | | |
| Major Components | | | | | | Response options | | | | | | | | | | | | | | | |
| **DOMAIN 1: STUDY ELIGIBILITY CRITERIA**  Describe the study eligibility criteria, any restrictions on eligibility and whether there was evidence that objectives and eligibility criteria were pre-specified: | | | | | | | | | | | | | | | | | | | | | |
| 1.1. Did the review adhere to pre-defined objectives and eligibility criteria? | | | | | | Yes/ Probably yes | | | | | | | | No/ Probably no | | | | | | No information | |
| 1.2. Were the eligibility criteria appropriate for the review question? | | | | | | Yes/ Probably yes | | | | | | | | No/ Probably no | | | | | | No information | |
| 1.3. Were eligibility criteria unambiguous? | | | | | | Yes/ Probably yes | | | | | | | | No/ Probably no | | | | | | No information | |
| 1.4. Were all restrictions in eligibility criteria based on study characteristics appropriate (e.g. date, sample size, study quality, outcomes measured)? | | | | | | Yes/ Probably yes | | | | | | | | No/ Probably no | | | | | | No information | |
| 1.5. Were any restrictions in eligibility criteria based on sources of information appropriate (e.g. publication status or format, language, availability of data)? | | | | | | Yes/ Probably yes | | | | | | | | No/ Probably no | | | | | | No information | |
| Concerns regarding specification of study eligibility criteria | | | | | | High | | | | | | | | Low | | | | | | Unclear | |
| Rationale for concern: | | | | | | | | | | | | | | | | | | | | | |
| **DOMAIN 2: IDENTIFICATION AND SELECTION OF STUDIES**  Describe methods of study identification and selection (e.g. number of reviewers involved): | | | | | | | | | | | | | | | | | | | | | |
| 2.1. Did the search include an appropriate range of databases/electronic sources for published and unpublished reports? | | | | | | Yes/ Probably yes | | | | | | | | No/ Probably no | | | | | | No information | |
| 2.2. Were methods additional to database searching used to identify relevant reports? | | | | | | Yes/ Probably yes | | | | | | | | No/ Probably no | | | | | | No information | |
| 2.3. Were the terms and structure of the search strategy likely to retrieve as many eligible studies as possible? | | | | | | Yes/ Probably yes | | | | | | | | No/ Probably no | | | | | | No information | |
| 2.4. Were restrictions based on date, publication format, or language appropriate? | | | | | | Yes/ Probably yes | | | | | | | | No/ Probably no | | | | | | No information | |
| 2.5. Were efforts made to minimise error in selection of studies? | | | | | | Yes/ Probably yes | | | | | | | | No/ Probably no | | | | | | No information | |
| Concerns regarding methods used to identify and/or select studies | | | | | | High | | | | | | | | Low | | | | | | Unclear | |
| Rationale for concern: | | | | | | | | | | | | | | | | | | | | | |
| **DOMAIN 3: DATA COLLECTION AND STUDY APPRAISAL**  Describe methods of data collection, what data were extracted from studies or collected through other means, how risk of bias was assessed (e.g. number of reviewers involved) and the tool used to assess risk of bias: | | | | | | | | | | | | | | | | | | | | | |
| 3.1. Were efforts made to minimise error in data collection? | | | | | | Yes/ Probably yes | | | | | | | | No/ Probably no | | | | | | No information | |
| 3.2. Were sufficient study characteristics available for both review authors and readers to be able to interpret the results? | | | | | | Yes/ Probably yes | | | | | | | | No/ Probably no | | | | | | No information | |
| 3.3. Were all relevant study results collected for use in the synthesis? | | | | | | Yes/ Probably yes | | | | | | | | No/ Probably no | | | | | | No information | |
| 3.4. Was risk of bias (or methodological quality) formally assessed using appropriate criteria? | | | | | | Yes/ Probably yes | | | | | | | | No/ Probably no | | | | | | No information | |
| 3.5. Were efforts made to minimise error in risk of bias assessment? | | | | | | Yes/ Probably yes | | | | | | | | No/ Probably no | | | | | | No information | |
| Concerns regarding methods used to collect data and appraise studies | | | | | | High | | | | | | | | Low | | | | | | Unclear | |
| Rationale for concern: | | | | | | | | | | | | | | | | | | | | | |
| **DOMAIN 4: SYNTHESIS AND FINDINGS**  Describe synthesis methods: | | | | | | | | | | | | | | | | | | | | | |
| 4.1 Did the synthesis include all studies that it should? | | | | | |  | | | | | | | |  | | | | | |  | |
| 4.2 Were all pre-defined analyses reported or departures explained? | | | | | | Yes/ Probably yes | | | | | | | | No/ Probably no | | | | | | No information | |
| 4.3 Was the synthesis appropriate given the nature and similarity in the research questions, study designs and outcomes across included studies? | | | | | | Yes/ Probably yes | | | | | | | | No/ Probably no | | | | | | No information | |
| 4.4 Was between-study variation (heterogeneity) minimal or addressed in the synthesis? | | | | | | Yes/ Probably yes | | | | | | | | No/ Probably no | | | | | | No information | |
| 4.5 Were the findings robust, e.g. as demonstrated through funnel plot or sensitivity analyses? | | | | | | Yes/ Probably yes | | | | | | | | No/ Probably no | | | | | | No information | |
| 4.6 Were biases in primary studies minimal or addressed in the synthesis? | | | | | | Yes/ Probably yes | | | | | | | | No/ Probably no | | | | | | No information | |
| Concerns regarding the synthesis and findings | | | | | | High | | | | | | | | Low | | | | | | Unclear | |
| Rationale for concern: | | | | | | | | | | | | | | | | | | | | | |
| **RISK OF BIAS IN THE REVIEW**  Describe whether conclusions were supported by the evidence: | | | | | | | | | | | | | | | | | | | | | |
| A. Did the interpretation of findings address all of the concerns identified in Domains 1 to 4? | | | | | | Yes/ Probably yes | | | | | | | | No/ Probably no | | | | | | No information | |
| B. Was the relevance of identified studies to the review's research question appropriately considered? | | | | | | Yes/ Probably yes | | | | | | | | No/ Probably no | | | | | | No information | |
| C. Did the reviewers avoid emphasizing results on the basis of their statistical significance? | | | | | | Yes/ Probably yes | | | | | | | | No/ Probably no | | | | | | No information | |
| Risk of bias in the review: | | | | | | High | | | | | | | | Low | | | | | | Unclear | |
| Rationale for risk: | | | | | | | | | | | | | | | | | | | | | |
|  | | | | | | | | | | | | | | | | | | | | | |
| H. The Appraisal of Guidelines for Research and Evaluation II (AGREE II) instrument (last released in 2009)  **Website:** http://www.agreetrust.org/ | | | | | | | | | | | | | | | | | | | | | |
| 1. The overall objective(s) of the guideline is (are) specifically described  2. The health question(s) covered by the guideline is (are) specifically described  3. The population (patients, public, etc.) to whom the guideline is meant to apply is specifically described  4. The guideline development group includes individuals from all the relevant professional groups  5. The views and preferences of the target population (patients, public, etc.) have been sought  6. The target users of the guideline are clearly defined  7. Systematic methods were used to search for evidence  8. The criteria for selecting the evidence are clearly described  9. The strengths and limitations of the body of evidence are clearly described  10. The methods for formulating the recommendations are clearly described  11. The health benefits, side effects, and risks have been considered in formulating the recommendations  12. There is an explicit link between the recommendations and the supporting evidence  13. The guideline has been externally reviewed by experts prior to its publication  14. A procedure for updating the guideline is provided  15. A procedure for updating the guideline is provided  16. The different options for management of the condition or health issue are clearly presented  17. Key recommendations are easily identifiable  18. The guideline provides advice and/or tools on how the recommendations can be put into practice  19. The guideline describes facilitators and barriers to its application  20. The potential resource implications of applying the recommendations have been considered  21. The guideline presents monitoring and/ or auditing criteria  22. The views of the funding body have not influenced the content of the guideline  23. Competing interests of guideline development group members have been recorded and addressed | | | | All AGREE II items are rated on a 7-point scale.  Score of 1 means "Strongly Disagree", while score of 7 means "Strongly Agree".  A score between 2 and 6 is assigned when the reporting of the AGREE II item does not meet the full criteria or considerations.  A score is assigned depending on the completeness and quality of reporting. Scores increase as more criteria are met and considerations addressed.  The “How to Rate” section for each item includes details about assessment criteria and considerations specific to the item. | | | | | | | | | | | | | | | | | |
